# Effective Membrane Permeabilization of Methicillin-Resistant *Staphylococcus aureus* by Prenylated Phenolics

**DOI:** 10.1021/acs.jnatprod.5c00540

**Published:** 2025-08-19

**Authors:** Janniek H. Ritsema, Nynke I. Kramer, Wouter J. C. de Bruijn, Sarah van Dinteren, Maurice C. R. Franssen, Jean-Paul Vincken, Carla Araya-Cloutier

**Affiliations:** † Laboratory of Food Chemistry, 4508 Wageningen University, Bornse Weilanden 9, 6708 WG Wageningen, Netherlands; ‡ Toxicology Chair Group, Wageningen University, Stippeneng 4, 6708 WE Wageningen, Netherlands; § Laboratory of Organic Chemistry, Wageningen University, Stippeneng 4, 6708 WE Wageningen, Netherlands

## Abstract

Prenylated phenolics are plant-derived compounds with antimicrobial activity against methicillin-resistant *Staphylococcus aureus* (MRSA), acting by targeting membranes resulting in fast permeabilization. Studies quantifying their membrane permeabilization capacity are lacking, limiting our understanding of the structural properties driving this effect. This study evaluated antimicrobial activity and permeabilization efficacy of 36 C- and O-prenylated phenolics, including 11 C- and O-prenylated phenolics chemically synthesized for this study. Minimum inhibitory concentrations (MICs) were obtained using the broth microdilution assay. Membrane permeabilization was measured by propidium iodide uptake using fluorescence spectrometry and microscopy. The most potent MRSA permeabilizers were luteone (**29**) and neobavaisoflavone (**22**), with EC_10_ of 27 ± 7 and 28 ± 8 μg mL^–1^, respectively. Diprenylated phenolics showed a strong negative correlation between permeabilization and their hydrophobic-to-polar surface area ratio (*r*_pearson_ = 0.88). For monoprenylated phenolics, prenyl configuration (chain) and molecular shape (globular) were important for effective permeabilization. Interestingly, potency of antimicrobial prenylated phenolics (MIC ≤ 50 μg mL^–1^) was not correlated to permeabilization potency, suggesting other mechanisms of action in addition to membrane permeabilization. These quantitative findings on membrane permeabilization by prenylated phenolics contribute to our mechanistic understanding of how these compounds can inhibit microbial growth.

## Graphical abstract

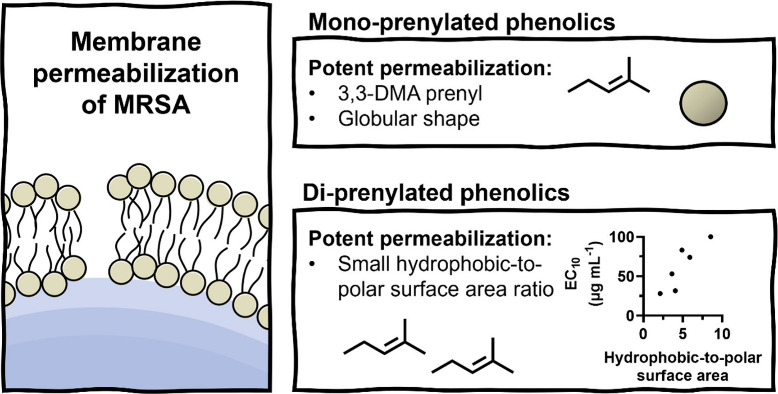

Antimicrobial resistance is one of the biggest threats to society, endangering both human and animal health.^1^ Among drug-resistant bacteria, methicillin-resistant *Staphylococcus aureus* (MRSA) is the second leading pathogen for resistance-associated deaths, being responsible for more than 100 000 deaths in 2019 worldwide.^2^ MRSA is intrinsically resistant to all β-lactam antibiotics (including penicillins, cephalosporins, and carbapenems), but also tends to develop resistance to other, unrelated antibiotics.^3^ To combat antimicrobial resistance, novel and effective alternatives to traditional antimicrobial agents with different mechanisms of action need to be developed.^4^

Prenylated phenolics are defense secondary metabolites produced by plants of the Leguminosae family upon stress.^5–8^ They possess anti-MRSA activity, with minimum inhibitory concentrations (MICs) ranging between 2 and 50 μg mL^–1^.^9–11^ Prenylation increases the hydrophobicity of a molecule, which enhances affinity toward biological targets, such as membranes and proteins.^12^ The structural diversity of prenylated phenolics in plants is large: different phenolic subclasses (e.g., flavones, isoflavans, and isoflavones) exist and the number, position, and configuration of prenyl groups, as well as the presence of other substituents (e.g., hydroxyl and methoxy), can vary. Phenolics can be prenylated on the carbon skeleton or on a hydroxyl group, referred to as C- or O-prenylation, respectively. Most natural prenylated phenolics are C-prenylated. O-Prenylation is less common in nature.^13^ The prenyl configuration most commonly present in nature is a 3,3-dimethylallyl (3,3-DMA) chain or a 2,2-dimethylpyran (2,2-DMP) ring.^13^

C-Prenylated phenolics have been studied extensively for their antimicrobial potency allowing the development of structure–activity relationships (SARs) and quantitative SARs (QSARs). In general, the addition of a second prenyl group increases activity against MRSA,^9,14^ and chain prenylated (3,3-DMA) phenolics are more active than ring prenylated (2,2-DMP) phenolics.^9,11,15^ However, the extensive structural diversity of prenylated phenolics pose a challenge in establishing clear SARs applicable to all phenolic subclasses. To address this issue, Kalli et al.^9^ developed a QSAR model to gain insights into molecular properties of C-prenylated phenolics underlying anti-MRSA activity. Increased anti-MRSA activity was positively linked to (i) hydrophobic volume, (ii) balanced hydrophilic surface areas, and (iii) the presence of formal negative charge.^9^

Some C-prenylated phenolics permeabilize the cytoplasmic membrane of MRSA.^16–20^ Permeabilization is commonly assessed by monitoring the uptake of propidium iodide (PI), a fluorescent probe, through fluorescence microscopy or spectrometry. Propidium is a membrane-impermeable doubly charged cation and can only enter the cell and intercalate with DNA when the cytoplasmic membrane is disrupted.^21^ Most studies have predominantly focused on one or two C-prenylated phenolics, highlighting their ability to permeabilize the cytoplasmic membrane.^16,19,22,23^ Unfortunately, differences in experimental conditions (e.g., microorganism studied and initial inoculum size) make it impossible to compare permeabilization across these studies. Kalli et al.^16^ found that chain prenylated wighteone (isoflavone) permeabilized membranes faster than ring prenylated glabridin (isoflavan) in the yeast *Zygosaccharomyces parabailii*. Sun et al.^20^ showed that eight C-prenylated phenolics permeabilized the cytoplasmic membrane of MRSA, although the relationships between permeabilization and structural features were not explored. Furthermore, Araya-Cloutier et al.^17^ studied the permeabilization of the cytoplasmic membrane of *Listeria monocytogenes* by 13 C-prenylated phenolics. Several diprenylated phenolics that were very potent antimicrobials (MIC against *L. monocytogenes* between 6 and 15 μg mL^–1^), however, showed no or poor membrane permeabilization at tested concentration (20 μg mL^–1^), and a significant negative correlation between the relative hydrophobic molecular surface area and permeabilization was found.^17^

In addition to permeabilization of whole cells, C-prenylated phenolics have also been studied for their ability to permeabilize model membranes (liposomes).^24,25^ Permeabilization of liposomes by two mono-C-prenylated phenolics (glabridin and wighteone) confirmed the lipid bilayer as primary target.^25^ Furthermore, *in silico* molecular dynamics simulations showed that wighteone and glabridin interacted just below the phospholipid headgroups.^25^ Wesołowska et al.^24^ showed that the localization of three mono-C-prenylated phenolics within liposomes was influenced by the compounds’ hydrophobicity and shape: more hydrophobic or less globular prenylated phenolics interacted deeper in the model membranes. To conclude, previous research indicates variation in permeabilization potency for antimicrobial prenylated phenolics, depending on their structure, suggesting potential differences in their mechanism of action.

In contrast to C-prenylated phenolics, O-prenylated phenolics have been poorly studied for their antimicrobial properties and membrane permeabilization capacity. Two O-prenylated genistein derivatives, 5-hydroxy-7-methoxy-4′-*O*-(3-methylbut-2-enyl)­isoflavone and 7,4′-*O*-diprenylgenistein, have been tested against various Gram-positive bacteria but were inactive (MIC > 128 μg mL^–1^).^26^ The O-prenylated chalcone 4-hydroxycordoin was inactive (MIC > 200 μg mL^–1^) against the yeast *Candida albicans*, but inhibited biofilm formation at 20 μg mL^–1^.^27^ O-prenylated naringenin (MIC > 50 μg mL^–1^) and genistein (MIC > 25 μg mL^–1^) derivatives were inactive against *Saccharomyces cerevisiae*, while the O-prenylated stilbenoid 4′-*O*-prenylresveratrol inhibited *S. cerevisiae* growth (MIC 12.5 μg mL^–1^).^28^ Considering that hydrophilicity is important for the antimicrobial activity of C-prenylated phenolics,^9,29^ O-prenylation might be less effective than C-prenylation. However, if sufficient hydroxyl groups remain present after O-prenylation, they could possibly possess anti-MRSA activity. To the best of our knowledge, it is unknown whether O-prenylated phenolics permeabilize the cytoplasmic membrane. Furthermore, no systematic study has been performed to establish SARs for permeabilization of bacterial membranes by prenylated phenolics.

In view of these knowledge gaps, this study aimed to systematically evaluate the antimicrobial activity and permeabilization capacity of 36 C- and O-prenylated phenolics (of which 14 are diprenylated, Figure 1) against MRSA, and to establish SARs for membrane permeabilization. Permeabilization capacity was studied at various concentrations, and effective concentrations (ECs) were extracted from concentration–response curves. We hypothesized that O-prenylated phenolics will possess antimicrobial activity provided they are sufficiently hydrophilic. Furthermore, based on our previous work,^17^ we hypothesized that a large relative hydrophobic surface area of prenylated phenolics decreases permeabilization of MRSA membranes.

**Figure 1:**
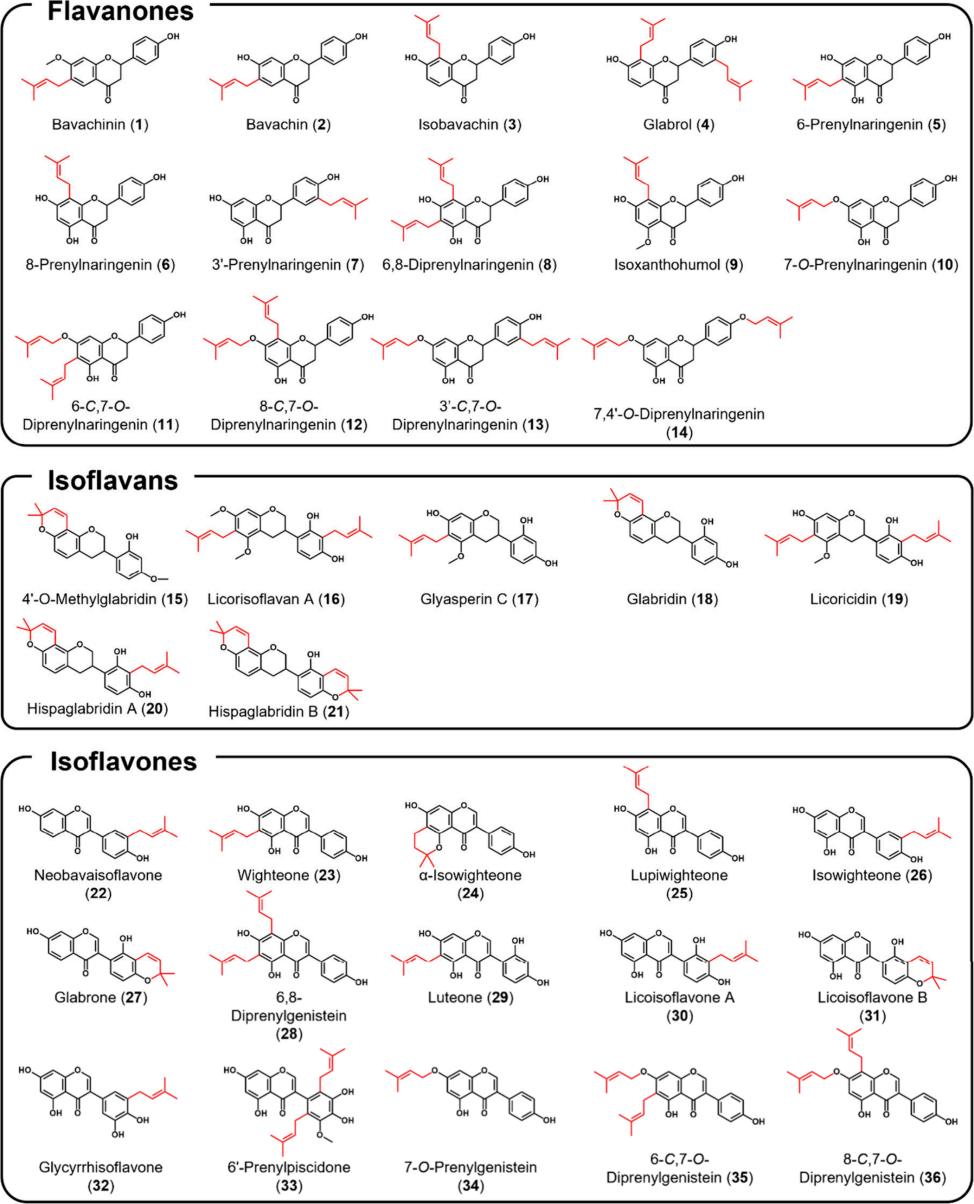
Prenylated phenolics (flavanones, isoflavans, and isoflavones) included in this study (prenyl group in red). Prenyl configurations in our collection of prenylated phenolics were 3,3-dimethylallyl (3,3-DMA) chain [e.g., bavachinin (**1**)], 2,2-dimethylpyran (2,2-DMP) ring [e.g., 4′-*O*-methylglabridin (**15**)], or 2,2-dimethyl-3,4-dihydro-2*H*-pyran [α-isowighteone (**24**)].

## Results and Discussion

### Anti-MRSA Activity of Prenylated Phenolics

We first examined the antimicrobial activity of C- and O-prenylated phenolics against MRSA. Understanding antimicrobial activity was essential, as this provided the foundation for studying the underlying mechanisms of antimicrobial action, in particular membrane permeabilization. The antimicrobial activity of several prenylated phenolics (compounds **4**, **5**, **15**, **18**, **20**, **21**, **22**, **23**, **25**, **26**, **28**, **29**, and **30**) included in this study had been previously established using the same bacterial strain (MRSA 18HN) and experimental conditions at our laboratory.^9^ All other prenylated phenolics, for which antimicrobial activity against MRSA 18HN had not been reported before, were tested in this study. The minimum inhibitory concentrations (MICs) and minimum bactericidal concentrations (MBCs) of all prenylated phenolics (including MIC and MBC values reported by Kalli et al.^9^) are shown in Table 1. Furthermore, Table 1 also shows the log reduction of viable cells at minimum inhibitory concentration. Log reductions provide a more thorough understanding of antimicrobial efficacy, indicating the extent of bacterial killing achieved at MIC.

**Table 1:** MIC and MBC in μg mL^–1^ and μM between Square Brackets of Prenylated Phenolics and Positive Controls (Vancomycin and Bithionol) against MRSA 18HN^a^

compound	MIC in μg mL^–1^ (μM)	log reduction in CFU mL^–1^ (average ± st. dev)	MBC in μg mL^–1^ (μM)
Flavanones			
bavachinin (**1**)	>50^a^ [>148]	n.a.	>50 [>148]
bavachin (**2**)	50 [154]	0.2 ± 0.2	>50 [>154]
isobavachin (**3**)	>50 [>154]	n.a.	>50 [>154]
glabrol (**4**)	9 [24]^e^	n.r.	19 [48]^e^
6-prenylnaringenin (**5**)	38 [110]^e^	n.r.	44 [129]^e^
8-prenylnaringenin (**6**)	13 < MIC ≤ 25 [37 < MIC ≤ 73]^b^	4.3 ± 0.6^c^	25 [73]
3′-prenylnaringenin (**7**)	>50 [>147]	n.a.	>50 [>147]
6,8-diprenylnaringenin (**8**)	6 < MIC ≤ 13 [15 < MIC ≤ 31]	4.6 ± 0.1	13 [31]
isoxanthohumol (**9**)	>50 [>141]	–0.8 ± 0.2	>50 [>141]
7-*O*-prenylnaringenin (**10**)	6 < MIC ≤ 13 [18 < MIC ≤ 37]	4.2 ± 0.7	13 [37]
6-*C*,7-*O*-diprenylnaringenin (**11**)	>50 [>122]	n.a.	>50 [>122]
8-*C*,7-*O*-diprenylnaringenin (**12**)	>50 [>122]	n.a.	>50 [>122]
3′-*C*,7-*O*-diprenylnaringenin (**13**)	>50 [>122]	n.a.	>50 [>122]
7,4′-*O*-diprenylnaringenin (**14**)	>50 [>122]	n.a.	>50 [>122]
Isoflavans			
4′-*O*-methylglabridin (**15**)	10 [30]^e^	n.r.	23 [66]^e^
licorisoflavan A (**16**)	>50 [>114]	n.a.	>50 [>114]
glyasperin C (**17**)	25 [70]	1.3 ± 0.3	50 [140]
glabridin (**18**)	13 [39]^e^	n.r.	19 [58]^e^
licoricidin (**19**)	13 [29]	2.0 ± 2.0	25 [59]
hispaglabridin A (**20**)	44 [111]^e^	n.r.	44 [111]^e^
hispaglabridin B (**21**)	>100 [>256]^e^	n.r.	>100 [>256]^e^
Isoflavones			
neobavaisoflavone (**22**)	38 [116]^e^	n.r.	50 [155]^e^
wighteone (**23**)	16 [46]^e^	n.r.	22 [65]^e^
α-isowighteone (**24**)	>50 [>148]	n.a.	>50 [>148]
lupiwighteone (**25**)	>100 [>296]^e^	n.r.	>100 [>296]^e^
isowighteone (**26**)	22 [65]^e^	n.r.	34 [102]^e^
glabrone (**27**)	25 [74]	1.2 ± 0.5	50 [149]
6,8-diprenylgenistein (**28**)	9 [23]^e^	n.r.	16 [38]^e^
luteone (**29**)	25 [71]^e^	n.r.	44 [123]^e^
licoisoflavone A (**30**)	25 [71]^e^	n.r.	50 [141]^e^
licoisoflavone B (**31**)	13 [35]	2.3 ± 1.9	25 [71]
glycyrrhisoflavone (**32**)	25 < MIC ≤ 50 [71 < MIC ≤ 141]	4.6 ± 0.3	50 [141]
6′-prenylpiscidone (**33**)	25 [55]	2.2 ± 1.0	50 [110]
7-*O*-prenylgenistein (**34**)	>50 [>148]	n.a.	>50 [>148]
6-*C*,7-*O*-diprenylgenistein (**35**)	>50 [>123]	n.a.	>50 [>123]
8-*C*,7-*O*-diprenylgenistein (**36**)	>50 [>123]	n.a.	>50 [>123]
Positive Controls^d^			
vancomycin	<2 [<1]	3.9 ± 0.5	2 [1]
bithionol	2 [4]	1.6 ± 0.5	3 [9]

a The log reduction at the MIC is based on the average cell count of the biological replicates. Abbreviations: n.a., not applicable; n.r., not reported; and st. dev., standard deviation. Structures of prenylated phenolics can be found in Figure 1.

b The highest tested concentration was 50 μg mL^–1^.

c A MIC range, e.g., 13 < MIC ≤ 25 [37 > MIC ≤ 73], indicated that at the lower concentration the cell count was higher than the initial inoculum size + 0.5 log_10_ CFU mL^–1^ (i.e., MIC was not reached), but the higher concentration resulted in >99.9% cell inactivation (i.e., MBC). As no concentration was tested in between, the definite MIC was not obtained.

d When no definite MIC was obtained and a range is given, the log reduction for the highest concentration is shown.

e Positive control vancomycin was tested at 2 μg mL^–1^, and bithionol was tested at 2–25 μg mL^–1^,

f MIC or MBC was reported by Kalli et al.,^9^ and highest tested concentration was 100 μg mL^–1^.

The most active prenylated phenolics were glabrol (**4**), 6,8-diprenylnaringenin (**8**), 7-*O*-prenylnaringenin (**10**), 4′-*O*-methylglabridin (**15**), glabridin (**18**), licoricidin (**19**), 6,8-diprenylgenistein (**28**), and licoisoflavone B (**31**) (MIC ≤ 13 μg mL^–1^). Fifteen compounds were inactive at the highest tested concentration (MIC > 50 μg mL^–1^). The growth delay (GD) is reported in Table S3 for the inactive compounds tested in this research. The GD provides insights into possible subinhibitory effects that slow down bacterial growth.

Of the eight O-prenylated phenolics tested for their antimicrobial activity against MRSA, the monoprenylated flavanone 7-*O*-prenylnaringenin (**10**) was the only compound that showed activity (6 < MIC ≤ 13 μg mL^–1^). It showed higher activity than its C-prenylated analogues (compounds **5** and **6**, MIC > 13 μg mL^–1^). In contrast, the O-prenylated isoflavone 7-*O*-prenylgenistein (**34**) was inactive (MIC > 50 μg mL^–1^).

The antimicrobial action of prenylated phenolics is mainly due to their ability to interact with and disrupt the hydrophobic cytoplasmic membrane.^16–20,30^ A previous QSAR study showed that anti-MRSA activity is positively linked to (i) hydrophobic volume, (ii) balanced hydrophilic surface areas, and (iii) formal negative charges.^9^ It is important to highlight that QSAR models typically predict activity based on the combination of molecular descriptors. Furthermore, this QSAR study only included C-prenylated phenolics, and it is unknown whether these findings are applicable to the anti-MRSA activity of O-prenylated phenolics.^9^

To understand the effect of O-prenylation in comparison to C-prenylation on the molecular properties and antimicrobial activity of these compounds, several molecular descriptors were calculated; i.e., the neutral fraction (% of molecular species in undissociated form), hydrophobicity (log *D*), and solubility (aqueous solubility) (Table S4). More specifically, the calculated molecular properties of O-prenylated flavanones and isoflavones and their C-prenylated counterparts were compared in Figure S60. These properties are important indicators of a compound’s ability to interact with hydrophobic targets under physiological conditions.^31,32^ Compared to C-prenylation, O-prenylation results in the loss of one free hydroxyl group and in a larger neutral fraction at pH 7.3 for both isoflavones and flavanones (Figure S60A).

O-Prenylated flavanones are mainly neutral (≥90% undissociated, see Figure S60A) and mostly inactive. The only O-prenylated compound with antimicrobial activity, 7-*O*-prenylnaringenin (**10**, 6 < MIC ≤ 13 μg mL^–1^), was more active than its mono-C-prenylated analogues (**5**, **6**, and **7**, MIC > 13 μg mL^–1^), but showed similar hydrophobicity and water solubility (Figure S60B and C), suggesting that the increased antimicrobial activity of 7-*O*-prenylnaringenin (**10**) can be attributed to its decreased ability to dissociate.

O-Prenylated isoflavones are more neutral (**34**, **35**, and **36**, ≤ 43% undissociated) and more hydrophobic compared to C-prenylated isoflavones (Figure S60A and B). The lack of anti-MRSA activity at 50 μg mL^–1^ of all tested O-prenylated isoflavones implies that neither cytosolic or effective membrane antimicrobial action occurred, despite their increased hydrophobicity and decreased ability to dissociate. Potentially, the free hydroxyl group at C7 is crucial for the antimicrobial activity of isoflavones.

To conclude, the most potent antimicrobial prenylated phenolics were glabrol (**4**), 6,8-diprenylnaringenin (**8**), 7-*O*-prenylnaringenin (**10**), glabridin (**18**), licoricidin (**19**), 6,8-diprenylgenistein (**28**), and licoisoflavone B (**31**) (MIC ≤ 13 μg mL^–1^). The only O-prenylated phenolic with anti-MRSA activity and also one of the most potent antimicrobial compounds overall was 7-*O*-prenylnaringenin (**10**). However, further research is needed to establish robust SARs for the antimicrobial activity of O-prenylated phenolics.

### Inhibition and Membrane Permeabilization of MRSA by Prenylated Phenolics

#### Cell Viability and Membrane Permeabilization of MRSA Treated with Wighteone and 6,8-Diprenylgenistein

Prior to quantifying the effective concentrations (EC) for membrane permeabilization of the entire collection of prenylated phenolics, a subset of compounds was studied using fluorescence spectrometry and cells were visualized using fluorescence microscopy. Membrane permeabilization was assessed for wighteone (**23**) and 6,8-diprenylgenistein (**28**), alongside the positive control, bithionol. Wighteone (**23**) and 6,8-diprenylgenistein (**28**), which differ by one prenyl group, were selected because they showed very different membrane permeabilization in *L. monocytogenes* at 20 μg mL^–1^.^18^ Bithionol, a fungicidal and anthelmintic drug, was included as positive control, as it is known to permeabilize the cytoplasmic membrane of Gram-positive bacteria, including MRSA.^33–35^ Fluorescence microscopy was also combined with viable cell counting to get insight into the number of viable cells at the conditions used during assessment of permeabilization.

Figure 2A shows membrane permeabilization of MRSA by bithionol, wighteone (**23**), and 6,8-diprenylgenistein (**28**) measured by fluorescence spectrometry. Bithionol was the most potent permeabilizer, followed by wighteone (**23**) and finally 6,8-diprenylgenistein (**28**). The results of wighteone (**23**) and 6,8-diprenylgenistein (**28**) were consistent with previous research, where wighteone (**23**) showed higher PI uptake in *L. monocytogenes* than 6,8-diprenylgenistein (**28**) at 20 μg mL^–1^.^18^ For fluorescence microscopy and viable cell counting, wighteone (**23**) and 6,8-diprenylgenistein (**28**) were tested at a concentration of 50 μg mL^–1^, as at this concentration permeabilization was observed for wighteone (**23**), but not for 6,8-diprenylgenistein (**28**) (Figure 2A). The positive control, bithionol, was included at a concentration of 25 μg mL^–1^. Fluorescence microscopy images were taken after 5 min (Figure S58) and 60 min (Figure 2B) of treatment. Viable cell counts are shown in Figure 2C.

**Figure 2:**
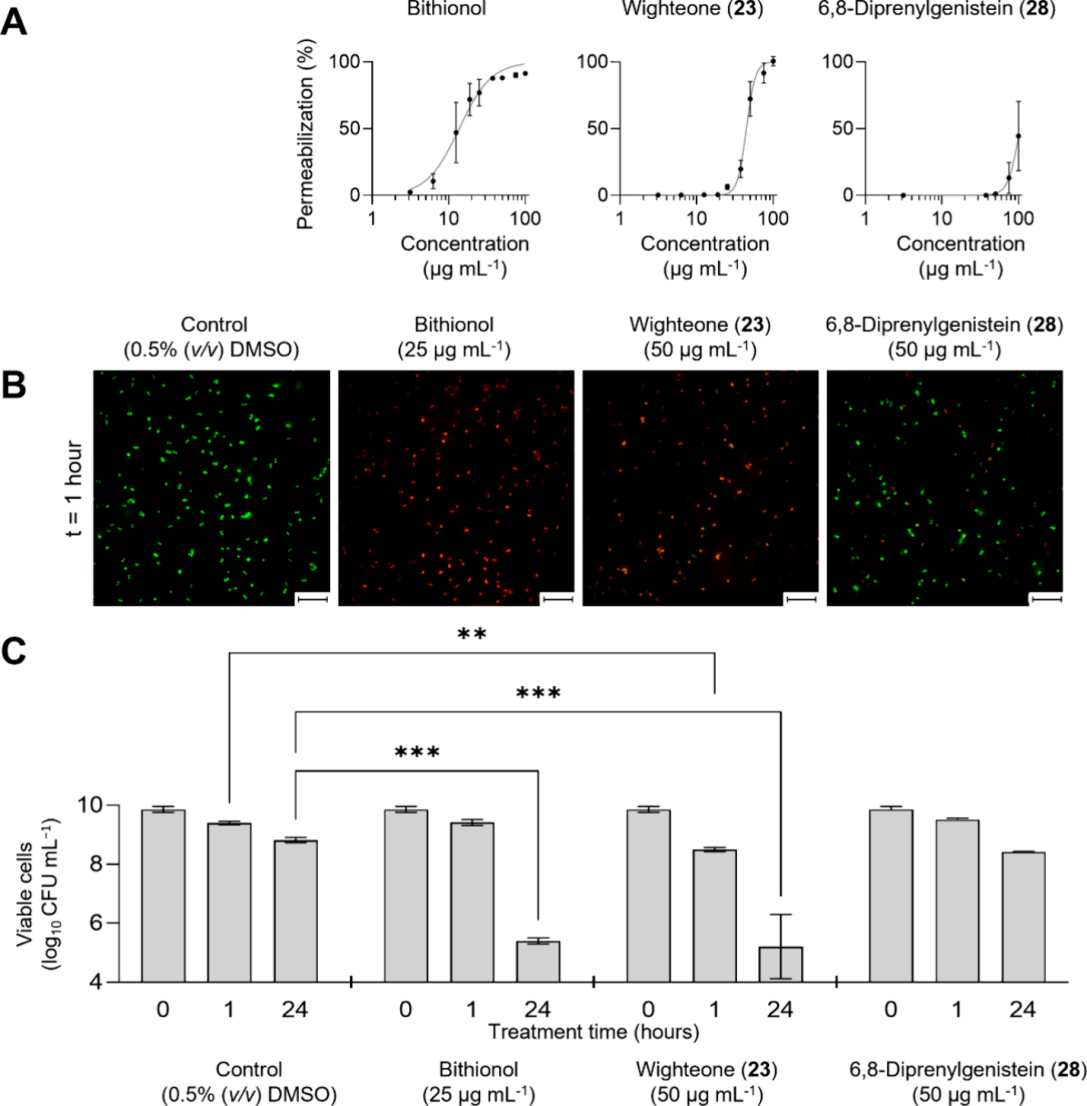
Membrane permeabilization and cell viability of MRSA after treatment with positive control bithionol and prenylated phenolics wighteone (**23**) and 6,8-diprenylgenistein (**28**). (A) Concentration–response relationships for membrane permeabilization based on propidium iodide (PI) uptake measured by fluorescence spectrometry. Data points represent the average membrane permeabilization (% compared to maximum fluorescence control, i.e., heated cells), with error bars showing the standard deviation of the mean from biological replicates (*n* ≥ 3). (B) Fluorescence microscopy images of MRSA stained with PI (red, permeabilized) and SYTO 9 (green, not permeabilized) after 1 h of treatment. Scale bar: 10 μm. Fluorescence microscopy images after 5 min of treatment are shown in Figure S58. (C) Viable cell counts after 0, 1, and 24 h of treatment. Bars show averages and error bars represent standard deviations of replicates (*n* = 3). Statistical differences were determined by two-way ANOVA. Asterisks indicate significant differences compared to the 0.5% (v/v) DMSO control at each time point (0, 1, and 24 h): (∗) *p* < 0.05, (∗∗) *p* < 0.01, and (∗∗∗) *p* < 0.001.

The results of fluorescence spectrometric measurements (Figure 2A) were consistent with fluorescence microscopy (Figure 2B). The positive control bithionol and prenylated phenolic wighteone (**23**) induced cell permeabilization (Figure 2B). The rapid permeabilization observed within 5 min of treatment with wighteone (**23**, Figure S58) suggests that its primary mechanism is targeting the membrane, rather than membrane permeabilization being a secondary effect of bacterial stress. Prenylated phenolics can induce leakage in model membranes,^24,25,36^ supporting the idea that membrane permeabilization is the result of a direct interaction of prenylated phenolics with the lipid bilayer. In contrast, the majority of the cells treated with 6,8-diprenylgenistein (**28**) were not permeabilized (Figure 2B and Figure S58), which is in line with previous research,^17^ suggesting that the antimicrobial activity of 6,8-diprenylgenistein (**28**) cannot be primarily attributed to membrane permeabilization.

Viable cell counts (Figure 2C) showed that treatment with bithionol or 6,8-diprenylgenistein (**28**) for 1 h did not decrease the number of viable cells compared to the control. Although no decrease in viable cells was observed for bithionol (Figure 2C), cells were permeabilized (Figure S58), suggesting that permeabilized cells are not necessarily dead, as described previously by Davey and Hexley.^21^ In contrast, treatment with wighteone (**23**) for 1 h did result in a significant reduction of viable cells. After 24 h, both bithionol and wighteone (**23**) showed a significant decrease of viable cells (>3 log reduction), while 6,8-diprenylgenistein (**28**) did not. The lack of significant bactericidal activity for 6,8-diprenylgenistein (**28**) was unexpected, given its lower MBC value compared to wighteone (**23**) (Table 1). This discrepancy could be due to differing experimental conditions (e.g., time and inoculum size) between membrane permeabilization (both spectrometric and microscopic) and antimicrobial activity (broth microdilution) assays.

#### Quantitative Assessment of Permeabilization Capacity of Prenylated Phenolics

As quantitative spectrometric measurements were in line with fluorescence microscopy, fluorescence spectrometry was used to quantitatively assess the ability of all other 34 prenylated phenolics in Figure 1 to permeabilize the cytoplasmic membrane of MRSA (concentration–response curves in Figure S57). From all concentration–response curves of individual biological replicates, EC_10_ values were extracted, i.e., the concentration at which 10% of the maximal response is achieved. This threshold was chosen for quantifying permeabilization because it allows for a more sensitive detection of permeabilization, resulting in more prenylated phenolics for which an EC_10_ could be determined compared to higher thresholds (e.g., EC_50_). Of all 36 prenylated phenolics, 15 compounds (**1**, **2**, **3**, **5**, **7**, **13**, **14**, **16**, **20**, **21**, **24**, **25**, **34**, **35**, and **36**) showed a flat concentration–response curve, which did not achieve 10% of the maximal response (see Figure S57). This shows that they did not permeabilize the cytoplasmic membrane of MRSA at the highest tested concentration (100 μg mL^–1^), and were thus regarded as nonpermeabilizers. A lack of effective permeabilization can have two potential explanations: (i) the prenylated phenolic does not interact with the cytoplasmic membrane at all, or (ii) the prenylated phenolic interacts with the cytoplasmic membrane without leading to permeabilization. The EC_10_ of permeabilization for the 21 permeabilizing prenylated phenolics and the known permeabilizer bithionol are shown in Figure 3.

**Figure 3:**
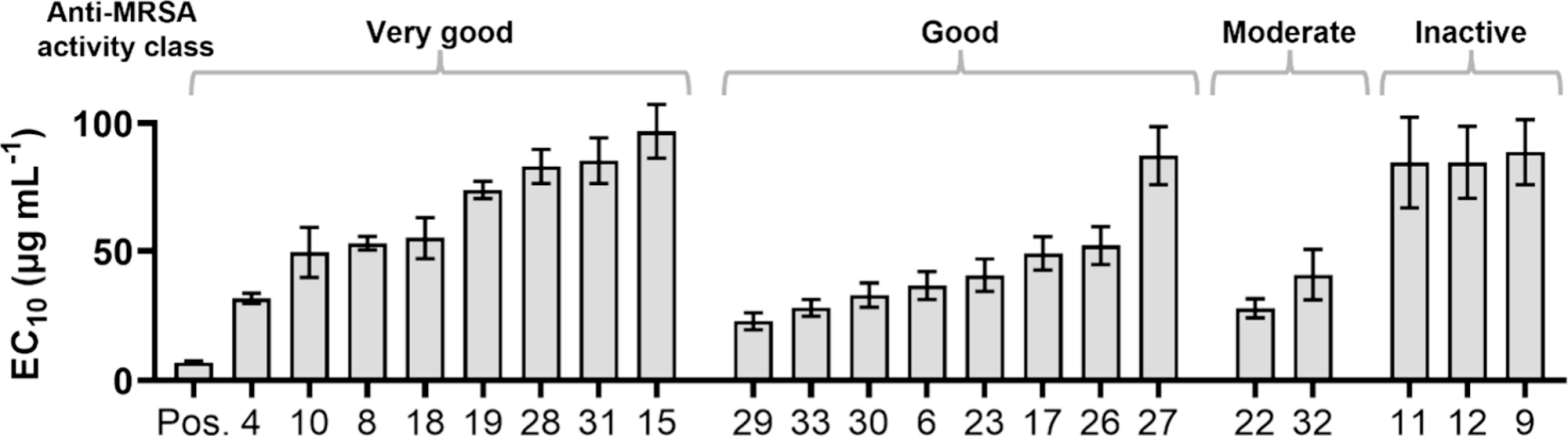
EC_10_ of permeabilization for prenylated phenolics and positive control bithionol (Pos.). The EC_10_ values were extracted from concentration–response curves (Figure S57) of individual replicates of PI uptake measured by fluorescence spectrometry. Prenylated phenolics with an EC_10_ > 100 μg mL^–1^ are not shown. Error bars represent standard deviation of the mean of biological replicates (*n* ≥ 3). The classification of anti-MRSA activity is shown on top of the graphs. Compounds with MIC ≤ 15 μg mL^–1^ were classified as very good, 15 < MIC ≤ 25 μg mL^–1^ as good, 25 < MIC ≤ 50 μg mL^–1^ as moderate, and MIC > 50 μg mL^–1^ as inactive.

Figure 3 shows that the lowest EC_10_ was found for positive control bithionol (EC_10_ of 7 ± 1 μg mL^–1^). Furthermore, Figure 3 illustrates that, for compounds with antimicrobial activity (MIC ≤ 50 μg mL^–1^), permeabilization is not correlated to their antimicrobial potency. Compounds **15**, **19**, **28**, and **31** possessed potent anti-MRSA activity (MIC ≤ 15 μg mL^–1^), but did possess effective permeabilization of MRSA (EC_10_ ≥ 50 mL^–1^). The most effective permeabilization was observed for luteone (**29**) and neobavaisoflavone (**22**), which were not the most potent antimicrobials (MIC 25 and 38 μg mL^–1^, respectively).

To conclude, for prenylated phenolics with antimicrobial activity (MIC ≤ 50 μg mL^–1^), permeabilization was not correlated to their antimicrobial potency (see also Figure S59). This suggests that permeabilization of the cytoplasmic membrane is not the sole mechanism by which prenylated phenolics exert their inhibitory effects on MRSA. Additionally, our results suggest that molecular properties linked to effective membrane permeabilization will differ from molecular properties linked to anti-MRSA activity.

### SARs for Permeabilization

#### Matched Molecular Pairs (MMPs) to Gain Insights in SARs for Permeabilization

To gain insights in the molecular properties linked to effective permeabilization, SARs for permeabilization were defined. These SARs can help to identify the molecular features of prenylated phenolics driving the interaction with and the effective permeabilization of the cytoplasmic membrane. To define SARs, MMPs obtained from MOEsaic were studied. MMPs are sets of prenylated phenolics differing in one structural feature. Especially interesting are the MMPs which show a distinct permeabilization capacity, as they highlight structural features influencing membrane permeabilization. Figure 4 shows the key MMPs and their varying structural features. The varying structural features all relate to the number of prenyl groups, configuration of prenyl group, and number of hydroxyl groups, and will be explained in the following sections. No MMPs differing in phenolic subclass were included.

**Figure 4:**
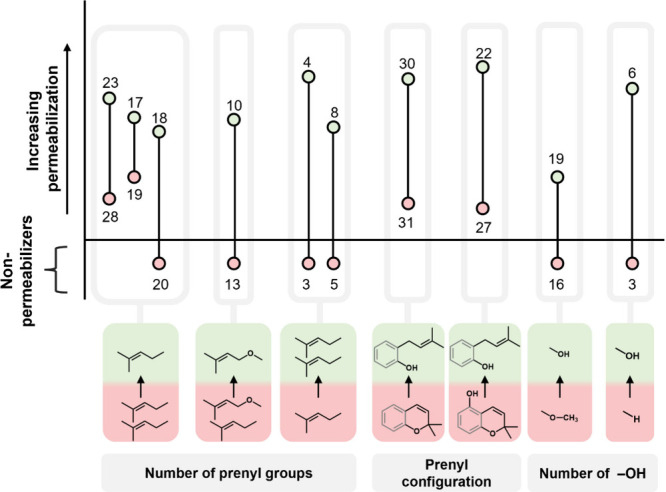
Permeabilization SARs for prenylated phenolics illustrated by MMPs obtained from MOEsaic. Data points are labeled with the numbers of the corresponding compounds (see Figure 1 for structures). MMPs are organized based on their variant structural feature, which is shown below the graph. Permeabilization on the *Y* axis is defined by the average EC_10_ (μg mL^–1^), nonpermeabilizing prenylated phenolics are shown below the *X* axis. Values on the *Y* axis and error bars were omitted for the sake of simplicity. The green color and red color indicate the compound of the MMP with highest and lowest permeabilization capacity, respectively.

Figure 4 shows that several diprenylated phenolics (**28**, **19**, **20**, and **13**) were poorer permeabilizers than their monoprenylated counterparts (**23**, **17**, **18**, and **10**). These results are in line with previous research by Araya-Cloutier et al.,^17^ which demonstrated that the permeabilization of the cytoplasmic membrane of *L. monocytogenes* by prenylated phenolics decreases as their relative hydrophobic surface area increases. For two MMPs (**3**/**4** and **5**/**8**), however, the opposite was observed and diprenylation resulted in more effective permeabilization.

Figure 4 also includes two MMPs which illustrate how the configuration of the prenyl group affects permeabilization. Chain prenylated licoisoflavone A (**30**) and neobavaisoflavone (**22**) show more effective permeabilization compared to their ring prenylated counterparts licoisoflavone B (**31**) and glabrone (**27**). These results highlight that cyclization of the prenyl group with a neighboring hydroxyl decreases permeabilization. In previous work, molecular dynamics simulations also showed a more favorable intercalation inside the lipid bilayer for chain prenylated wighteone (**23**) compared to ring prenylated glabridin (**18**).^25^ Ring closure reduces the flexibility of the hydrophobic prenyl group. The flexibility of a chain prenyl group potentially allows for a more effective interaction with the cytoplasmic membrane. In addition, cyclization of the prenyl group with a neighboring hydroxyl also results in the loss of a free hydroxyl. The importance of free hydroxyl groups for effective permeabilization (MMP **16**/**19** and **3**/**6**) is also highlighted in Figure 4. Possibly, hydroxyl groups interact with the phospholipid headgroups of the cytoplasmic membrane. Previous molecular dynamics simulations showed that hydrophilic regions of prenylated phenolics interacted with polar headgroups.^25^ This may explain the potent permeabilization of diprenylated phenolic 6′-prenylpiscidone (**33**; see Figure 3), which, in addition to two prenyl groups, possesses four hydroxyl groups (see Figure 1). These characteristics likely enhance its interaction with the cytoplasmic membrane.

To conclude, the MMPs in Figure 4 showed that (i) diprenylation was not always linked to lower permeabilization, (ii) hydroxylation improved permeabilization, and (iii) chain prenylated phenolics permeabilized more effectively than ring prenylated phenolics.

#### Molecular Descriptors to Gain Insight into SARs for Permeabilization

Figure 4 highlights the importance of both hydrophobicity (i.e., prenyl groups) and hydrophilicity (i.e., hydroxylation). Moreover, previous research emphasized the importance of relative hydrophobicity.^17^ Therefore, Figure 5A and B show the correlation between permeabilization (EC_10_) and the hydrophobic-to-polar surface area ratio (*ASA_H*/*ASA_P*). The ratio of molecular descriptors *ASA_H*/*ASA_P* specifically compares the balance between hydrophobic and polar surface areas rather than indicating the total proportion of the molecule that is hydrophobic. Figure 5A and B include prenylated phenolics with antimicrobial activity against MRSA (i.e., MIC ≤ 50 μg mL^–1^).

**Figure 5:**
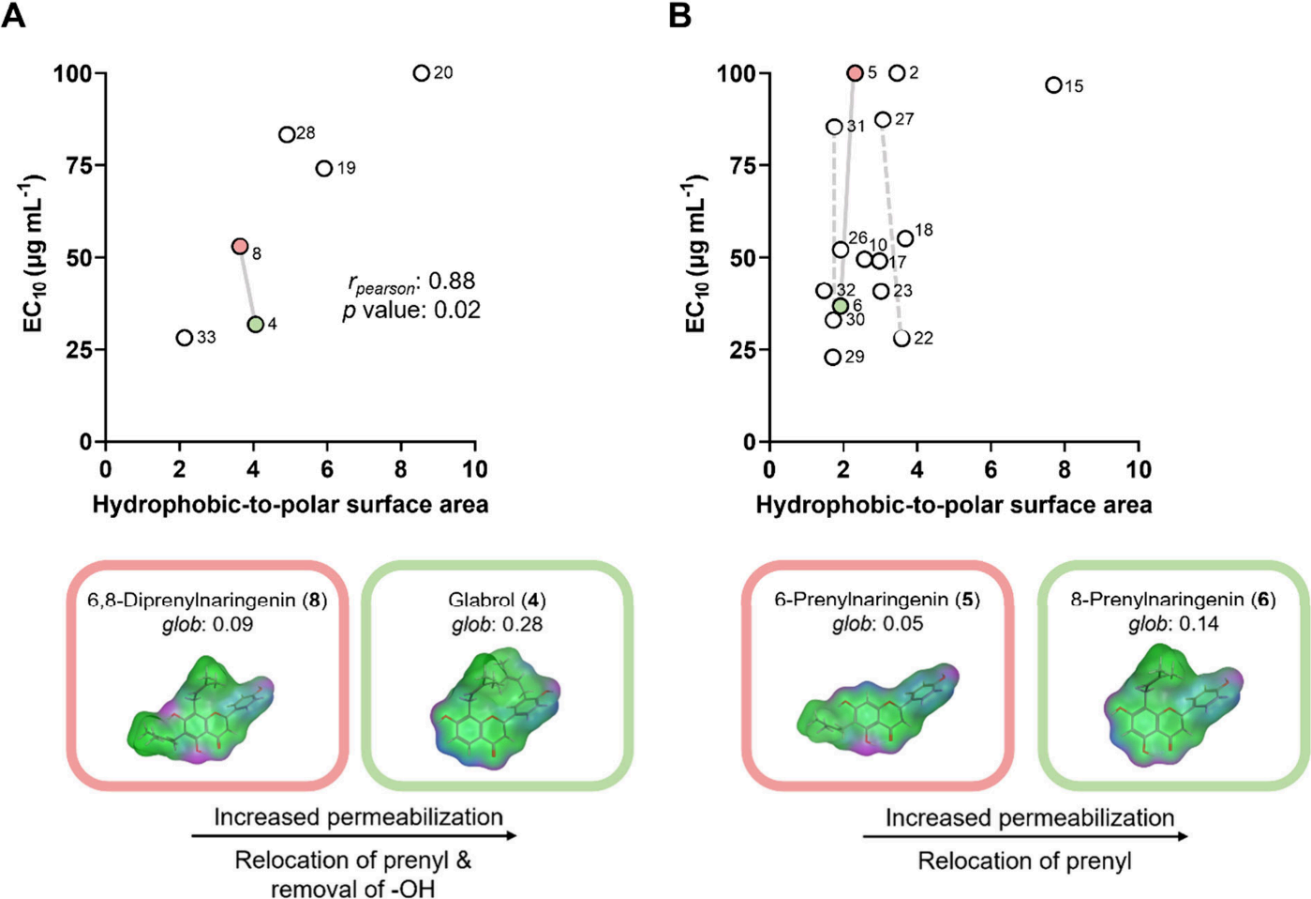
Correlation plots between permeabilization (EC_10_ in μg mL^–1^) and the hydrophobic-to-polar surface area ratio (*ASA_H*/*ASA_P*, obtained from MOE) of (A) diprenylated and (B) monoprenylated phenolics with antimicrobial activity against MRSA (MIC ≤ 50 μg mL^–1^). Nonpermeabilizing prenylated phenolics (**2**, **5**, and **20**) were included with an EC_10_ of 100 μg mL^–1^. The dotted gray lines in the correlation plot (B) indicate the MMPs with varying prenyl configuration (ring vs chain; Figure 4). Next to the correlation plots, a pair of prenylated phenolics with similar hydrophobic-to-polar surface area ratios and differing permeabilization efficacy is exemplified (indicated in correlation plots with solid gray lines), showing their molecular structures, surface maps, and globularity (*glob*) values. Surface maps show mildly polar regions in blue, hydrogen-bond donors and/or acceptors in pink, and hydrophobic regions in green. Compound numbers refer to Figure 1.

For diprenylated phenolics (Figure 5A), a strong positive correlation between permeabilization (EC_10_) and the hydrophobic-to-polar surface area ratio was observed (*r*_pearson_ = 0.88), indicating that compounds with a higher hydrophobic-to-polar surface area ratio are less potent permeabilizers. This is in line with previous research, where a significant negative correlation between the relative hydrophobic molecular surface area of a smaller set of prenylated phenolics and permeabilization was found.^17^ These findings emphasize the importance of a small hydrophobic-to-polar surface area ratio for effective permeabilization of diprenylated compounds, whereas antimicrobial activity against MRSA was previously associated with a large hydrophobic volume and balanced hydrophilic surface areas.^9^ This suggests that the mechanism of antimicrobial action of diprenylated phenolics with a large hydrophobic-to-polar surface area [e.g., 6,8-diprenylgenistein (**28**)] cannot be completely attributed to membrane permeabilization. To better understand the antimicrobial mechanism of action of diprenylated phenolics, investigating other membrane effects (e.g., rigidification and depolarization) and further studying membrane permeabilization using model membrane systems in combination with molecular dynamics simulations will help validate the membrane effects of these compounds.

In contrast, for monoprenylated phenolics (Figure 5B) no correlation between permeabilization and hydrophobic-to-polar surface area ratio was observed. Four compounds (**2**, **5**, **27**, and **31**) showed higher EC_10_ values (i.e., poorer permeabilization) compared to a larger set of monoprenylated phenolics with similar hydrophobic-to-polar surface area ratios. This suggests that other molecular properties are important for effective permeabilization.

Previous research using model membranes (liposomes) demonstrated that, in addition to hydrophobicity, molecular shape influenced the permeabilization of the model membrane by prenylated phenolics.^24,36^ Therefore, we investigated the molecular shape of molecular pairs with a similar hydrophobic-to-polar surface area ratio but differing permeabilization efficacy. The selected molecular pairs and their globularity (*glob*) are highlighted with gray solid lines in the correlation plots in Figure 5A and B, and their molecular structures are shown next to the correlation plots.

The molecular pairs in Figure 5 highlight small structural changes that influence permeabilization efficacy without affecting the hydrophobic-to-polar surface area ratio. These structural changes alter the 3D configuration and overall shape of the compound, as evidenced by the molecular surface maps. For diprenylated phenolics, the more globular glabrol (**4**) was found to permeabilize more effectively than 6,8-diprenylnaringenin (**8**) (Figure 5A). Similarly, 8-prenylnaringenin (**6**) permeabilized more effectively than 6-prenylnaringenin (**5**) (Figure 5B). A similar trend was observed for bavachinin (**2**; *glob* of 0.05), which also showed poorer permeabilization compared to monoprenylated phenolics with a similar hydrophobic-to-polar surface area ratio.

In summary, a strong negative correlation between permeabilization and the hydrophobic-to-polar surface area ratio was observed for diprenylated phenolics. For monoprenylated phenolics, our findings suggest that prenyl configuration and molecular shape play a role in permeabilization. Previous research using model membranes (liposomes) demonstrated that molecular shape, in addition to hydrophobicity, influenced the localization of three prenylated phenolics within the membrane.^24^ In particular, more hydrophobic and less globular prenylated phenolics interacted more deeply in the membrane.^24^ Possibly, prenylated phenolics need to interact near the phospholipid headgroups in order to effectively permeabilize the bacterial membrane, explaining the poorer permeabilization of prenylated phenolics with a high hydrophobic-to-polar surface area ratio or low globularity. Potentially, this deeper interaction in the membrane does not result in permeabilization, instead these prenylated phenolics might inhibit bacterial growth by affecting the fluidity, elasticity, potential, or conductivity of the membrane.^20,37,38^

While the permeabilization of bacterial membranes may contribute to antimicrobial activity, it also raises concerns regarding the potential use of prenylated phenolics as alternatives to traditional antimicrobials. Numerous studies have reported cytotoxic effects of prenylated phenolics in mammalian cell lines,^41,42^ and prenylation has been associated with increased cytotoxicity.^43–45^ Reported EC_50_ values for cytotoxicity vary considerably across studies (e.g., from 5.7 to >50 μM for wighteone (**23**)^46,47^ ), likely due to differences in assay conditions and cell models.^48–50^ Prenylated phenolics are absorbed in the small intestine, but their intestinal permeability and systemic exposure remain poorly characterized.^51^ Therefore, improved understanding of the pharmacokinetic and pharmacodynamic properties of prenylated phenolics is needed to relate *in vitro* toxicity to physiologically relevant concentrations and to evaluate their safety.

## Conclusion

This study investigated the inhibition and permeabilization of MRSA by 36 C- and O-prenylated phenolics. The only O-prenylated phenolic that demonstrated antimicrobial activity, 7-*O*-prenylnaringenin (**10**), was more active than its C-prenylated analogues. Fluorescence microscopy showed that permeabilization occurred within 5 min for wighteone (**23**), suggesting a direct interaction with the membrane. For diprenylated phenolics, effective permeabilization was linked to a small hydrophobic-to-polar surface area ratio. For monoprenylated phenolics, prenyl configuration (chain) and molecular shape (globular) were important for effective permeabilization. These structural features might facilitate an interaction close to the phospholipid headgroups, possibly resulting in permeabilization of the cytoplasmic membrane. Overall, this study provides a quantitative overview of membrane permeabilization by prenylated phenolics, contributing to our understanding of the antimicrobial activity of prenylated phenolics. Future studies using model membranes and molecular dynamics simulations could further elucidate the role of hydrophobic-to-polar surface area, prenyl configuration, and molecular shape in the membrane interactions of prenylated phenolics.

## Experimental Section

### General Experimental Procedures

Identities and purities of all prenylated phenolics were confirmed by reversed-phase ultrahigh performance liquid chromatography coupled to photodiode array and electrospray ionization ion trap mass spectrometry detection (RP-UHPLC-PDA-ESI-IT-MS^*n*^; see Supporting Information A3 and Table S1). Purity of synthesized and purified prenylated phenolics was additionally determined by proton nuclear magnetic resonance spectroscopy (^1^H NMR; see Supporting Information A4 and Table S1). Fluorescence measurements were performed using a Spectramax ID3 plate reader (Molecular Devices, Sunnyvale, CA, U.S.A.), and microscopy images were acquired using an Axioskop fluorescence microscope (Carl Zeiss, Oberkochen, Germany) equipped with a XC30 camera (Olympus, Hamburg, Germany). Statistical analysis and curve fitting were performed using GraphPad Prism version 9.3.1 (GraphPad Software, San Diego, CA, U.S.A.). Statistical significance was determined using the following levels: (∗) *p* < 0.05, (∗∗) *p* < 0.01, and (∗∗∗) *p* < 0.001.

### Materials

#### Collection of Prenylated Phenolics

In addition to commercially available compounds, our collection of prenylated phenolics consisted of synthesized and purified prenylated phenolics. Bavachinin (**1**), bavachin (**2**), isobavachin (**3**), 6-prenylnaringenin (**5**), isoxanthohumol (**9**), glyasperin C (**17**), neobavaisoflavone (**22**), wighteone (**23**), α-isowighteone (**24**), 6,8-diprenylgenistein (**28**), licoisoflavone A (**30**), and glycyrrhisoflavone (**32**) were purchased from Chemfaces (Wuhan, China). Glabridin (**18**) was purchased from Wako (Osaka, Japan). Lupiwighteone (**25**), isowighteone (**26**), and luteone (**29**) were purchased from Plantech UK (Reading, U.K.). 6′-Prenylpiscidone (**33**) was purchased from Carbosynth, Ltd. (Berkshire, U.K.). 8-Prenylnaringenin (**6**), 3′-prenylnaringenin (**7**), 6,8-diprenylnaringenin (**8**), 7-*O*-prenylnaringenin (**10**), 6-*C*,7-*O*-diprenylnaringenin (**11**), 8-*C*,7-*O*-diprenylnaringenin (**12**), 3′-*C*,7-*O*-diprenylnaringenin (**13**), 7,4′-*O*-diprenylnaringenin (**14**), 7-*O*-prenylgenistein (**34**), 6-*C*,7-*O*-diprenylgenistein (**35**), and 8-*C*,7-*O*-diprenylgenistein (**36**) were synthesized in this study (see Supporting Information A2). Glabrol (**4**), 4′-*O*-methylglabridin (**15**), licorisoflavan A (**16**), licoricidin (**19**), hispaglabridin A (**20**), hispaglabridin B (**21**), glabrone (**27**), and licoisoflavone B (**31**) were previously purified from *Glycyrrhiza* spp. roots.^52,53^

#### Other Materials

Methanol (MeOH) and glacial acetic acid were purchased from Biosolve (Valkenswaard, Netherlands). Water for other purposes than UHPLC was prepared using a Milli-Q water purification system (Merck Millipore, Billerica, MA, U.S.A.). Dimethyl sulfoxide (DMSO) was purchased from Merck Millipore. Propidium iodide (PI), bithionol, and *tert*-butanol ≥ 98% (w/w) were purchased from Sigma-Aldrich (St Louis, MO, U.S.A.). LIVE/DEAD BacLight Bacterial Viability Kit containing PI and SYTO 9 was purchased from ThermoFisher (Eugene, OR, U.S.A.). Vancomycin hydrochloride (called vancomycin here) was purchased from PanReac Applichem (Darmstadt, Germany). Tryptone soya broth (TSB) and bacteriological agar were obtained from Oxoid, Ltd. (Basingstoke, U.K.), peptone physiological salt solution (PPS) from Tritium Microbiologie (Eindhoven, Netherlands). Tryptone soy agar (TSA) plates were prepared using TSB and bacteriological agar.

### Antimicrobial Activity Determination

Prenylated phenolics were tested for their antimicrobial activity against MRSA 18HN (strain provided by RIVM, Bilthoven, Netherlands). Bacteria were streaked onto a TSA plate from a −80 °C glycerol stock and incubated 24 h at 37 °C. One colony was transferred to 10 mL TSB and further incubated for 18 h at 37 °C. This overnight culture was diluted 4,000 × which resulted in an initial inoculum size of 4.7 ± 0.2 log_10_ CFU mL^–1^ in the final assay. Stock solutions of the prenylated phenolics were prepared in DMSO and subsequently diluted with TSB. Prenylated phenolics were tested at concentrations ranging from 3.1 to 50 μg mL^–1^. The maximum DMSO concentration in the final assay was 1% (v/v), which did not affect bacterial growth (Figure S56A). Vancomycin stock was prepared at 10 mg mL^–1^ in water +0.1% (v/v) acetic acid and filter-sterilized prior to further dilution using TSB. Vancomycin was tested at 2 μg mL^–1^ as positive control. The antimicrobial activity of bithionol, the positive control used in cell membrane permeability assays, was assessed at concentrations ranging from 1.56 to 25 μg mL^–1^. Furthermore, negative controls [TSB and 1% (v/v) DMSO in TSB with bacteria] and blanks (prenylated phenolics at 3.1 μg mL^–1^ without bacteria) were included for optical comparison and sterility control.

Equal volumes (100 μL) of inoculum and prenylated phenolics in TSB were added to a clear nonbinding 96-well plate (Greiner Bio One, Kremsmünster, Austria). The 96-well plate was incubated for 24 h at 37 °C in a Tecan Infinite 200Pro (Tecan Group, Ltd., Zürich, Switzerland). Before each measurement, linear shaking was applied for 5 s with an amplitude of 3 mm. Optical density (OD) was measured at 600 nm every 10 min. Inhibition of growth was assessed by measuring the time-to-detection (TTD), i.e., the time to reach a change in OD_600_ of 0.05 units.^17^ The growth delay (GD) was calculated by subtracting the TTD of the DMSO blank from the TTD of the compound. When no change in OD_600_ (ΔOD_600_ < 0.05) was observed after 24 h of incubation, cell viability was verified by performing viable cell counts. In short, duplicates of wells showing no change in OD_600_ were combined and 100 μL was decimally diluted in PPS and spread onto TSA plates. Plates were incubated for 24 h at 37 °C, after which colonies were counted. The MIC was defined as the lowest compound concentration that inhibits growth compared to the initial inoculum (with maximum increase in cell count of 0.5 log_10_ CFU mL^–1^) and MBC as the lowest concentration resulting in at least 3 log_10_ CFU mL^–1^ reduction of the initial inoculum.^54^ Prenylated phenolics were tested in three independent biological replicates, each performed in duplicate. All experiments were conducted assuming that nominal concentrations of prenylated phenolics corresponded similarly to their effective concentrations in the experimental system.^50^

Natural compounds are generally classified as compounds with very good antimicrobial activity when their MIC is ≤ 15 μg mL^–1^.^9,17^ Compounds with 15 < MIC ≤ 25 μg mL^–1^ are classified as compounds with good activity, 25 < MIC ≤ 100 μg mL^–1^ as moderately active, and MIC > 100 μg mL^–1^ as inactive.^9,17^ In this research the highest tested concentration was 50 μg mL^–1^. Therefore, we classified compounds with MIC ≤ 15 μg mL^–1^ as very good activity, 15 < MIC ≤ 25 μg mL^–1^ as good activity, 25 < MIC ≤ 50 μg mL^–1^ as moderate activity, and MIC > 50 μg mL^–1^ as inactive at 50 μg mL^–1^. If a range was obtained for the MIC, the upper value was considered for classification purposes.

### Cell Membrane Permeability

The fluorescent probe propidium iodide (PI) was used to investigate the permeability of the cytoplasmic membrane upon exposure to prenylated phenolics. MRSA was streaked from a −80 °C glycerol stock to a TSA plate and incubated for 24 h at 37 °C. Subsequently, one colony was transferred to 50 mL TSB and incubated for 5 h at 37 °C while being continuously shaken at 150 rpm. Cells were harvested by centrifugation (4696*g*, 4 °C, 10 min) and washed twice with PPS (pH 7.2). The cell pellet, obtained after the final washing step, was suspended in 4 mL PPS. The final initial inoculum in the assay was 9.9 ± 0.1 log_10_ CFU mL^–1^.

Stock solutions of PI and prenylated phenolics were diluted in PPS to concentrations of 60 μM and 12.5–400 μg mL^–1^, respectively. Next, 50 μL of each solution and 100 μL of inoculum were added to a black nonbinding 96-well plate with clear bottom (Greiner Bio One, Kremsmünster, Austria). The final concentrations of prenylated phenolics tested ranged between 3.1 to 100 μg mL^–1^. In addition to prenylated phenolics, bithionol was included as it is known to induce membrane permeabilization.^33–35^ The maximum DMSO concentration in the final assay was 2% (v/v), which did not affect PI uptake (Figure S56B). Background fluorescence was accounted for using controls and blanks (cells with PI, without compounds; cells with PI and 2% (v/v) DMSO, without compounds; PI alone; compounds with PI at lowest and highest concentration tested, without cells). Cells heated at 95 °C for 10 min in a Thermomixer (Eppendorf, Hamburg, Germany) were measured to establish maximum fluorescence.

Plates were incubated for 1 h at 37 °C in a Spectramax ID3 plate reader. During incubation, each plate was excited at 520 nm and fluorescence was measured every minute at 620 nm (top read mode). Before each measurement, shaking was applied for 5 s (orbital mode, medium intensity). All compounds were tested in at least two biological replicates, and compounds that resulted in PI uptake were tested in at least five biological replicates. All experiments were conducted assuming that nominal concentrations of prenylated phenolics equaled their effective concentrations in the experimental system.^50^

To account for background fluorescence of PI and free DNA, the fluorescence of the negative control was subtracted. The percentage of permeabilization was calculated according to the following equation:

$$\mathrm{permeabilization}\left(\%\right)=\frac{\mathrm{compound}\left({\mathrm{RFU}}_{\mathrm{av}}\right)-\mathrm{negative control}\left({\mathrm{RFU}}_{\mathrm{av}}\right)}{\mathrm{heated cells}\left({\mathrm{RFU}}_{\mathrm{av}}\right)-\mathrm{negative control}\left({\mathrm{RFU}}_{\mathrm{av}}\right)}\times 100\%$$

where RFU_av_ is the average fluorescence of the last 10 min of the measurement. Concentration–response curves were established by fitting the data using nonlinear regression (Sigmoidal, 4PL, X is concentration) with GraphPad Prism. Upon fitting the data, the average fluorescence of the negative (RFU) and average fluorescence of the heated cells (RFU) were constrained to 0 and 100%, respectively. EC_10_ values were determined from concentration–response curves of individual biological replicates and then averaged.

### Fluorescence Microscopy and Viable Cell Counting

Fluorescence microscopy was performed to visualize and qualitatively assess permeabilization. Cells were prepared as described in section 3.4. The cell pellet, obtained after the final washing step, was suspended in 4 mL PPS. The final initial inoculum in the assay was 9.7 ± 0.1 log_10_ CFU mL^–1^. Stock solutions of wighteone (**23**) and 6,8-diprenylgenistein (**28**) were diluted in PPS to a concentration of 100 μg mL^–1^. Bithionol was diluted to a concentration of 50 μg mL^–1^. These concentrations were chosen based on the results of the propidium iodide assay as described in section 3.4. Furthermore, a solution of 1.0% (v/v) DMSO in PPS was prepared as control. Equal volumes of inoculum and compound were combined. After 5 and 60 min of treatment, 45 μL of suspension was taken for fluorescence microscopy. Cells were stained with SYTO 9 and PI at final concentrations of 10 and 60 μM, respectively, following the manufacturer’s instructions. Stained cells were visualized using a Axioskop fluorescence microscope equipped with a XC30 camera. At least five images were taken for each treatment and time point. Two independent biological replicates were performed.

Fluorescence microscopy was combined with viable cell counting to get insight into the number of viable cells at the conditions used during assessment of permeabilization. The cell suspension prepared for fluorescence microscopy was incubated at 37 °C in a thermomixer. Viable cell counts were performed by taking 100 μL of cell suspension after 0, 1, and 24 h of treatment. Cell suspension was decimally diluted in PPS and spread onto TSA plates. Plates were incubated for 24 h at 37 °C after which colonies were counted. A two-way ANOVA was conducted to compare the effects of treatments [bithionol, wighteone (**23**), and 6,8-diprenylgenistein (**28**)] at different time points (0, 1, and 24 h) with the control [0.5% (v/v) DMSO].

### *In Silico* Calculation and Analysis of Molecular Descriptors

Chemical structures of prenylated phenolics were loaded into the modeling software Molecular Operating Environment (MOE, version 2022.02, Chemical Computing Group, Montreal, QC, Canada). The force field in MOE was set to MMFF94x. A conformational search (LowModeMD, RMS gradient 0.1 kcal mol^–1^ Å^–1^, other settings default) was performed. The lowest energy conformer was further refined using MOPAC force field (RMS gradient 0.01 kcal mol^–1^ Å^–1^). Optimized chemical structures were used to calculate 2D and 3D molecular descriptors in MOE and imported into MOEsaic along with permeabilization values (EC_10_) for generating MMPs and visualizing SARs for permeabilization.

For antimicrobial prenylated phenolics (MIC ≤ 50 μg mL^–1^, *n* = 21), Pearson correlation coefficients (*r*) between molecular descriptors and permeabilization (EC_10_) were calculated in RStudio (version 2023.06.1, R Foundation for Statistical Computing, Vienna, Austria). The percentage of molecular species in undissociated form, log *D*, and aqueous solubility were calculated using MarvinSketch version 23.14.0 (ChemAxon, https://www.chemaxon.com).

## Supplementary Material



## Data Availability

The NMR data are made available publicly under the CC 4.0 BY-SA license. DOI: 10.5281/zenodo.15727855.

## Notes

(A) Experimental section and results of chemical synthesis, RP-UHPLC-PDA-ESI-IT-MS^*n*^ analysis, and NMR spectroscopy and (B) antimicrobial activity (controls and growth delay), permeabilization activity (controls, concentration–response curves, and fluorescence microscopy images), and calculated properties of prenylated phenolics (PDF)
