# Supplementary material for: Effective Membrane Permeabilization of Methicillin-Resistant Staphylococcus aureus by Prenylated Phenolics
Source: J Nat Prod. 2025 Aug 19;88(9):2065–75. doi: 10.1021/acs.jnatprod.5c00540 (PMC12481577; doi:10.1021/acs.jnatprod.5c00540)
Supplement: Supplementary file 1 [file np5c00540_si_001.pdf]

Supporting information

Effective membrane permeabilization of MRSA by prenylated phenolics

Janniek H. Ritsema[1,2], Nynke I. Kramer[2], Wouter J.C. de Bruijn[1], Sarah van Dinteren[1], Maurice C.R. Franssen[3], Jean-Paul Vincken[1], Carla Araya-Cloutier[1*]

[1] Laboratory of Food Chemistry, Wageningen University, Bornse Weilanden 9, 6708 WG Wageningen, The Netherlands

[2] Toxicology Chair Group, Wageningen University, Stippeneng 4, 6708 WE Wageningen, The Netherlands

[3] Laboratory of Organic Chemistry, Wageningen University, Stippeneng 4, 6708 WE Wageningen, The Netherlands

*Corresponding author (E-mail: carla.arayacloutier@wur.nl)

Table of Contents

Supporting information A **4**

Supporting information A1. Materials **4**

Supporting information A2. Chemical synthesis **4**

Supporting information A3. RP-UHPLC-PDA ESI-IT-MS^n analysis **6**

Supporting information A4. NMR spectroscopy **8**

Table S1. RP-UHPLC-PDA-IT-MSn analysis of prenylated phenolics (**1 – 36)** 9

Table S2. ^{1}H and ^{13}C NMR spectral data for synthesized prenylated phenolics in acetone-*d6* 12

Figure S1-5. NMR spectra of 8-prenylnaringenin (**6**) in acetone-*d6* 15

Figure S6-10. NMR spectra of 3'-prenylnaringenin (**7**) in acetone-*d6* 17

Figure S11-15. NMR spectra of 6,8-diprenylnaringenin (**8**) in acetone-*d6* 20

Figure S16-20. NMR spectra of 7-*O*-prenylnaringenin (**10**) in acetone-*d6* 22

Figure S21-25. NMR spectra of 6-*C*,7-*O*-diprenylnaringenin (**11**) in acetone-*d6* 25

Figure S26-30. NMR spectra of 8-*C*,7-*O*-diprenylnaringenin (**12**) in acetone-*d6* 27

Figure S31-35. NMR spectra of 3'-*C*,7-*O*-diprenylnaringenin (**13**) in acetone-*d6* 30

Figure S36-40. NMR spectra of 7,4-*O*-diprenylnaringenin (**14**) in acetone-*d6* 32

Figure S41-45. NMR spectra of 7-*O*-prenylgenistein (**34**) in acetone-*d6* 35

Figure S46-50. NMR spectra of 6-*C*,7-*O*-diprenylgenistein (**35**) in acetone-*d6* 37

Figure S51-55. NMR spectra of 8-*C*,7-*O*-diprenylgenistein (**36**) in acetone-*d6* 40

Supporting information B **43**

Figure S56. Signal of the controls during antimicrobial activity and permeabilization testing 43

Table S3. Growth delay (GD) of MRSA 18HN by prenylated phenolics inactive at 50 μg mL^{-1} 43

Figure S57. Dose-response curves for permeabilization by bithionol and phenolics 44

Figure S58. Fluorescence microscopy images of MRSA after treatment with bithionol and prenylated phenolics wighteone (**23**) and 6,8-diprenylgenistein (**28**)... 47

Figure S59. Correlation between permeabilization (EC_{10}) and antimicrobial activity (MIC) 48

Figure S60. Calculated properties of *O*-prenylated and *C*-prenylated phenolics 48

Table S4. Calculated neutral fraction, hydrophobicity, and solubility of prenylated phenolics. 49

References.. **50**

Supporting information A

Supporting Information A1. Materials

Naringenin ≥ 98% (*w/w*) and genistein ≥ 98% (*w/w*) were purchased from Cayman Chemical (Ann Arbor, MI, USA). Acetone-d_6, 3,3-dimethylallyl bromide, and anhydrous K_2CO_3 were purchased from Sigma-Aldrich (St Louis, MO, USA). ULC-MS grade water acidified with 0.1% (*v/v*) formic acid and acetonitrile acidified with 0.1% (*v/v*) formic acid were purchased from Biosolve (Valkenswaard, The Netherlands). HPLC grade acetone and acetonitrile were also purchased from Biosolve.

Supporting Information A2. Chemical synthesis

Prenylation of naringenin and genistein

To 17 mL of HPLC grade acetone in a 20 mL glass vial was added 1 equivalent (200 mg, 0.74 mmol) of naringenin or genistein and 2 equivalents of K_2CO_3 (approximately 205 mg, 1.47 mmol). The reaction was started by adding 1.7 equivalents of 3,3-dimethylallyl bromide (approximately 187 mg, 1.25 mmol). Subsequently, the vial's headspace was flushed with nitrogen gas and the vial was closed, after which the mixture was reacted at room temperature for 16 h under magnetic stirring. Then the reaction mixture was filtered (Whatman 595 ½ cellulose) and the solvent was removed under reduced pressure. This yielded a cream-white powdery solid for the genistein reaction mixture and an orange-yellow oil for the naringenin reaction mixture.

Purification of prenylated naringenin and genistein by preparative chromatography

Purification of the reaction mixtures was performed using a Waters preparative HPLC-PDA-ESI-MS system, consisting of a 2545 quaternary gradient pump, 2767 sample manager, fluid organizer, 2998 photodiode array detector, and 3100 mass spectrometer (Waters, Milford, MA, USA). The reaction mixtures were separated on a Waters XBridge Prep C18 OBD column (19 × 250 mm, 5 µm particle size). Eluents used were water (A) and HPLC grade acetonitrile (B), both acidified with 1% (*v/v*) formic acid. Specific optimized methods were employed to separate the naringenin and genistein reaction mixtures.

The naringenin reaction mixture was resolubilized in 50% (*v/v*) aqueous acetonitrile to a concentration of approximately 76 mg mL^{-1}. Multiple injections (2.1-3.1 mL) were performed. Separation of the naringenin reaction mixture was performed using a flow rate of 17 mL min^{-1}. The elution program was as follows: isocratic at 45% B for 3.50 min, followed by 3.50–21.00 min linear gradient to 65% B, 21.00–50.76 min linear gradient to 82% B, 50.76-54.26 min linear gradient to 100% B, and 54.26–71.76 min isocratic at 100% B. The column was then readjusted to the starting conditions in 3.50 min and equilibrated for 17.50 min.

The genistein reaction mixture was resolubilized in pure DMSO to a concentration of approximately 134 mg mL^{-1}. Multiple injections (1.1-1.9 mL) were performed. "At column dilution" was used to be able to load the samples dissolved in DMSO. To this end, the sample was injected into a flow of 5 mL min^{-1} of eluent B delivered by a loading pump (HPLC pump 515, Waters), which was then mixed, via a T-piece just before the column, with a flow of 12 mL min^{-1} from the gradient pump for a total flow rate of 17 mL min^{-1}. Separation of the

genistein reaction mixture was performed using the following elution program: isocratic at 45% B for 3.50 min, followed by 3.50–25.38 min linear gradient to 70% B, 25.38–53.38 min linear gradient to 82% B, 53.38–56.89 min linear gradient to 100% B, and 56.89–74.39 min isocratic at 100% B. The column was then readjusted to the starting conditions in 3.50 min and equilibrated for 17.50 min.

After the column, a small part of the flow (1:5,000 splitter) was directed to the PDA detector and the MS using a flow (1 mL min^{-1}) of MeOH with 0.1% (*v/v*) formic acid delivered by a make-up pump (HPLC pump 515, Waters). The PDA detector was set to measure a range of 200–600 nm. MS data were collected in the range of *m/z* 200-800 in positive ionization mode. Data acquisition and reprocessing were done with MassLynx (version 4.1, Waters). Fractions were collected and those potentially containing compounds of interest, based on PDA and MS responses, were further analyzed by UHPLC-PDA-ESI-IT-MS^n (see **Supporting Information A3**, using a shortened gradient). Fractions containing the same compound of interest were pooled. Then, the organic solvent was removed under reduced pressure and the remaining water was removed by lyophilization, yielding the pure prenylated naringenin and genistein derivatives.

Supporting Information A3. Reversed-phase ultra-high performance liquid chromatography (RP-UHPLC-PDA) coupled to electrospray ionization ion trap mass spectrometry (ESI-IT-MS^n)

All purified reaction products were subjected to RP-UHPLC-PDA-ESI-IT-MS^n analysis. In addition to the purified reaction products, also all other standards used within this study were analyzed to confirm their identity. **Table S1** shows the results of RP-UHPLC-PDA-ESI-

IT-MS^n analysis. Prenylated phenolics were solubilized in MeOH at a concentration of 50 µg mL^{-1} prior to analysis (after centrifugation for 5 min, 15,000 × *g* at RT). Separation was achieved on a Thermo Vanquish UHPLC system (Thermo Scientific, San Jose, CA, USA) equipped with a pump, degasser, autosampler and photodiode array (PDA) detector. The flow rate was 400 µL min^{-1} and column temperature set at 45 °C. Injection volume was 1 µL. Eluents used were water acidified with 0.1% (*v/v*) FA (A) and acetonitrile acidified with 0.1% (*v/v*) FA (B). An Acquity UPLC BEH C18 column (150 mm × 2.1 mm, i.d. 1.7 µm) with VanGuard guard column (5 mm × 2.1 mm, i.d. 1.7 µm) of the same material (Waters, Milford, USA) were used. The elution program was started by running isocratic at 25% B for 1.10 min, followed by 1.10−36.95 min linear gradient to 99% B, and 36.95−42.44 min isocratic at 99% B. Eluent was adjusted to its starting conditions in 1.09 min, followed by equilibration of 5.49 min. The PDA detector was set to detect wavelengths between 190 and 680 nm.

RP-UHPLC flow was directed to an LTQ Velos Pro linear ion trap mass spectrometer (Thermo Scientific) equipped with a heated ESI probe coupled in-line to the Vanquish UHPLC system after 2.0 min. Nitrogen was used as sheath gas (50 arbitrary units), auxiliary gas (13 arbitrary units), and sweep gas (1 arbitrary units). Both negative ionization (NI) and positive ionization (PI) mode data was acquired in the range of *m/z* 200−1,000. Data dependent MS^2 analyses were performed by collision-induced dissociation with a normalized collision energy of 35% on the most intense ion in full MS. For positive ionization mode, additional data dependent MS^3 spectra were acquired for the most intense product ion in MS^2. Dynamic exclusion, with a repeat count of 3, repeat duration of 5.0 s, and an exclusion duration of 5.0 s was used to obtain MS^2 spectra of multiple different ions present in full MS at the same time. Ion transfer tube temperature was 263 °C, source heater temperature 425 °C, and the source

voltage was 3.5 (PI) and 2.5 (NI) kV. Data were processed using Xcalibur 4.1 (Thermo Scientific).

Supporting Information A4. NMR spectroscopy

In addition to RP-UHPLC-PDA-IT-ESI-MS^n analysis, all synthesized compounds were subjected to nuclear magnetic resonance (NMR) spectroscopy. NMR spectra were recorded using a Bruker® Avance Ultrashield Plus (^{1}H-NMR, 500 MHz; ^{13}C-NMR, 125 MHz respectively) spectrometer with samples dissolved in acetone-d_6. Chemical shifts are reported in (δ) ppm and were referenced to the acetone-d_6 signal (δ_H 2.05 ppm; δ_C 29.84 ppm). Assignments were based on ^{1}H-NMR, ^{13}C-NMR, COSY, HMBC, and HMQC or HSQC experiments. Spectra were analyzed using MestReNova 14.3.1 (Mestrelab research, Santiago de Compostela, Spain). **Figure S1-55** shows ^{1}H (500 MHz) and ^{13}C (125 MHz) NMR spectra and COSY, HMBC, and HMQC or HSQC correlations for all synthesized prenylated phenolics in acetone-*d6*. ^{1}H (500 MHz) and ^{13}C (125 MHz) NMR spectral data is also shown in **Table S2**. Purity was estimated based on ^{1}H NMR (**Table S1**).

Table S1. RP-UHPLC-PDA-IT-MS^n analysis of prenylated phenolics. ^1H NMR purity of purified or synthesized prenylated phenolics is also included. Compound numbers refer to **Figure 1**. sh = shoulder peak, n.d. = not detected, n.a. = not applicable (as commercial standards were not analyzed by NMR).

**Compound**	**Molecular formula**	**λ_{max} (nm)**	**UV_{280} purity (%)**	**^1H NMR purity (%)**	**$[M-H]^-$**	**MS^2 product ions (relative abundance)**	**$[M+H]^+$**	**MS^2 product ions (relative abundance)**	**MS^3 product ions (relative abundance)**
Bavachinin (**1**)	$C_{21}H_{22}O_4$	320	78	n.a.	337	119 (100), 293 (14), 120 (6)	339	271 (100), 283 (98), 267 (24), 284 (18), 219 (18)	177 (100), 253 (36), 151 (28), 147 (27), 225 (27)
Bavachin (**2**)	$C_{20}H_{20}O_4$	278, 322	95	n.a.	323	203 (100)	325	269 (100), 149 (12), 147 (5)	149 (100)
Isobavachin (**3**)	$C_{20}H_{20}O_4$	282, 320sh	94	n.a.	323	203 (100), 119 (9)	325	269 (100), 149 (9), 205 (7)	149 (100), 197 (31), 251 (22)
Glabrol (**4**)	$C_{25}H_{28}O_4$	282	71[a]	72[a,b]	391	203 (100), 187 (26), 159 (6)	393	337 (100), 205 (15), 203 (11), 338 (6), 231 (5)	213 (100), 195 (66), 319 (56), 177 (38), 322 (23)
6-Prenylnaringenin (**5**)	$C_{20}H_{20}O_5$	294, 338sh	94	n.a.	339	219 (100), 245 (9), 220 (8). 233 (6)	341	285 (100)	165 (100), 191 (20)
8-Prenylnaringenin (**6**)	$C_{20}H_{20}O_5$	294, 338	92	95	339	219 (100), 245 (9), 220 (6), 233 (6)	341	285 (100), 286 (9), 165 (8)	165 (100), 191 (16), 121 (5)
3'-Prenylnaringenin (**7**)	$C_{20}H_{20}O_5$	286	93	82	339	151 (100), 177 (18), 187 (13), 107 (7), 161 (6)	341	285 (100), 267 (37), 179 (31), 215 (9), 153 (9)	267 (100), 165 (26), 153 (17), 221 (6)
6,8-Diprenylnaringenin (**8**)	$C_{25}H_{28}O_5$	294, 350	81	85	407	287 (100), 288 (13), 301 (11), 313 (7)	409	353 (100)	297 (100), 233 (29), 259 (5)
Isoxanthohumol (**9**)	$C_{21}H_{22}O_5$	290, 330sh	93	n.a.	353	233 (100), 247 (10)	355	299 (100), 235 (32), 179 (26), 300 (6)	179 (100), 193 (11), 257 (6)
7-*O*-Prenylnaringenin (**10**)	$C_{20}H_{20}O_5$	290, 330sh	96	99	339	270 (100), 219 (58), 193 (21), 271 (11), 164 (11)	341, 273	273 (100)	153 (100), 147 (97), 189 (6), 231 (6)
6-*C*,7-*O*-Diprenylnaringenin (**11**)	$C_{25}H_{28}O_5$	294, 342sh	99	97	407	338 (100), 287 (24), 261 (21), 339 (8), 218 (7)	409	341 (100), 353 (53), 285 (16)	285 (100)
8-*C*,7-*O*-Diprenylnaringenin (**12**)	$C_{25}H_{28}O_5$	294, 346sh	98	97	407	338 (100), 287 (14), 261 (12), 339 (9), 232 (8)	409	341 (100), 285 (100)	285 (100)
3'-*C*,7-*O*-Diprenylnaringenin (**13**)	$C_{25}H_{28}O_5$	290, 342sh	99	84	407	338 (100), 339 (12), 232 (8), 218 (5)	409	341 (100), 353 (30)	285 (100), 267 (35), 179 (31), 153 (8), 215 (7)

7,4'-*O*-Diprenylnaringenin (**14**)	$C_{25}H_{28}O_5$	290, 330	99	99	407	338 (100), 339 (6)	409	341 (100)	273 (100)
4'-*O*-Methylglabridin (**15**)	$C_{21}H_{22}O_4$	278	98[c]	81[c]	337	201 (100), 175 (56), 322 (45), 213 (12), 149 (10)	339	189 (100), 137 (63), 215 (5)	147 (100), 171 (20), 161 (6)
Licorisoflavan A (**16**)	$C_{27}H_{34}O_5$	282	82[a]	86 [a,b]	437	405 (100), 203 (63), 215 (60), 177 (23), 221 (17)	439	383 (100), 371 (54), 315 (10), 369 (9), 235 (6)	181 (100), 189 (83), 327 (60), 191 (25), 193 (18)
Glyasperin C (**17**)	$C_{21}H_{21}O_5$	284	75	n.a.	355	323 (100), 233 (20), 207 (18), 219 (16), 245 (15)	357	301 (100), 211 (50), 235 (30), 289 (7)	179 (100), 165 (52), 123 (34), 191 (19), 283 (17)
Glabridin (**18**)	$C_{20}H_{20}O_4$	278	97	n.a.	323	135 (100), 201 (69), 121 (37), 213 (37). 147 (28)	325	189 (100), 123 (32), 203 (22), 215 (8)	147 (100), 171 (24), 161 (7)
Licoricidin (**19**)	$C_{26}H_{32}O_5$	282	73[a]	94[a]	423	229 (100), 193 (53), 391 (10)	425	369 (100), 221 (24), 191 (13)	189 (100), 167 (90), 313 (36), 191 (20), 219 (19)
Hispaglabridin A (**20**)	$C_{25}H_{28}O_4$	282, 315sh	86[a]	85[a]	391	203 (100), 177 (56), 201 (48), 189 (39), 215 (38)	393	337 (100), 191 (90), 189 (87), 135 (8), 147 (6)	175 (100), 189 (82), 187 (29), 201 (20), 295 (18)
Hispaglabridin B (**21**)	$C_{25}H_{26}O_4$	278	90[c]	≥95[c]	389	201 (100), 175 (23), 374 (8), 187 (7), 213 (7)	391	189 (100), 147 (9)	147 (100), 171 (20), 161 (7)
Neobavaisoflavone (**22**)	$C_{20}H_{18}O_4$	262, 310	85	n.a.	321	265 (100), 321 (75), 266 (20), 322 (14), 277 (11)	323	267 (100), 268 (6)	239 (100), 211 (11), 137 (8), 183 (7)
Wighteone (**23**)	$C_{20}H_{18}O_5$	266, 330	80	n.a.	337	282 (100), 283 (17), 337 (15)	339	283 (100)	283 (100), 265 (25), 255 (20), 121 (6), 199 (5)
α-Isowighteone (**24**)	$C_{20}H_{18}O_5$	284, 312	92	n.a.	337	293 (100), 322 (37), 227 (319), 309 (9)	339	323 (100), 163 (94), 324 (36), 321 (30), 135 (9)	295 (100), 296 (31), 305 (30), 306 (10), 213 (9)
Lupiwighteone (**25**)	$C_{20}H_{18}O_5$	266, 338	84	n.a.	337	282 (100), 283 (10), 337 (8)	339	283 (100), 284 (14)	241 (100), 283 (14), 213 (12), 242 (9), 255 (8)
Isowighteone (**26**)	$C_{20}H_{18}O_5$	260, 324	92	n.a.	337	337 (100), 281 (89), 338 (35), 282 (21), 293 (12)	339	283 (100), 271 (18), 284 (7)	255 (100), 153 (9), 227 (6)
Glabrone (**27**)	$C_{20}H_{16}O_5$	265, 310sh	79[a]	66[a,b]	335	291 (100), 292 (20), 320 (18), 307 (9), 317 (7)	337	295 (100), 309 (34), 319 (33), 283 (32), 296 (16)	267 (100), 295 (33), 268 (21), 277 (9), 266 (6)
6,8-Diprenylgenistein (**28**)	$C_{26}H_{25}O_5$	270, 346	78	n.a.	405	350 (100), 307 (46), 405 (28), 351 (17), 295 (11)	407	351 (100), 295 (6)	295 (100)

Luteone (**29**)	$C_{20}H_{18}O_6$	266, 290sh	93	n.a.	353	309 (100), 219 (88), 285 (66), 298 (46), 201 (44)	355	299 (100)	165 (100), 281 (53)
Licoisoflavone A (**30**)	$C_{20}H_{18}O_6$	262	86	n.a.	353	285 (100), 284 (58), 267 (16), 309 (6), 243 (6)	355	299 (100)	271 (100), 147 (74), 243 (64), 217 (48), 245 (47)
Licoisoflavone B (**31**)	$C_{20}H_{16}O_6$	262	85[a]	83[a,b]	351	283 (100), 284 (18), 265 (13), 307 (9)	353	311 (100), 325 (30), 299 (23), 335 (20), 312 (16)	283 (100), 255 (66), 311 (32), 153 (25), 284 (14)
Glycyrrhisoflavone (**32**)	$C_{20}H_{18}O_6$	262	79	n.a.	353	298 (100), 297 (61), 284 (46), 353 (25), 299 (9)	355	299 (100), 287 (45)	271 (100), 243 (35)
6'-Prenylpiscidone (**33**)	$C_{26}H_{28}O_7$	254, 294	75	n.a.	451	367 (100), 382 (29), 436 (14), 368 (13)	453	397 (100), 385 (68), 329 (47), 317 (16), 411 (10)	369 (100), 341 (27), 329 (9), 355 (9), 354 (8)
7-*O*-Prenylgenistein (**34**)	$C_{20}H_{18}O_5$	262	99	99	337	n.d.	339	271 (100)	271 (100), 215 (82), 153 (78), 243 (74), 253 (33)
6-*C*,7-*O*-Diprenylgenistein (**35**)	$C_{26}H_{25}O_5$	266, 338	98	88	405	350 (100), 335 (51), 405 (43), 336 (27), 351 (20)	407	339 (100), 351 (32), 295 (10), 283 (6)	283 (100)
8-*C*-,7-*O*-Diprenylgenistein (**36**)	$C_{26}H_{25}O_5$	266, 338	97	86	405	336 (100), 349 (6), 337 (5)	407	339 (100)	283 (100)

[a] Based on ^{1}H NMR purity reported by van Dinteren et al. [1], without considering residual solvents or additives from the purification process (*i.e.*, *tert*-butanol and formic acid). [b] If the estimated ^{1}H NMR purity including *tert*-butanol and formic acid as impurity was ≤ 75% (*i.e.*, compounds **4**, **16**, **27**, and **31**), the content was corrected as described in [1]. [c] Based on purity reported by van de Schans et al. [2]

Table S2. ^{1}H (500 MHz) and ^{13}C (125 MHz) NMR spectral data for synthesized prenylated phenolics in acetone-*d6*. Assignments were based on ^{1}H and ^{13}C NMR, COSY, HMBC, and HMQC or HSQC 2D spectra. Compound numbers refer to **Figure 1**. Chemical shifts (δ_C and δ_H) are reported in ppm. Pos. = position, mult. = multiplicity, int. = integral, ax = axial, eq = equatorial, s = singlet, d = doublet, dd = doublet of doubles, t = triplet, m = multiplet.

	*8-Prenylnaringenin (**6**)*		*3´-Prenylnaringenin (**7**)*		*6,8-Diprenylnaringenin (**8**)*		*7-O-Prenylnaringenin (**10**)*	
Pos.	**δ_C**	**δ_H (mult.; *J* [Hz]; int.)**	**δ_C**	**δ_H (mult.; *J* [Hz]; int.)**	**δ_C**	**δ_H (mult.; *J* [Hz]; int.)**	**δ_C**	**δ_H (mult.; *J* [Hz]; int.)**
2	79.75	5.42 (dd; 12.7, 3.1; 1H)	80.15	5.43 (dd; 12.8, 3.1; 1H)	79.70	5.43 (dd; 12.7, 3.0; 1H)	79.96	5.46 (d; 3.1; 1H)
3-eq	43.47	2.74 (dd; 17.1, 3.1; 1H)	43.49	2.72 (dd; 17.1, 3.1; 1H)	43.56	2.77 (dd; 17.0, 3.1; 1H)	43.45	2.74 (dd; 17.1, 3.0; 1H)
3-ax	43.47	3.11 (dd; 17.0, 12.7; 1H)	43.49	3.18 (dd; 17.1, 12.8; 1H)	43.56	3.14 (dd; 17.0, 12.7; 1H)	43.45	3.19 (dd; 17.1, 13.0; 1H)
4	197.53		197.34		197.92		197.47	
5	163.01		165.32		160.17		164.87	
6	96.42	6.02 (s; 1H)	95.84	5.95 (d; 2.2; 1H)	108.70		95.06	6.02 (d; 2.3; 1H)
7	165.08		167.35		162.32		168.06	
8	108.33		96.77	5.95 (d; 2.2; 1H)	107.94		96.02	6.04 (d; 2.3; 1H)
9	161.10		164.42		158.95		164.06	
10	103.29		103.25		103.36		103.60	
1´	131.13		130.84		131.14		130.67	
2´	128.85	7.40 (d; 2.1; 1H)	129.15	7.29 (d; 2.3; 1H)	128.85	7.39 (d; 2.1; 1H)	129.01	7.39 (d; 2.1; 1H)
3´	116.15	6.89 (d; 2.1; 1H)	128.97		116.13	6.91 (s; 1H)	116.16	6.91 (d; 2.0; 1H)
4´	158.58		156.20		158.57		158.67	
5´	116.15	6.88 (d; 2.1; 1H)	115.70	6.89 (d; 8.2; 1H)	116.19	6.89 (s; 1H)	116.16	6.90 (d; 2.0; 1H)
6´	128.85	7.38 (d; 2.1; 1H)	126.30	7.21 (dd; 8.2, 2.3; 1H)	128.85	7.40 (d; 2.0; 1H)	129.01	7.38 (d; 2.0; 1H)
1´´	22.29	3.20 (d; 7.3; 2H)	29.11	3.35 (d; 7.4; 2H)	21.78	3.32 (dd; 7.3, 2.5; 2H)	66.05	4.60 (d; 6.7; 2H)
2´´	123.75	5.17 (m; 1H)	123.48	5.36 (m; 1H)	123.34	5.18 (m; 1H)	120.03	5.43 (m; 1H)
3´´	131.18		132.68		132.17		138.87	
4´´	17.86	1.59 (d; 1.5; 3H)	17.86	1.71 (s; 3H)	17.94	1.75 (d; 1.5; 3H)	25.77	1.77 (d; 1.5; 3H)
5´´	25.92	1.58 (d; 1.4; 3H)	25.91	1.71 (s; 3H)	25.86	1.65 (d; 1.6; 3H)	18.20	1.74 (d; 1.3; 3H)
1´´´					22.47	3.29 (d; 7.1; 2H)		
2´´´					123.46	5.15 (m; 1H)		
3´´´					131.96			
4´´´					17.94	1.59 (d; 1.4; 3H)		
5´´´					25.89	1.62 (d; 1.5; 3H)		

Table S2. Continued.

	*6-C,7-O-Diprenylnaringenin (**11**)*		*8-C,7-O-Diprenylnaringenin (**12**)*		*3´-C,7-O-Diprenylnaringenin (**13**)*		*7,4-O-Diprenylnaringenin (**14**)*	
Pos.	**δ_C**	**δ_H (mult.; *J* [Hz]; int.)**	**δ_C**	**δ_H (mult.; *J* [Hz]; int.)**	**δ_C**	**δ_H (mult.; *J* [Hz]; int.)**	**δ_C**	**δ_H (mult.; *J* [Hz]; int.)**
2	80.06	5.45 (dd; 13.1, 3.0; 1H)	79.70	5.45 (dd; 12.6, 3.0; 1H)	80.25	5.46 (d; 3.1; 1H)	79.86	5.51 (dd; 12.8, 3.0; 1H)
3-eq	43.60	2.74 (m; 1H)	43.56	2.79 (dd; 17.1, 3.0; 1H)	43.57	2.74 (dd; 17.1, 3.1; 1H)	43.45	2.77 (dd; 17.1, 3.0; 1H)
3-ax	43.60	3.18 (m; 1H)	43.56	3.15 (dd; 17.1, 12.5; 1H)	43.57	3.20 (dd; 17.1, 12.7; 1H)	43.45	3.21 (dd; 17.1, 12.8; 1H)
4	197.68		197.91		197.63		197.45	
5	160.98		163.50		164.98		164.94	
6	110.09		93.97	6.14 (s; 1H)	95.13		96.06	6.04 (d; 2.3; 1H)
7	165.57		165.75		168.17	6.02 (d; 2.3 Hz; 1H)	168.14	
8	92.75	6.15 (s; 1H)	109.45		96.04	6.03 (d; 2.3; 1H)	95.13	6.05 (d; 2.3; 1H)
9	162.61		160.00		164.20		164.05	
10	103.43		103.53		103.73		103.65	
1´	130.80		131.08		130.78		131.70	
2´	129.02	7.40 (d; 8.3; 1H)	128.84	7.40 (d; 8.5; 1H)	129.16	7.29 (d; 2.3; 1H)	128.88	7.48 (m; 1H)
3´	116.18	6.91 (d; 8.5; 1H)	116.18	6.90 (d; 8.7; 1H)	128.85		115.49	6.99 (m; 1H)
4´	158.74		158.61		156.21		160.21	
5´	116.18	6.91 (d; 8.5; 1H)	116.18	6.90 (d; 8.7; 1H)	115.72	6.89 (d; 8.2; 1H)	115.49	6.99 (m; 1H)
6´	129.02	7.40 (d; 8.3; 1H)	128.84	7.40 (d; 8.5; 1H)	126.31	7.21 (dd; 8.2, 2.3; 1H)	128.88	7.48 (m; 1H)
1´´	21.70	3.22 (m; 2H)	66.26	4.64 (d; 6.6; 2H)	66.13	4.62 (d; 6.7; 2H)	66.10	4.62 (d; 6.6; 2H)
2´´	123.52	5.18 (m; 1H)	120.25	5.50 (m; 1H)	120.13	5.44 (dd; 4.8, 2.4; 1H)	120.06	5.44 (m; 1H)
3´´	131.21		138.97		138.92		138.94	
4´´	17.86	1.74 (s; 3H)	18.28	1.77 (s; 3H)	18.21	1.75 (s; 3H)	18.21	1.75 (s; 3H)
5´´	25.90	1.60 (s; 3H)	25.80	1.79 (s; 3H)	25.93	1.77 (s; 3H)	25.79	1.77 (s; 3H)
1´´´	66.23	4.63 (d; 6.8; 2H)	22.36	3.20 (d; 7.4; 2H)	29.12	3.36 (d; 7.3; 2H)	65.44	4.59 (d; 6.6; 2H)
2´´´	120.26	5.49 (m; 1H)	123.67	5.14 (m; 1H)	123.50	5.36 (m; 1H)	120.98	5.47 (m; 1H)
3´´´	138.90		131.20		132.70		137.91	
4´´´	18.27	1.76 (s; 3H)	17.89	1.60 (s; 3H)	17.86	1.72 (s; 3H)	18.21	1.75 (s; 3H)
5´´´	25.79	1.78 (s; 3H)	25.93	1.60 (s; 3H)	25.78	1.72 (s; 3H)	25.79	1.77 (s; 3H)

Table S2. Continued.

	7-*O*-Prenylgenistein (**34**)		6-*C*,7-*O*-Diprenylgenistein (**35**)		8-*C*,7-*O*-Diprenylgenistein (**36**)	
Pos.	**δ_C**	**δ_H (mult.; *J* [Hz]; int.)**	**δ_C**	**δ_H (mult.; *J* [Hz]; int.)**	**δ_C**	**δ_H (mult.; *J* [Hz]; int.)**
2	154.52	8.20 (s; 1H)	154.34	8.22 (s; 1H)	154.66	8.27 (s; 1H)
3-eq	124.21		124.21		123.64	
3-ax						
4	181.75		181.75		182.19	
5	163.59		159.60		162.05	
6	99.40	6.34 (d; 2.2; 1H)	113.41		96.67	6.50 (s; 1H)
7	165.94		163.51		163.16	
8	93.60	6.53 (d; 2.2; 1H)	91.53	6.64 (s; 1H)	108.57	
9	158.91		157.24		155.43	
10	106.74		106.61		106.26	
1´	123.02		123.18		123.17	
2´	131.22	7.46 (m; 1H)	131.70	7.47 (m; 1H)	131.18	7.47 (d; 8.4; 1H)
3´	116.04	6.91 (m; 1H)	115.99	6.91 (m; 1H)	116.01	6.91 (d; 8.5; 1H)
4´	158.51		158.45		158.45	
5´	116.04	6.91 (m; 1H)	115.99	6.91 (m; 1H)	116.01	6.91 (d; 8.5; 1H)
6´	131.22	7.46 (m; 1H)	131.70	7.47 (m; 1H)	131.18	7.47 (d; 8.4; 1H)
1´´	66.35	4.69 (d; 6.7; 2H)	22.11	3.34 (d; 7.3; 2H)	66.55	4.72 (d; 6.6; 2H)
2´´	120.05	5.49 (m; 1H)	123.06	5.22 (m; 1H)	120.26	5.53 (t; 6.8; 1H)
3´´	139.08		131.70		139.07	
4´´	18.28	1.78 (s; 3H)	17.93	1.77 (s; 3H)	17.92	1.80 (s; 3H)
5´´	25.80	1.79 (s; 3H)	25.91	1.64 (; 3H)	25.82	1.81 (s; 3H)
1´´´			66.46	4.72 (d; 6.7; 2H)	22.13	3.43 (d; 7.3; 2H)
2´´´			120.16	5.55 (m; 1H)	123.04	5.20 (t; 7.4; 1H)
3´´´			139.12		132.05	
4´´´			18.31	1.79 (s; 1H)	18.31	1.80 (s; 3H)
5´´´			25.81	1.81 (s; 3H)	25.89	1.65 (s; 3H)

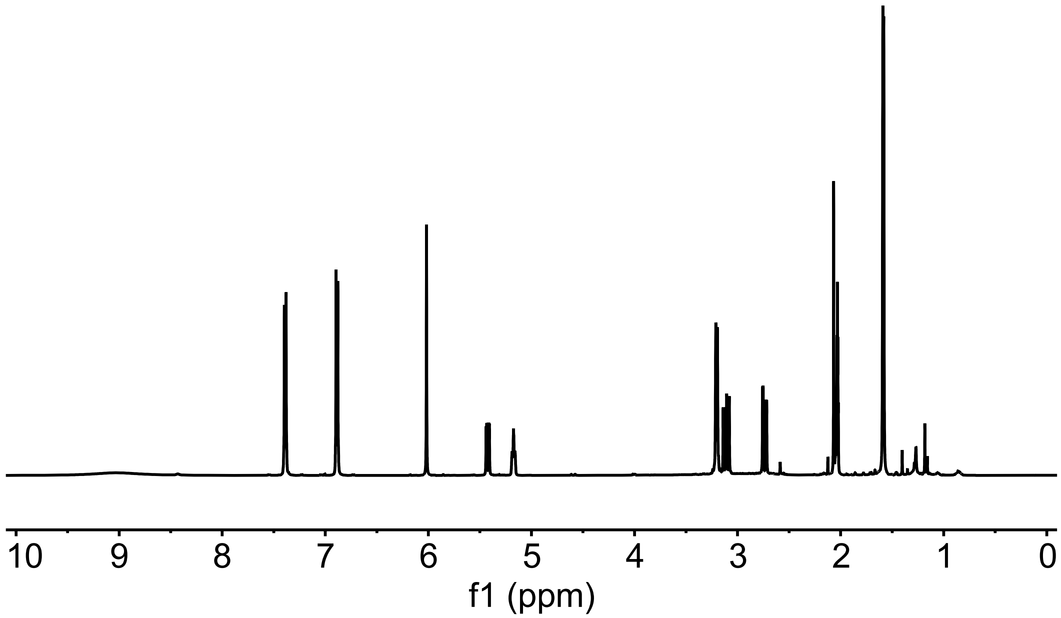

Figure S1. ^{1}H NMR (500 MHz) of 8-prenylnaringenin (**6**) in acetone-*d6*.

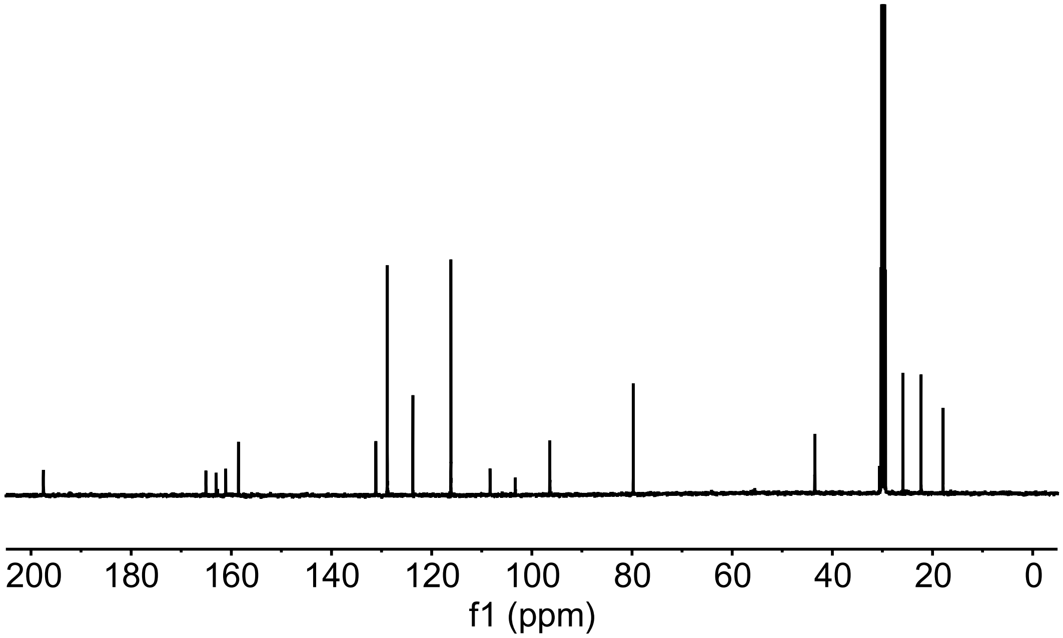

Figure S2. ^{13}C NMR (125 MHz) of 8-prenylnaringenin (**6**) in acetone-*d6*.

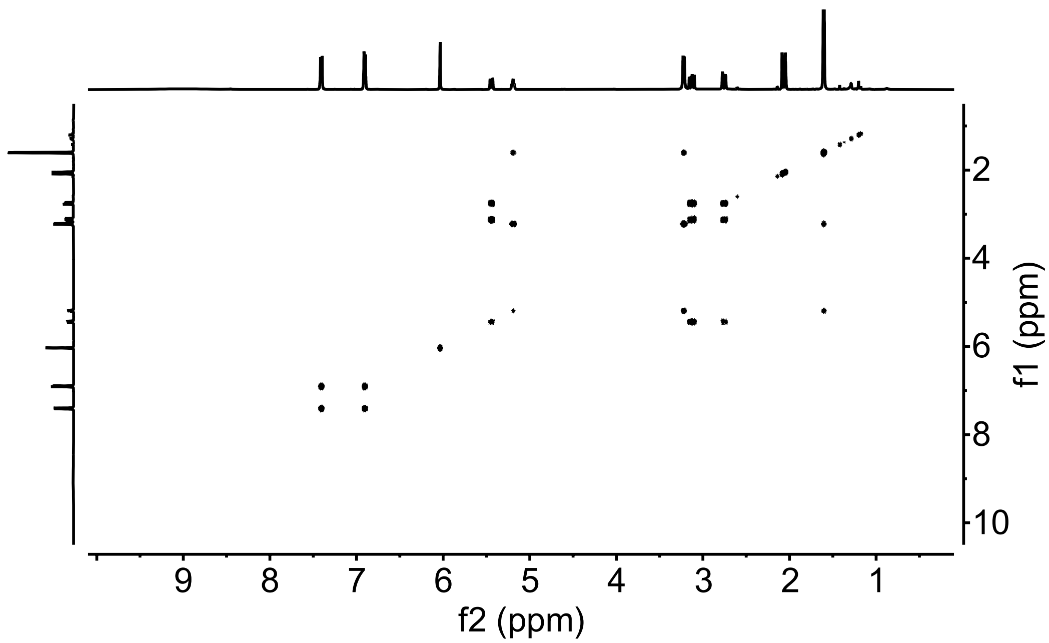

Figure S3. ^{1}H (500 MHz) NMR spectra and COSY correlations of 8-prenylnaringenin (**6**) in acetone-*d6*.

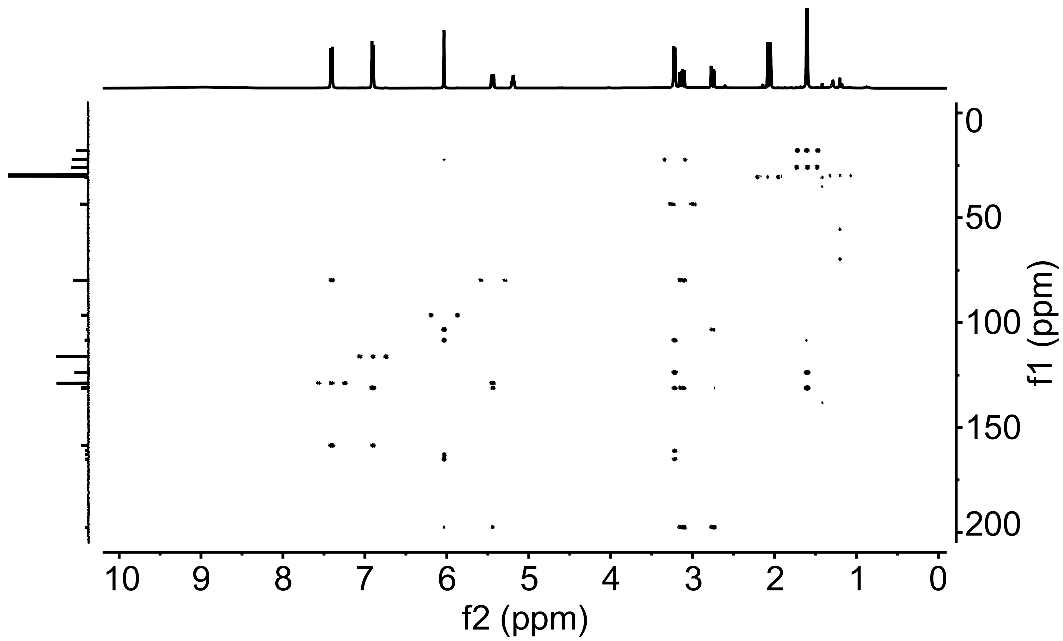

Figure S4. ^{1}H (500 MHz) and ^{13}C (125 MHz) NMR spectra and HMBC correlations of 8-prenylnaringenin (**6**) in acetone-*d6*.

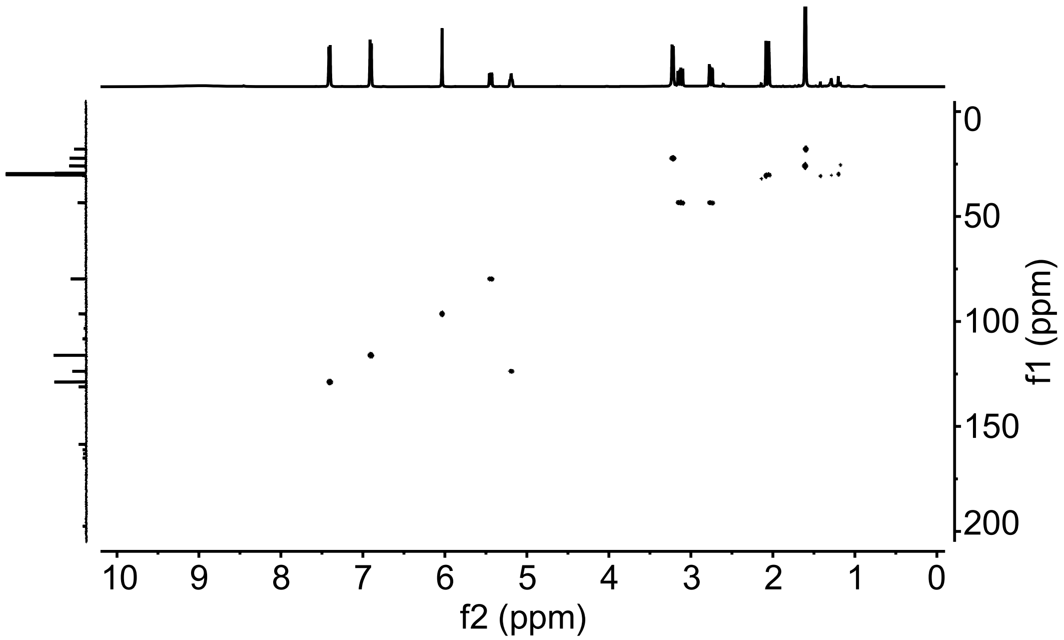

Figure S5. ^{1}H (500 MHz) and ^{13}C (125 MHz) NMR spectra and HMQC correlations of 8-prenylnaringenin (**6**) in acetone-*d6*.

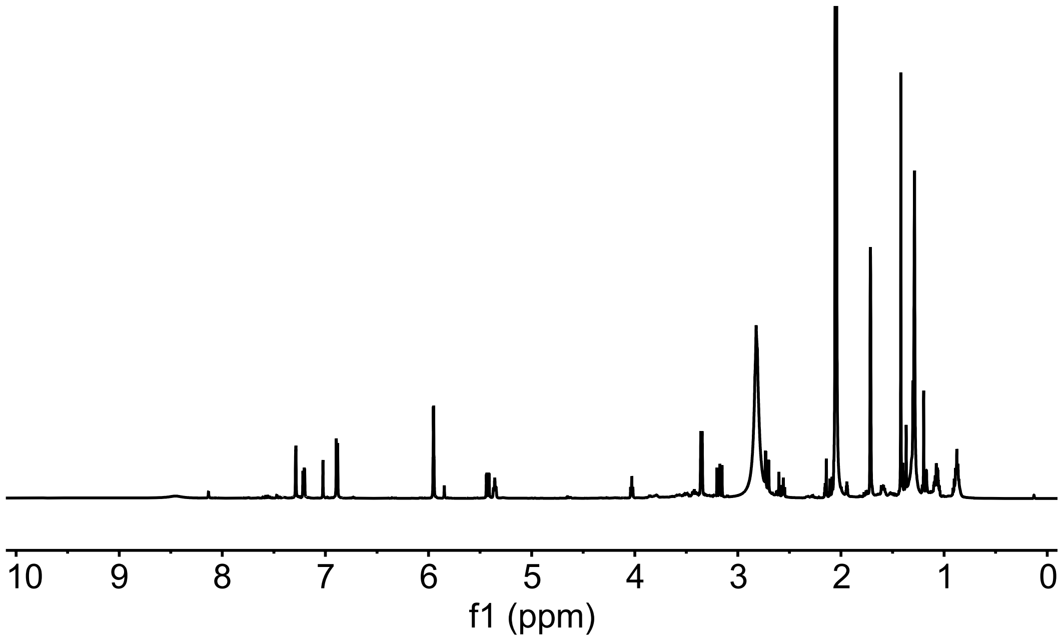

Figure S6. ^{1}H NMR (500 MHz) of 3'-prenylnaringenin (**7**) in acetone-*d6*.

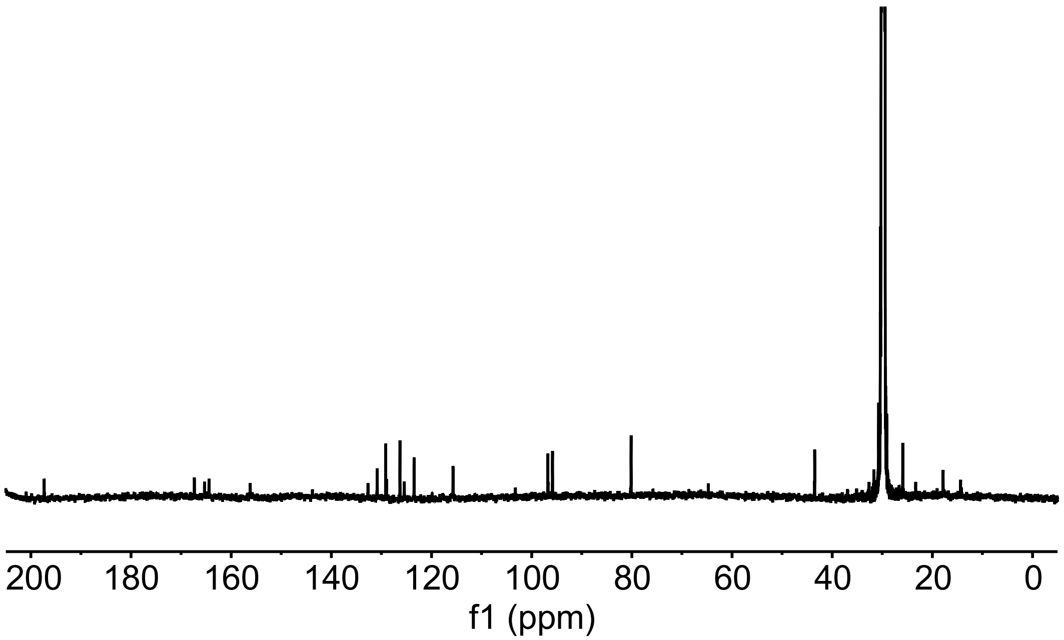

Figure S7. ^{13}C NMR (125 MHz) of 3'-prenylnaringenin (**7**) in acetone-*d6*.

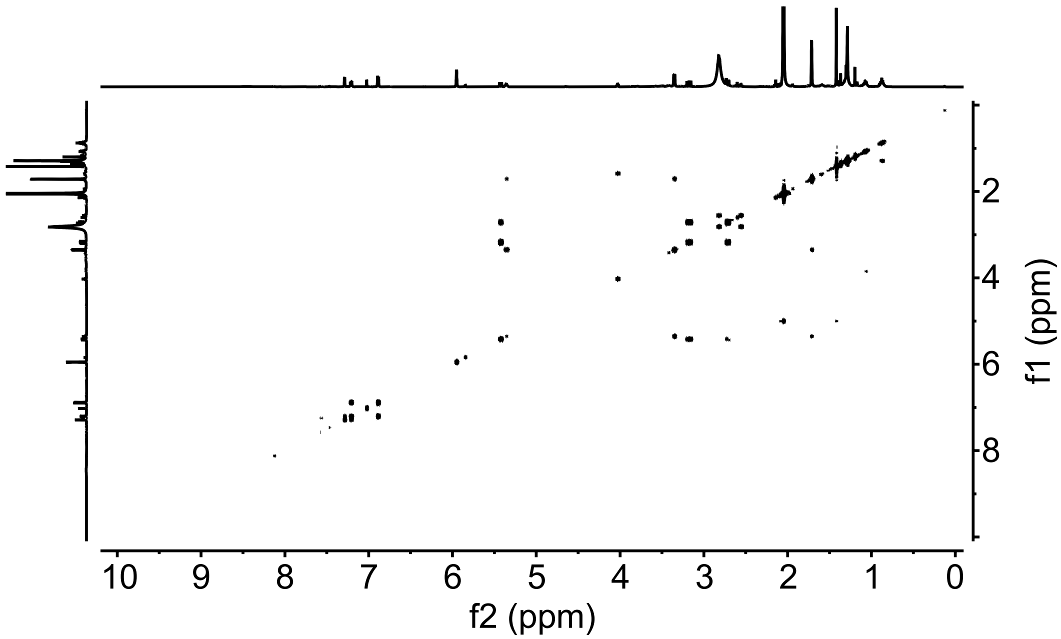

Figure S8. ^{1}H (500 MHz) NMR spectra and COSY correlations of 3'-prenylnaringenin (**7**) in acetone-*d6*.

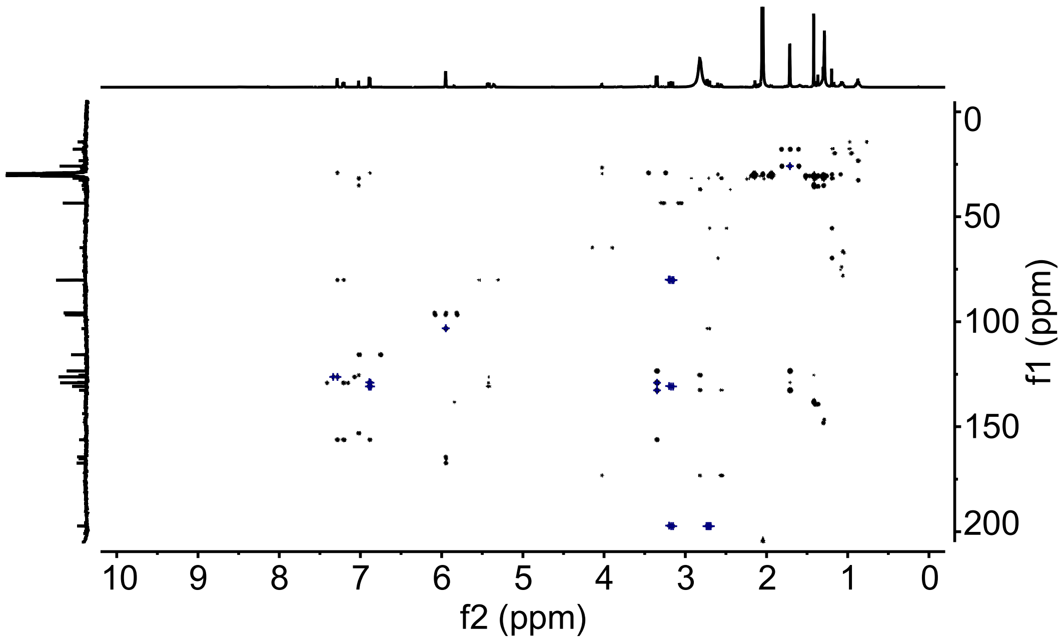

Figure S9. ^{1}H (500 MHz) and ^{13}C (125 MHz) NMR spectra and HMBC correlations of 3’-prenylnaringenin (**7**) in acetone-*d6*.

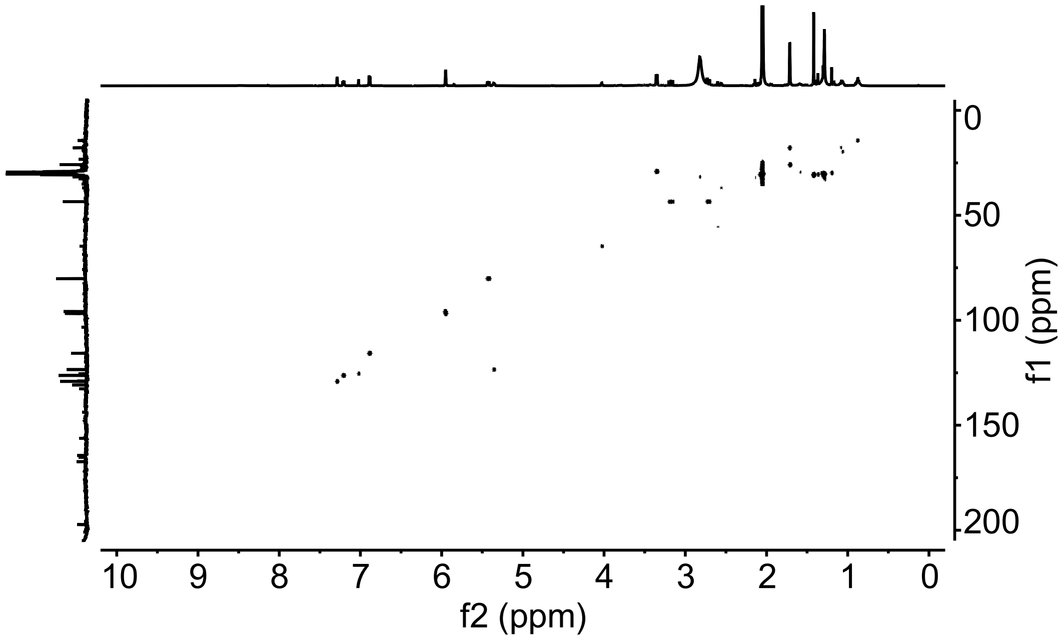

Figure S10. ^{1}H (500 MHz) and ^{13}C (125 MHz) NMR spectra and HSQC correlations of 3’-prenylnaringenin (**7**) in acetone-*d6*.

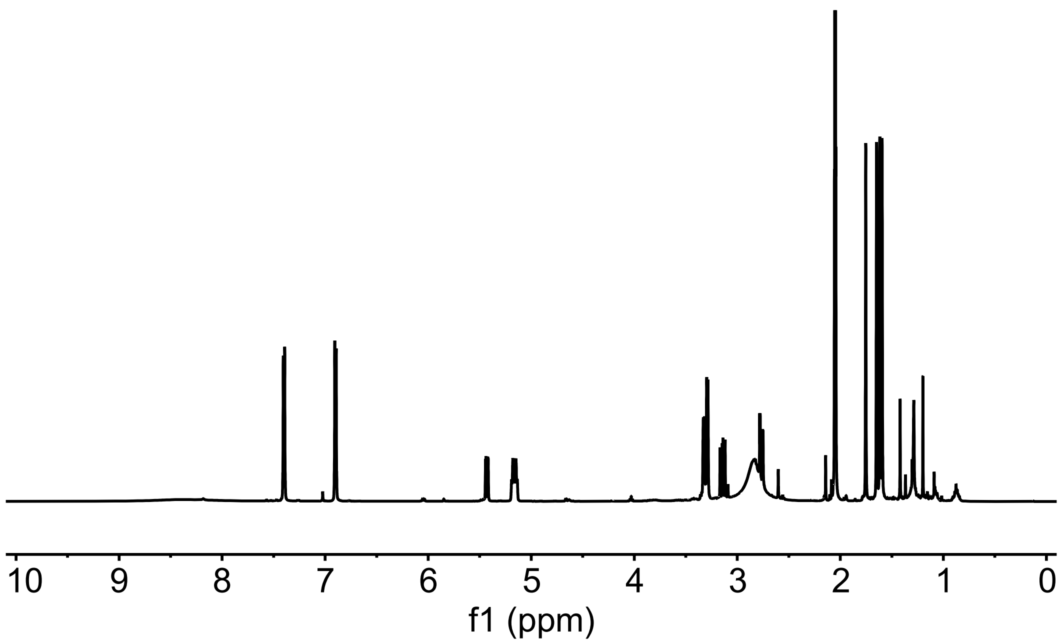

Figure S11. ^{1}H (500 MHz) NMR of 6,8-diprenylnaringenin (**8**) in acetone-*d6*.

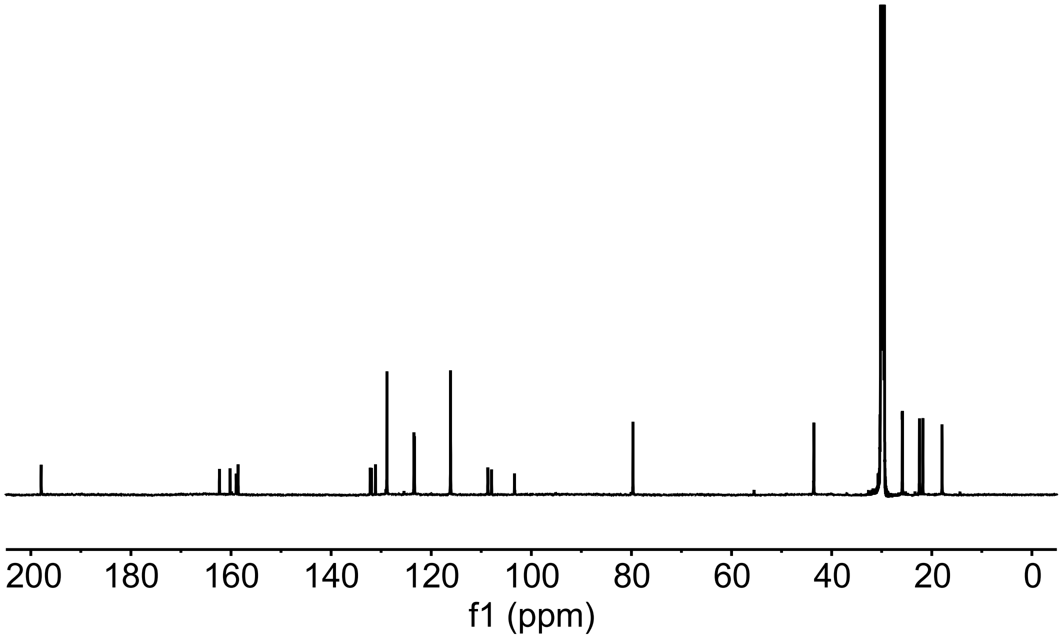

Figure S12. ^{13}C NMR (125 MHz) of 6,8-diprenylnaringenin (**8**) in acetone-*d6*.

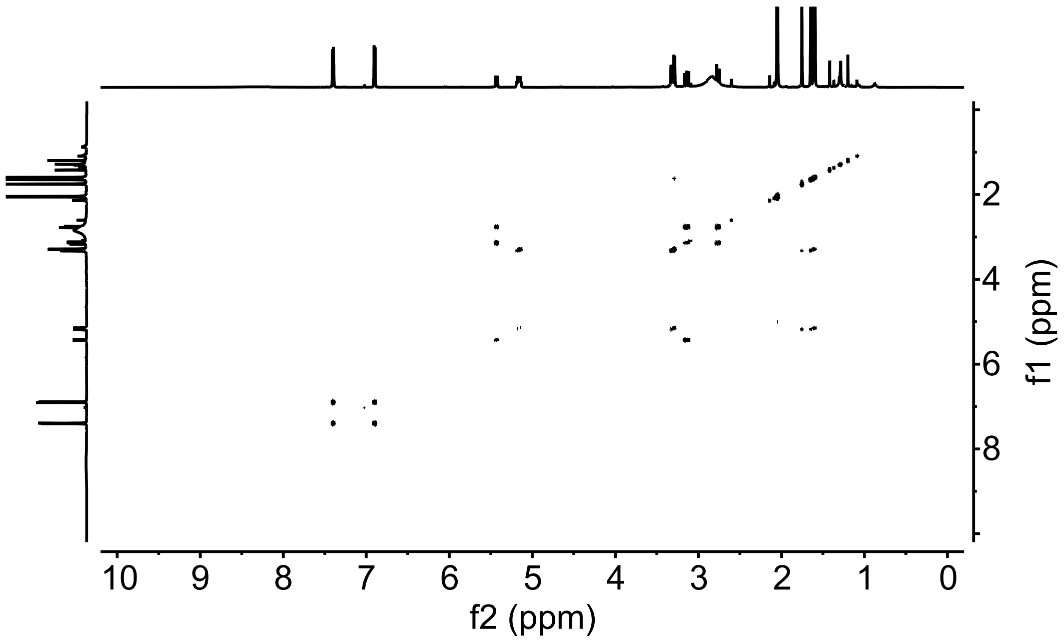

Figure S13. ^{1}H (500 MHz) NMR spectra and COSY correlations of 6,8-diprenylnaringenin (**8**) in acetone-*d6*.

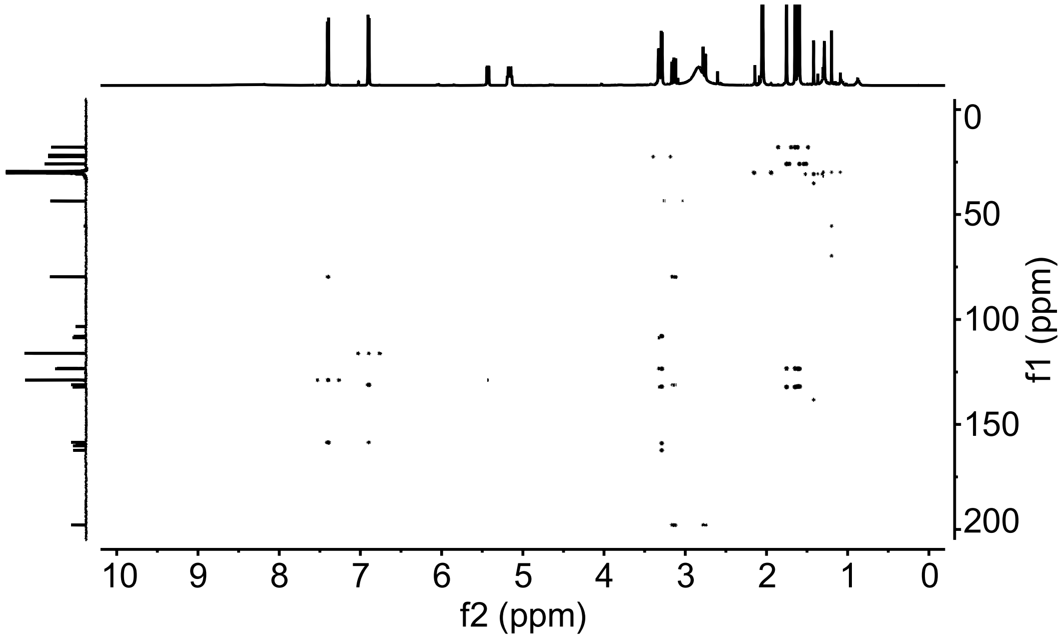

Figure S14. ^{1}H (500 MHz) and ^{13}C (125 MHz) NMR spectra and HMBC correlations of 6,8-prenylnaringenin (**8**) in acetone-*d6*.

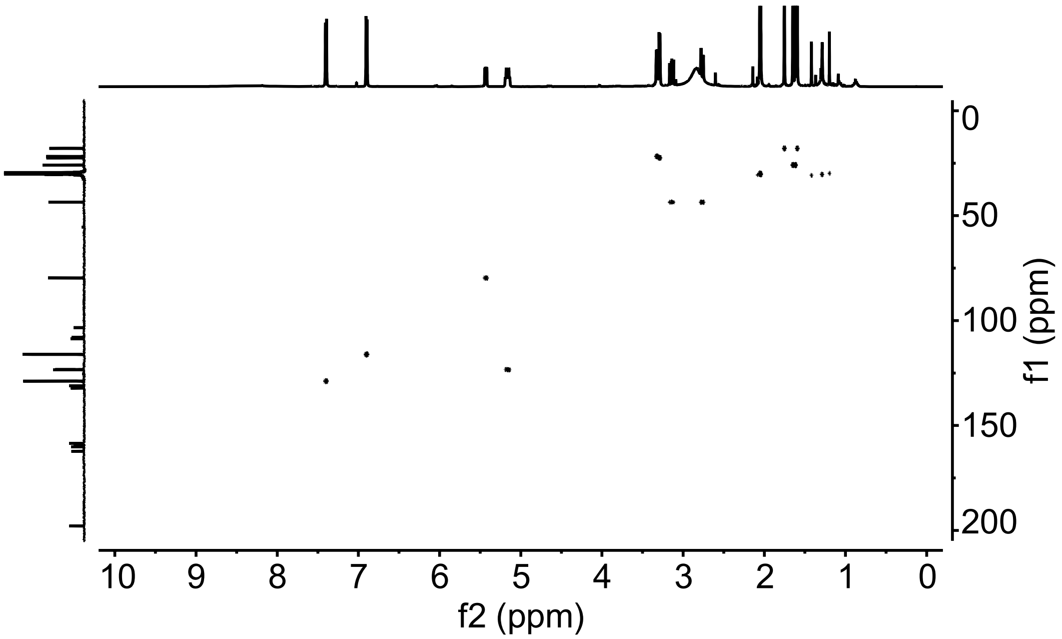

Figure S15. ^{1}H (500 MHz) and ^{13}C (125 MHz) NMR spectra and HSQC correlations of 6,8-prenylnaringenin (**8**) in acetone-*d6*.

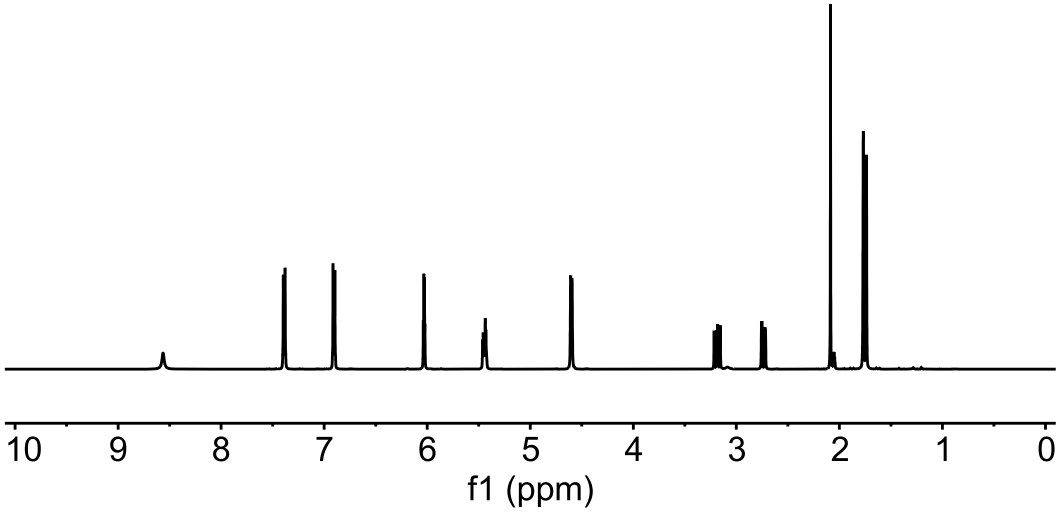

Figure S16. ^{1}H (500 MHz) NMR of 7-*O*-prenylnaringenin (**10**) in acetone-*d6*.

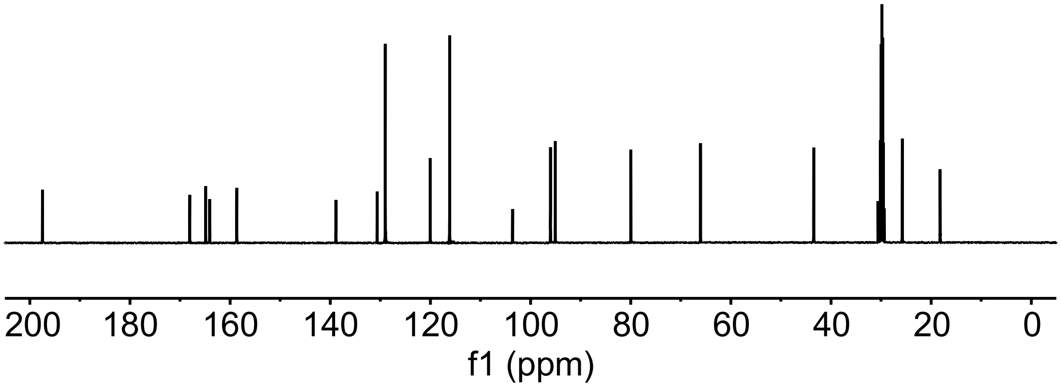

Figure S17. ^{13}C NMR (125 MHz) of 7-*O*-prenylnaringenin (**10**) in acetone-*d6*.

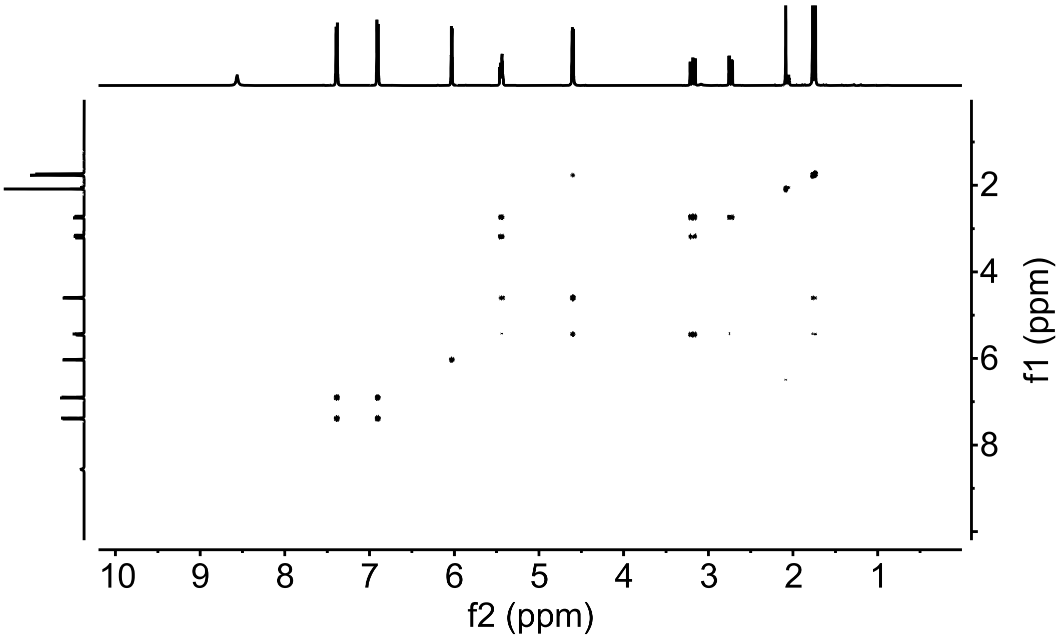

Figure S18. ^{1}H (500 MHz) NMR spectra and COSY correlations of 7-*O*-prenylnaringenin (**10**) in acetone-*d6*.

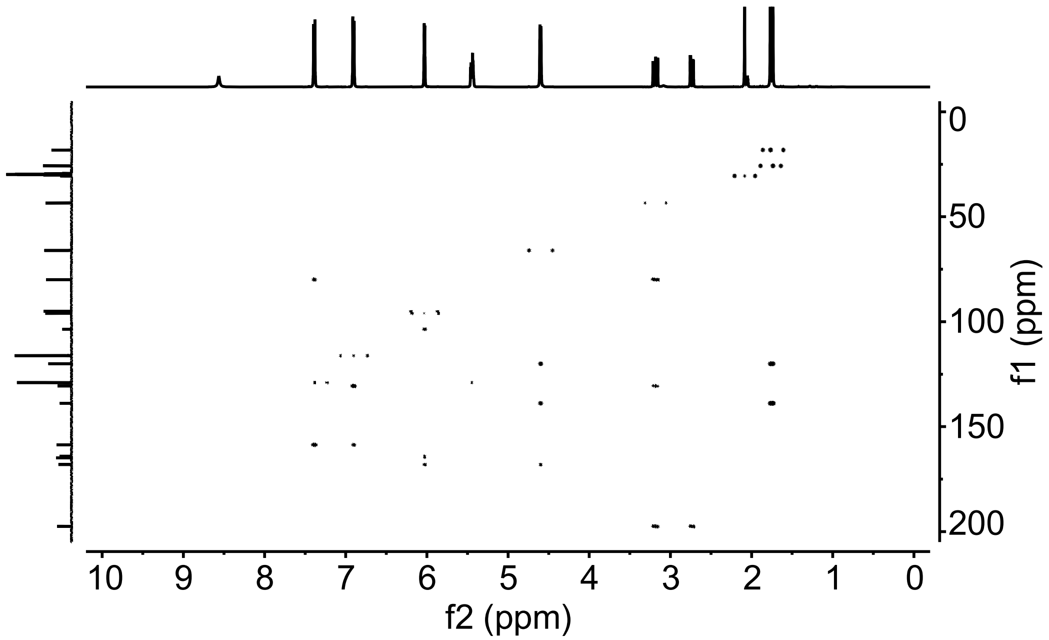

Figure S19. ^{1}H (500 MHz) and ^{13}C (125 MHz) NMR spectra and HMBC correlations of 7-*O*-prenylnaringenin (**10**) in acetone-*d6*.

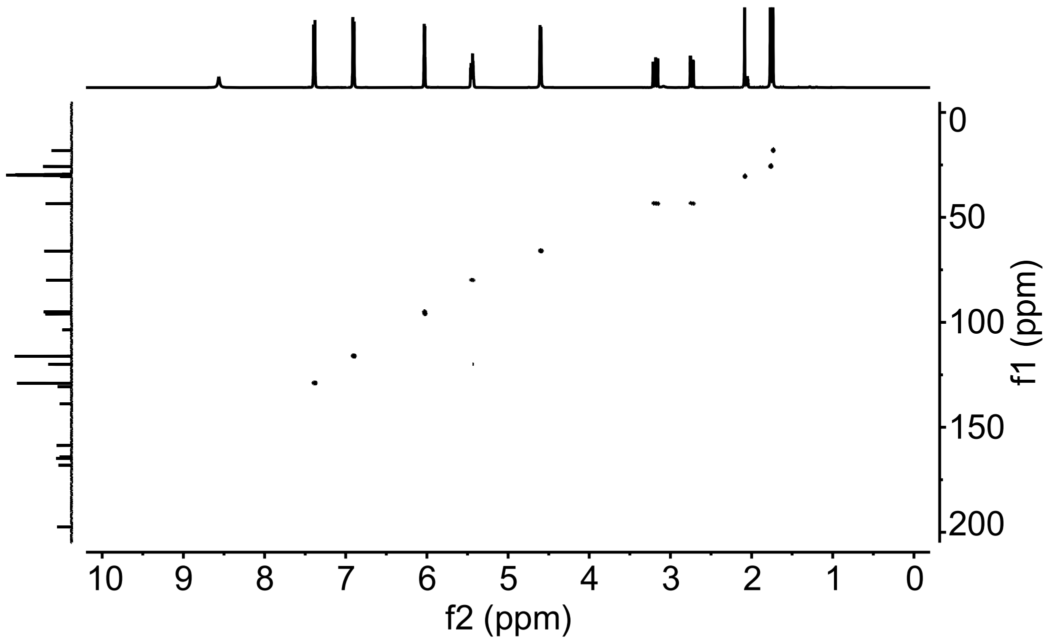

Figure S20. ^{1}H (500 MHz) and ^{13}C (125 MHz) NMR spectra and HMQC correlations of 7-*O*-prenylnaringenin (**10**) in acetone-*d6*.

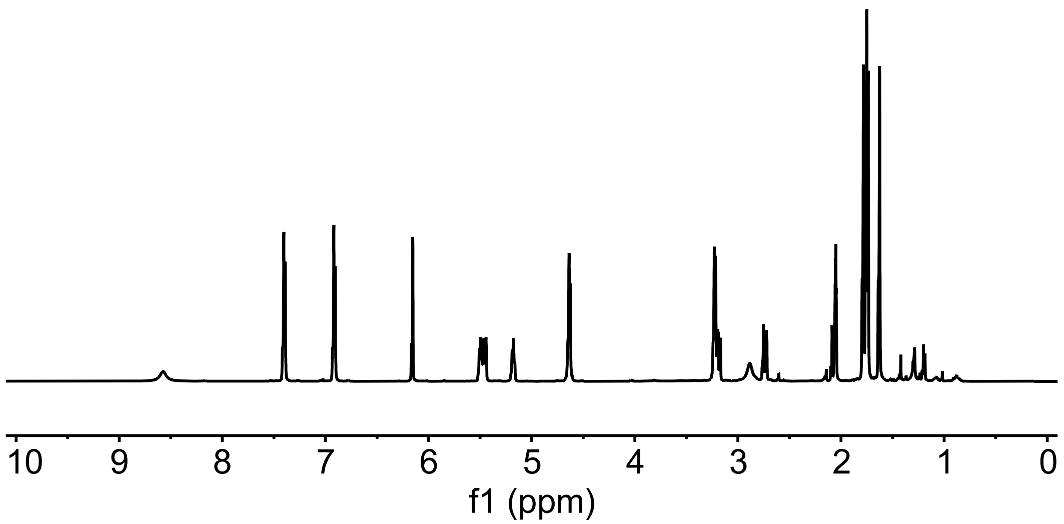

Figure S21. ^{1}H (500 MHz) NMR of 6-*C*,7-*O*-diprenylnaringenin (**11**) in acetone-*d6*.

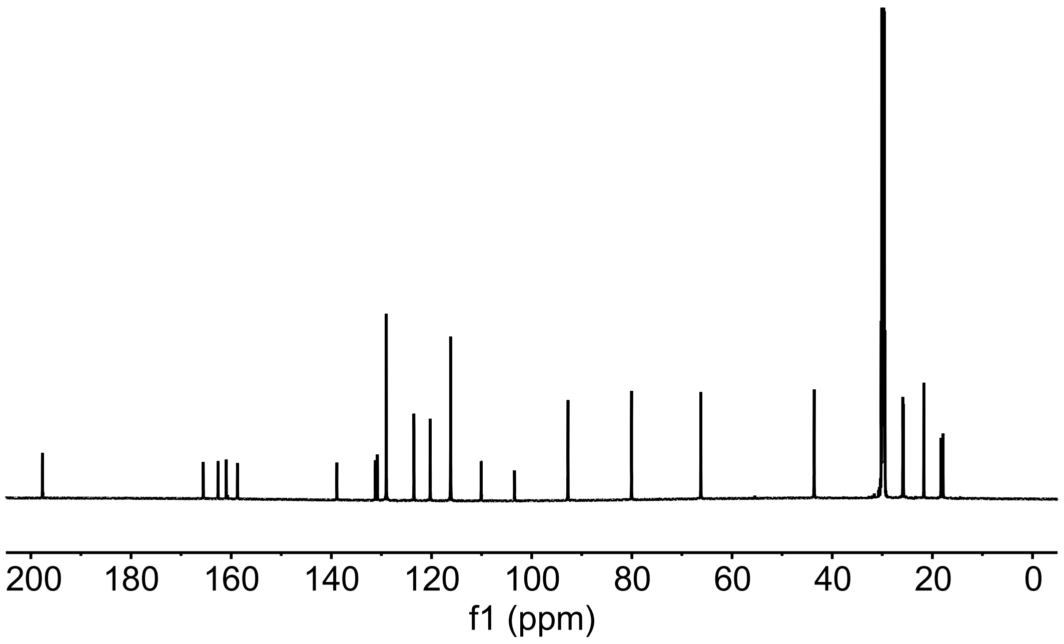

Figure S22. ^{13}C NMR (125 MHz) of 6-*C*,7-*O*-diprenylnaringenin (**11**) in acetone-*d6*.

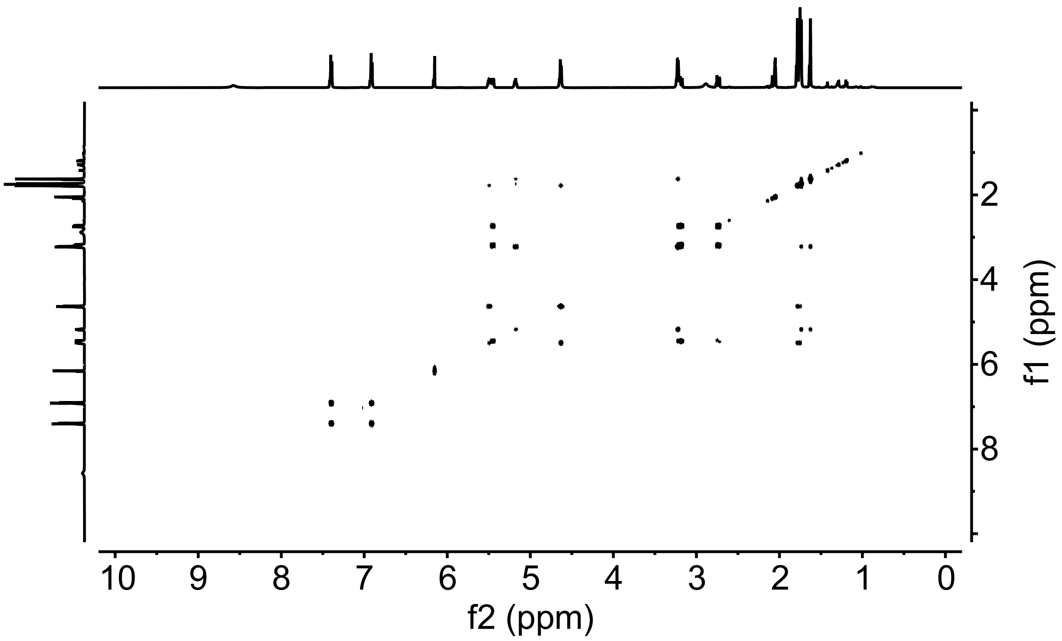

Figure S23. ^{1}H (500 MHz) NMR spectra and COSY correlations of 6-*C*,7-*O*-diprenylnaringenin (**11**) in acetone-*d6*.

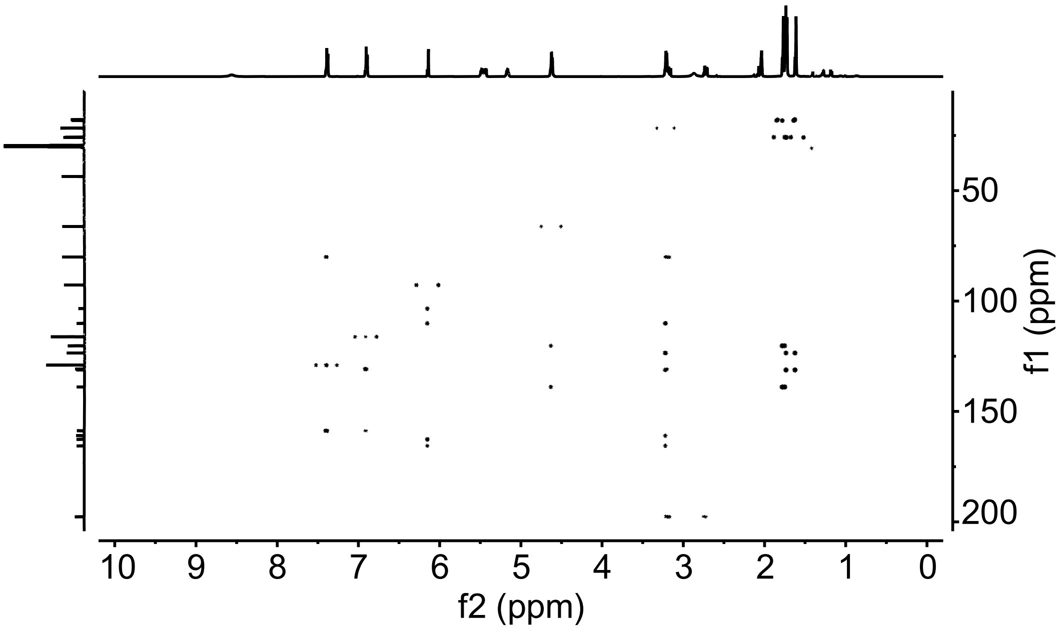

Figure S24. ^{1}H (500 MHz) and ^{13}C (125 MHz) NMR spectra and HMBC correlations of 6-*C*,7-*O*-diprenylnaringenin (**11**) in acetone-*d6*.

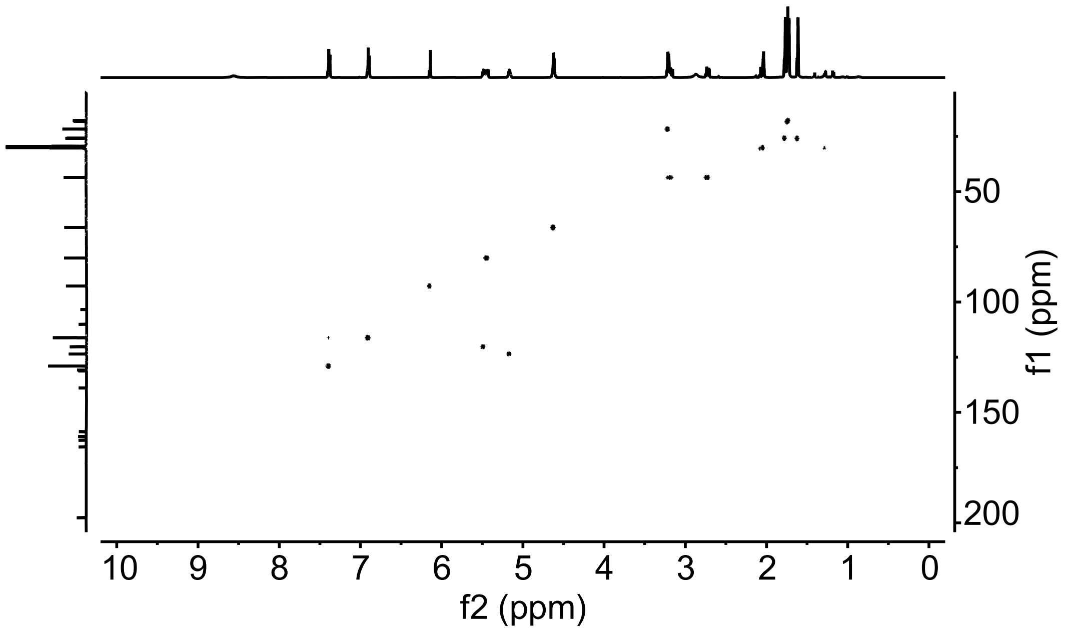

Figure S25. ^{1}H (500 MHz) and ^{13}C (125 MHz) NMR spectra and HSQC correlations of 6-*C*,7-*O*-diprenylnaringenin (**11**) in acetone-*d6*.

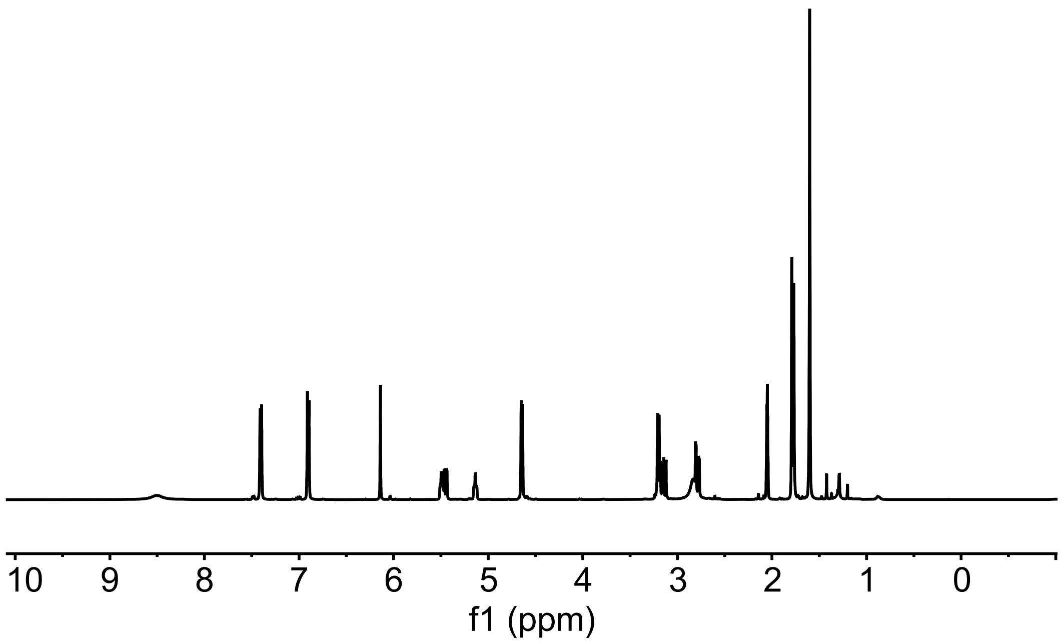

Figure S26. ^{1}H (500 MHz) NMR of 8-*C*,7-*O*-diprenylnaringenin (**12**) in acetone-*d6*.

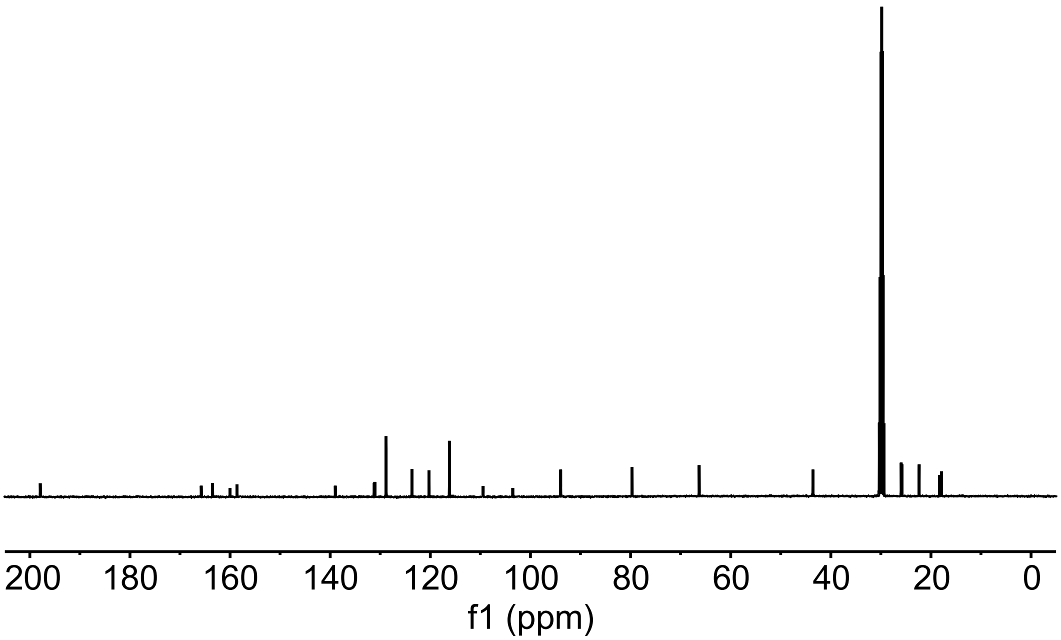

Figure S27. ^{13}C NMR (125 MHz) of 8-*C*,7-*O*-diprenylnaringenin (**12**) in acetone-*d6*.

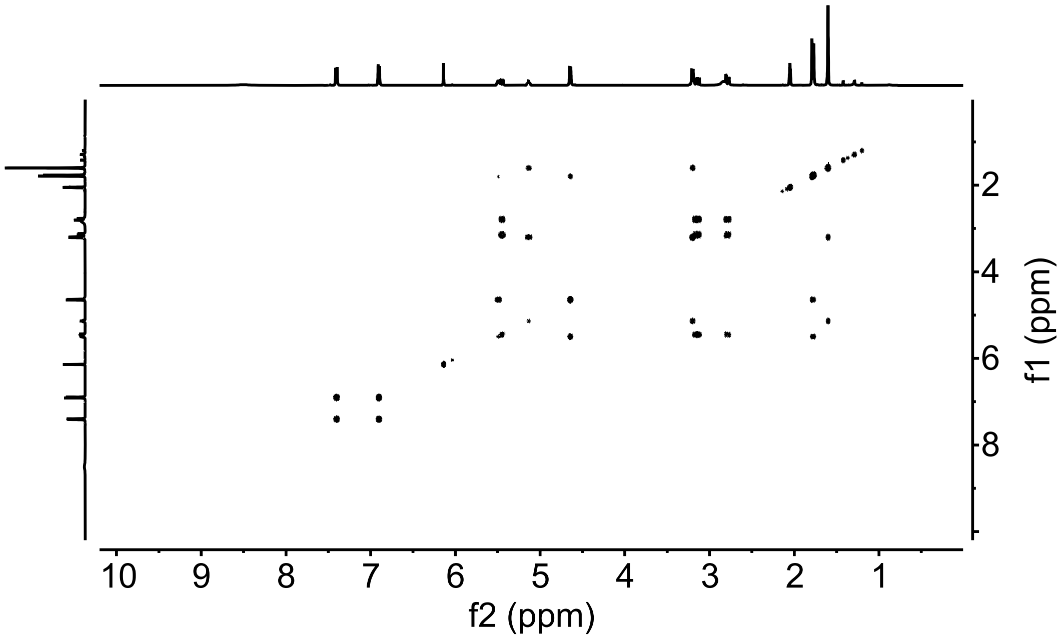

Figure S28. ^{1}H (500 MHz) NMR spectra and COSY correlations of 8-*C*,7-*O*-diprenylnaringenin (**12**) in acetone-*d6*.

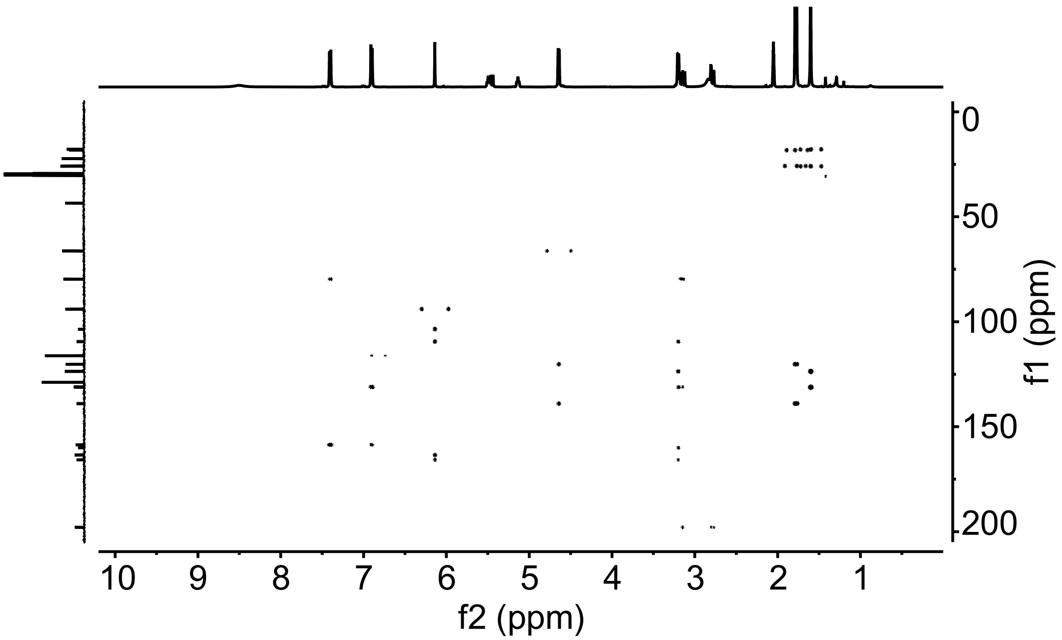

Figure S29. ^{1}H (500 MHz) and ^{13}C (125 MHz) NMR spectra and HMBC correlations of 8-*C*,7-*O*-diprenylnaringenin (**12**) in acetone-*d6*.

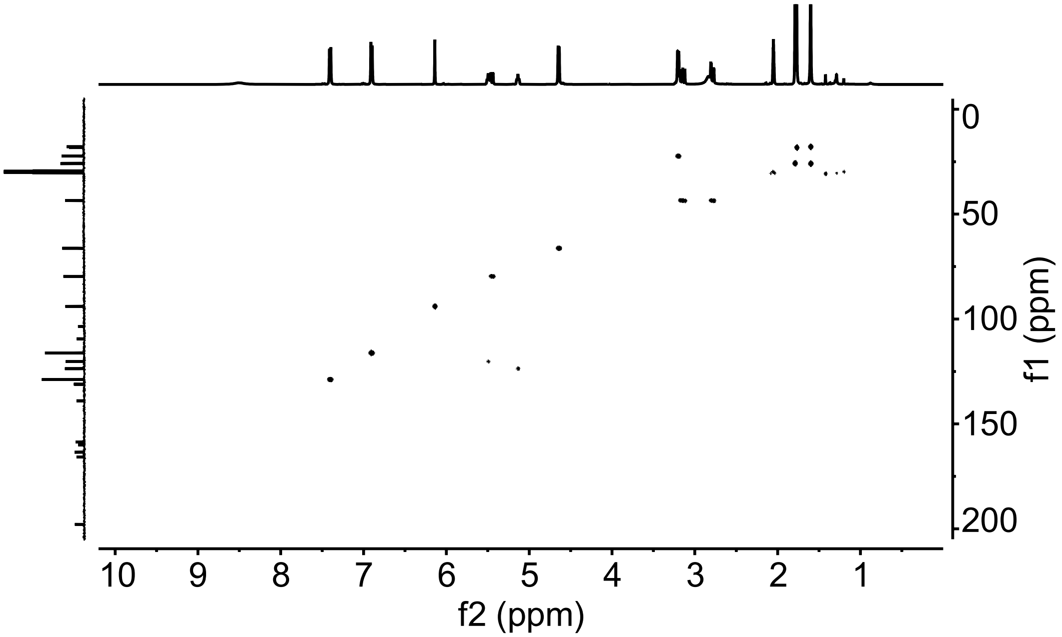

Figure S30. ^{1}H (500 MHz) and ^{13}C (125 MHz) NMR spectra and HMQC correlations of 8-*C*,7-*O*-diprenylnaringenin (**12**) in acetone-*d6*.

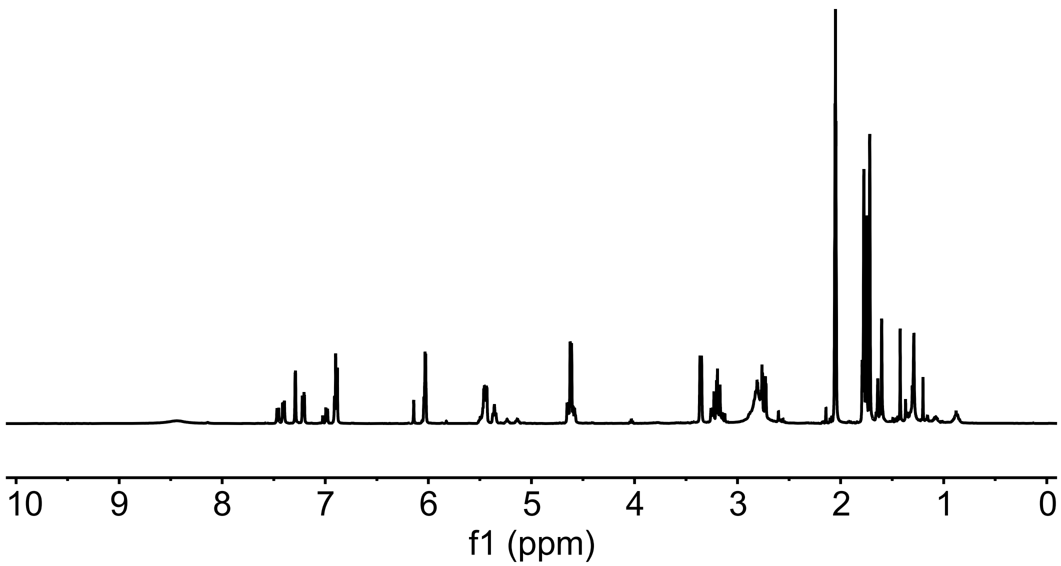

Figure S31. ^{1}H (500 MHz) NMR of 3’-*C*,7-*O*-diprenylnaringenin (**13**) in acetone-*d6*.

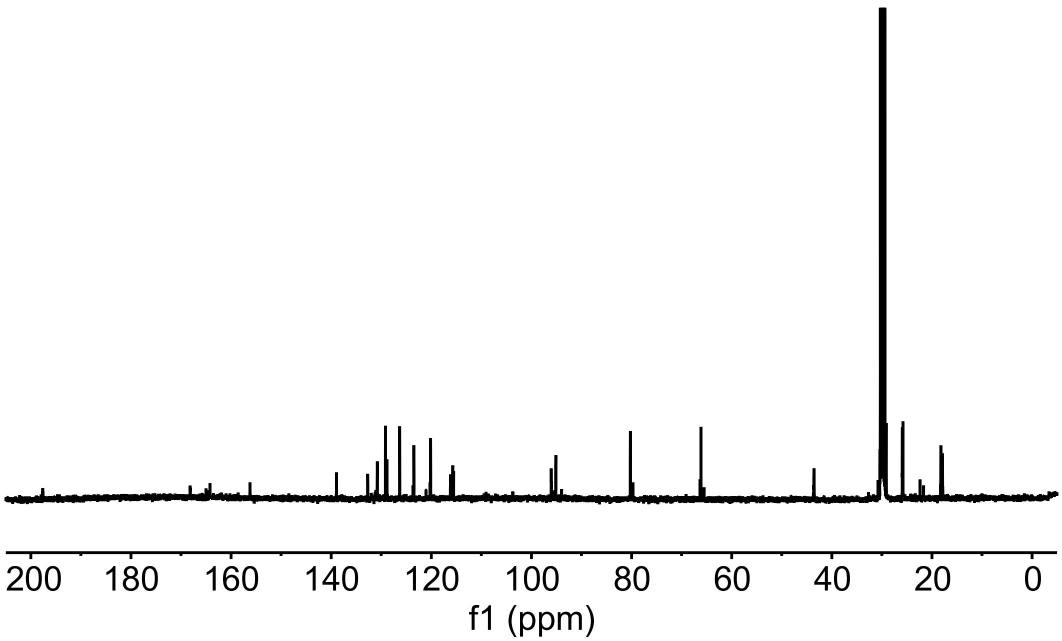

Figure S32. ^{13}C NMR (125 MHz) of 3’-*C*,7-*O*-diprenylnaringenin (**13**) in acetone-*d6*.

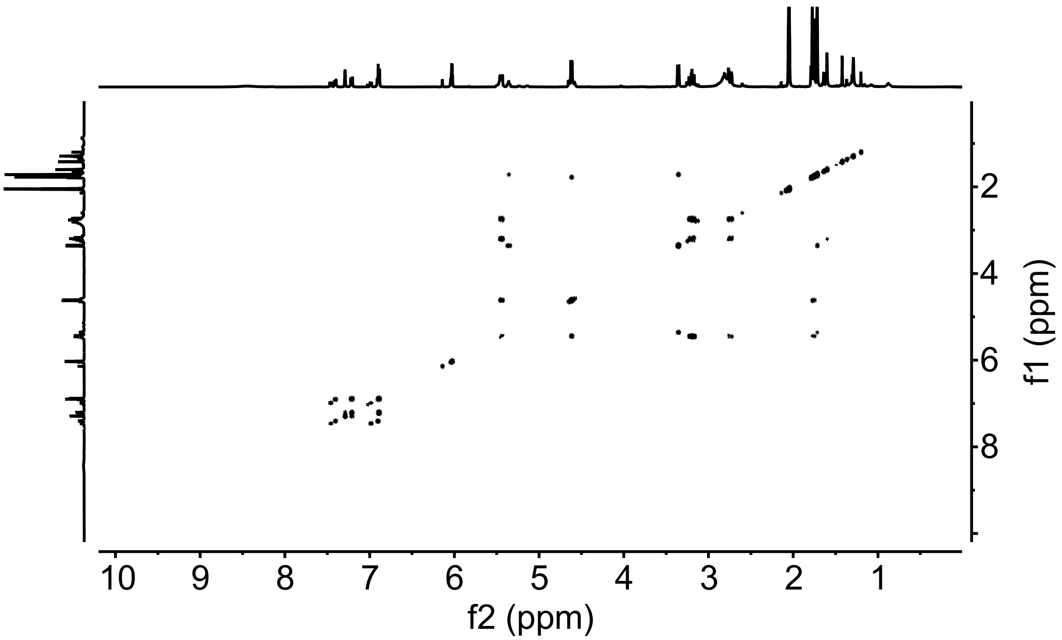

Figure S33. ^{1}H (500 MHz) NMR spectra and COSY correlations of 3'-*C*,7-*O*-diprenylnaringenin (**13**) in acetone-*d6*.

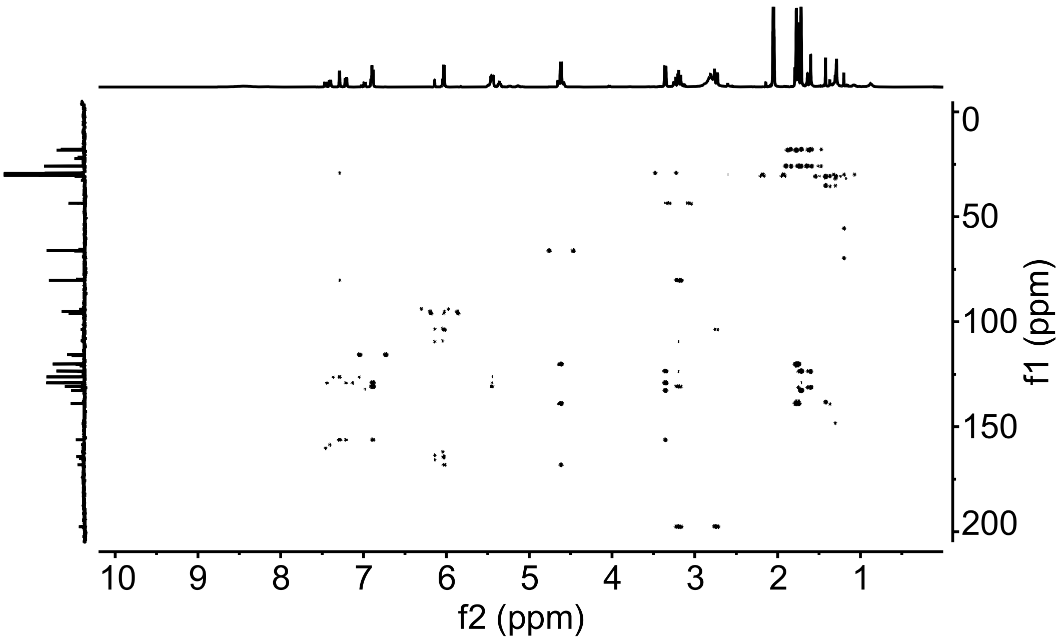

Figure S34. ^{1}H (500 MHz) and ^{13}C (125 MHz) NMR spectra and HMBC correlations of 3'-*C*,7-*O*-diprenylnaringenin (**13**) in acetone-*d6*.

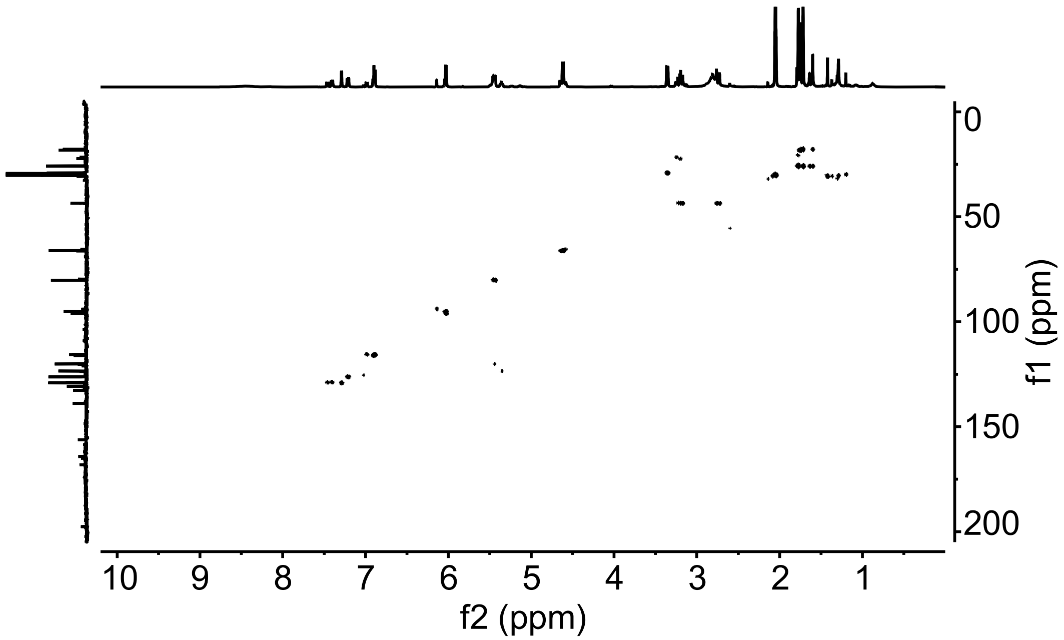

Figure S35. ^{1}H (500 MHz) and ^{13}C (125 MHz) NMR spectra and HMQC correlations of 3'-*C*,7-*O*-diprenylnaringenin (**13**) in acetone-*d6*.

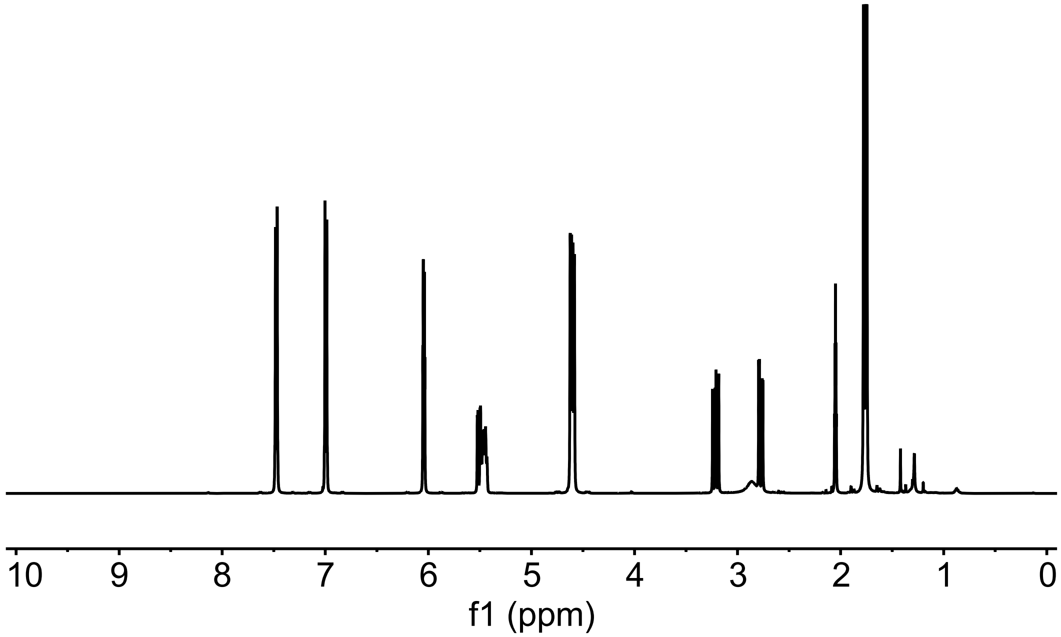

Figure S36. ^{1}H (500 MHz) NMR of 7,4-*O*-diprenylnaringenin (**14**) in acetone-*d6*.

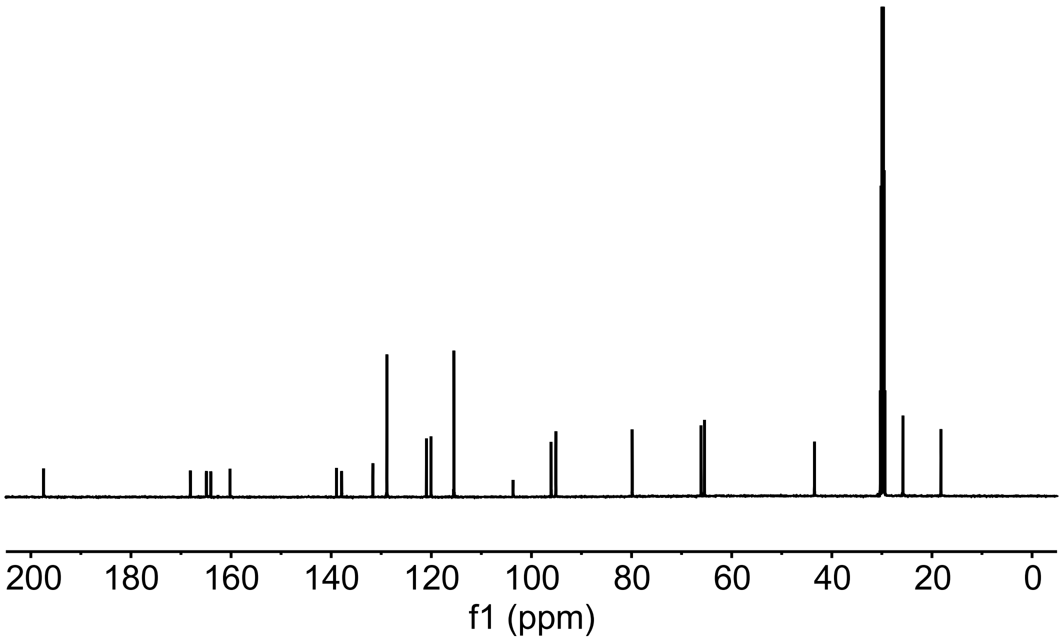

Figure S37. ^{13}C NMR (125 MHz) of 7,4-*O*-diprenylnaringenin (**14**) in acetone-*d6*.

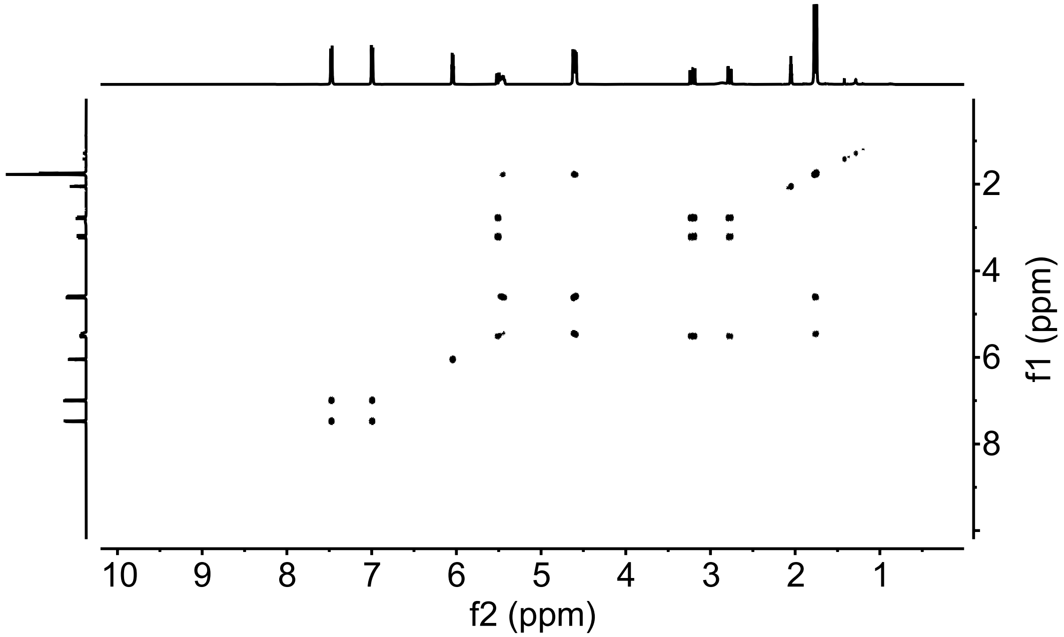

Figure S38. ^{1}H (500 MHz) NMR spectra and COSY correlations of 7,4-*O*-diprenylnaringenin (**14**) in acetone-*d6*.

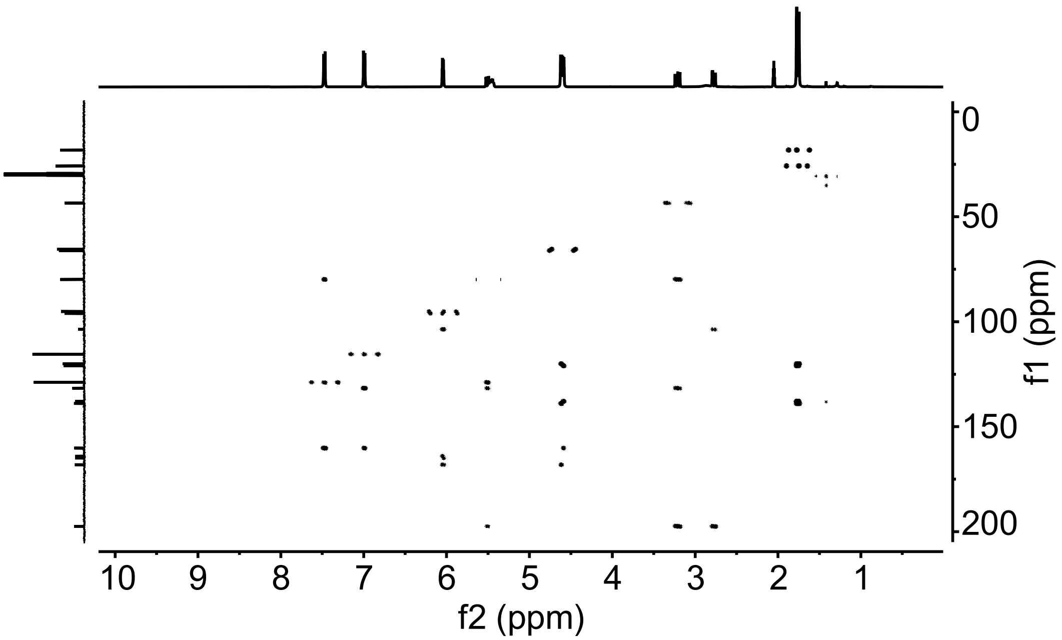

Figure S39. ^{1}H (500 MHz) and ^{13}C (125 MHz) NMR spectra and HMBC correlations of 7,4-*O*-diprenylnaringenin (**14**) in acetone-*d6*.

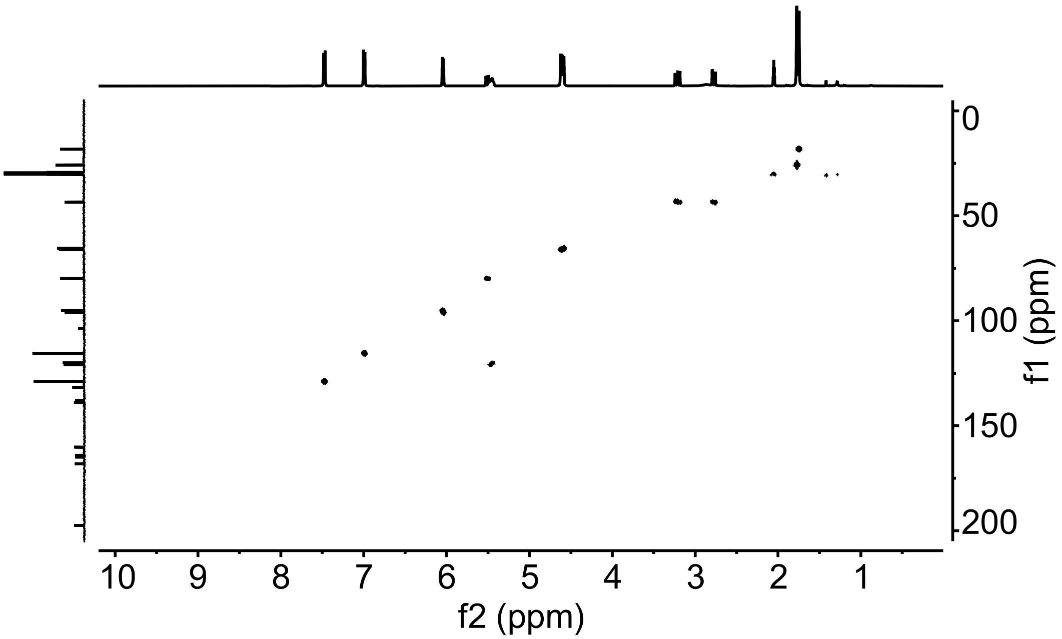

Figure S40. ^{1}H (500 MHz) and ^{13}C (125 MHz) NMR spectra and HMQC correlations of 7,4-*O*-diprenylnaringenin (**14**) in acetone-*d6*.

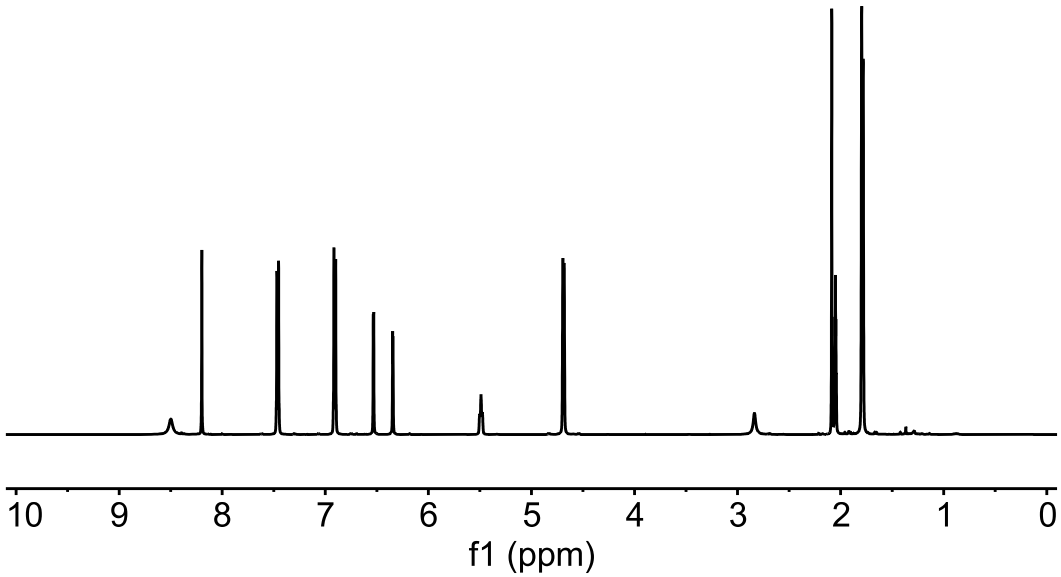

Figure S41. ^{1}H (500 MHz) NMR of 7-*O*-prenylgenistein (**34**) in acetone-*d6*.

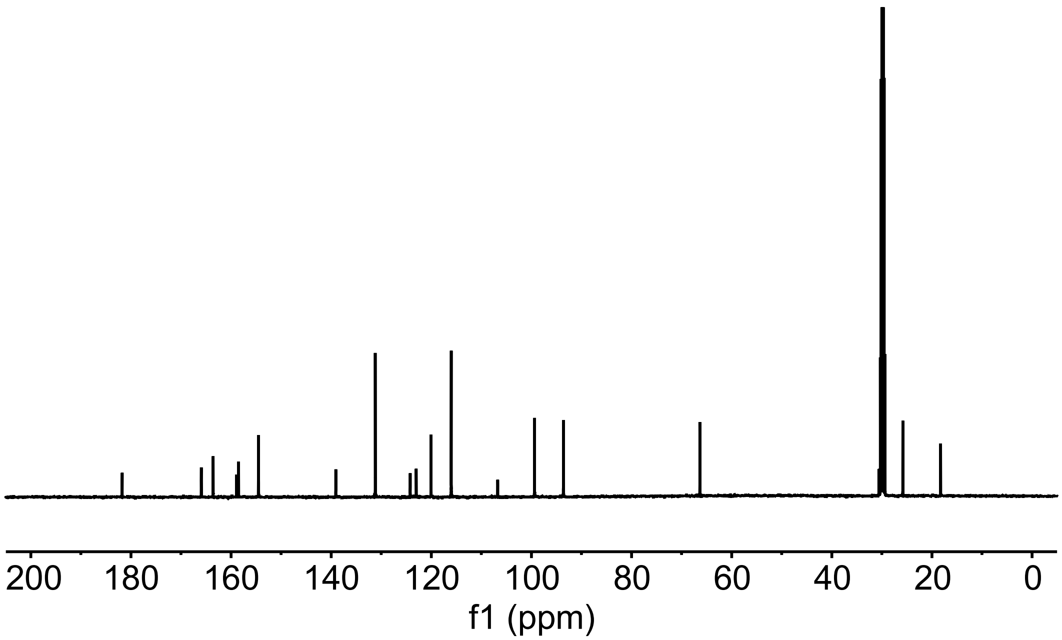

Figure S42. ^{13}C NMR (125 MHz) of 7-*O*-prenylgenistein (**34**) in acetone-*d6*.

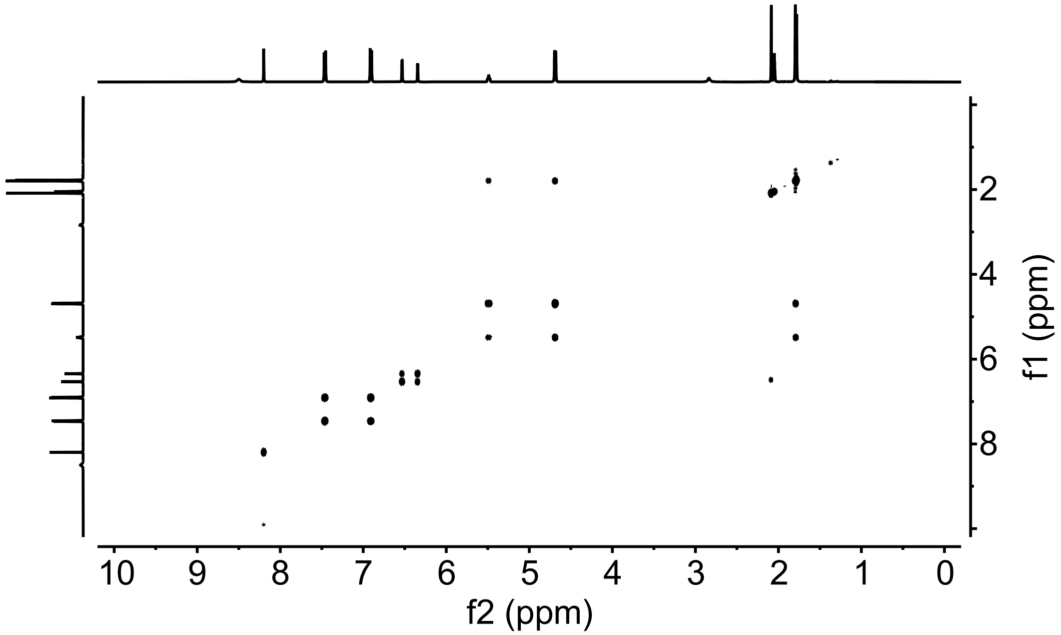

Figure S43. ^{1}H (500 MHz) NMR spectra and COSY correlations of 7-*O*-prenylgenistein (**34**) in acetone-*d6*.

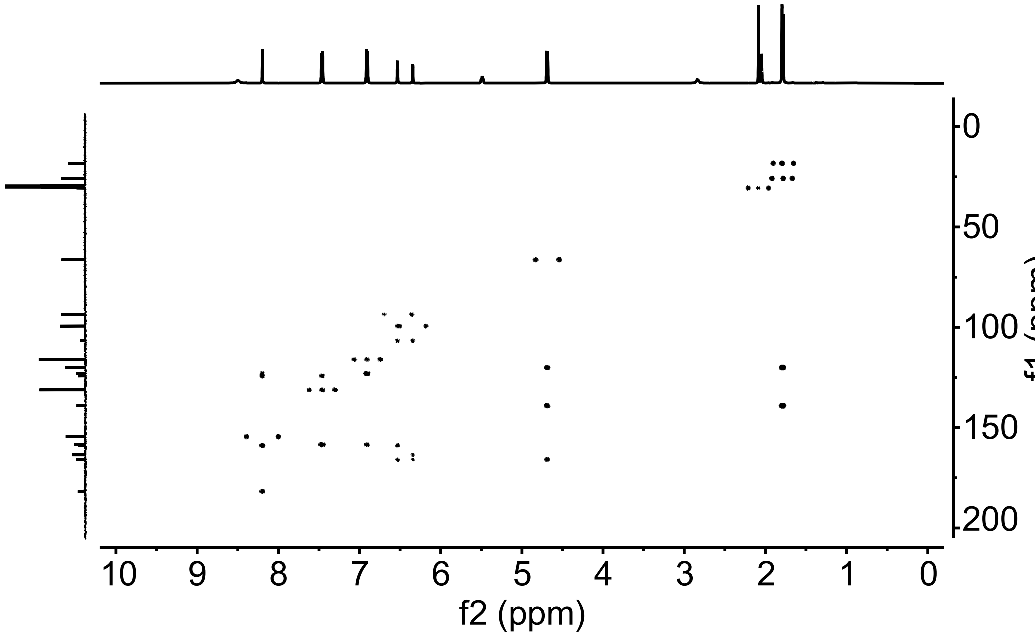

Figure S44. ^{1}H (500 MHz) and ^{13}C (125 MHz) NMR spectra and HMBC correlations of 7-*O*-prenylgenistein (**34**) in acetone-*d6*.

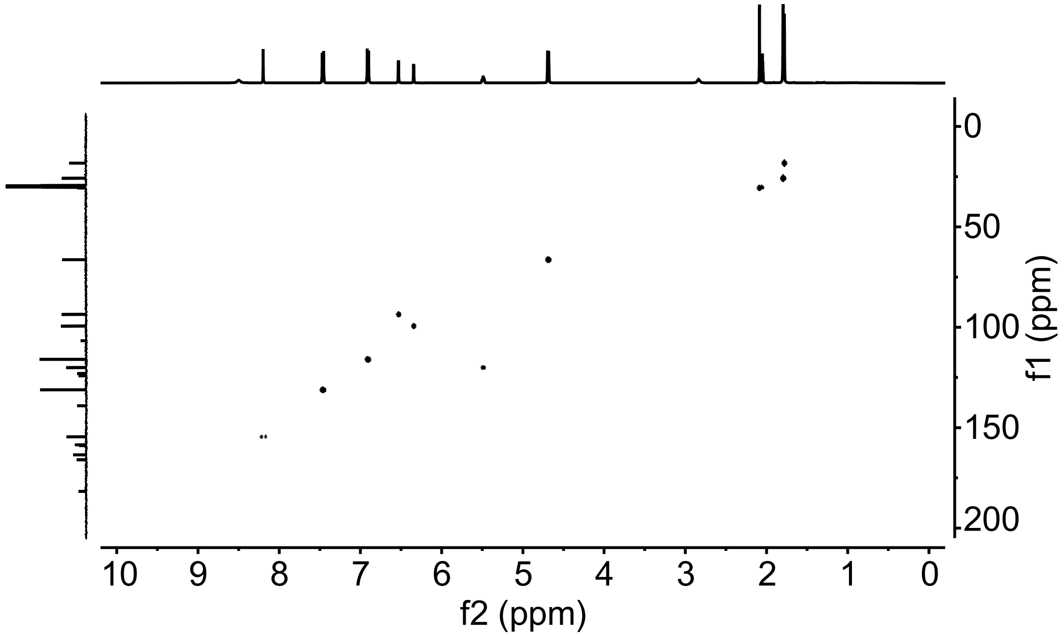

Figure S45. ^{1}H (500 MHz) and ^{13}C (125 MHz) NMR spectra and HMQC correlations of 7-*O*-prenylgenistein (**34**) in acetone-*d6*.

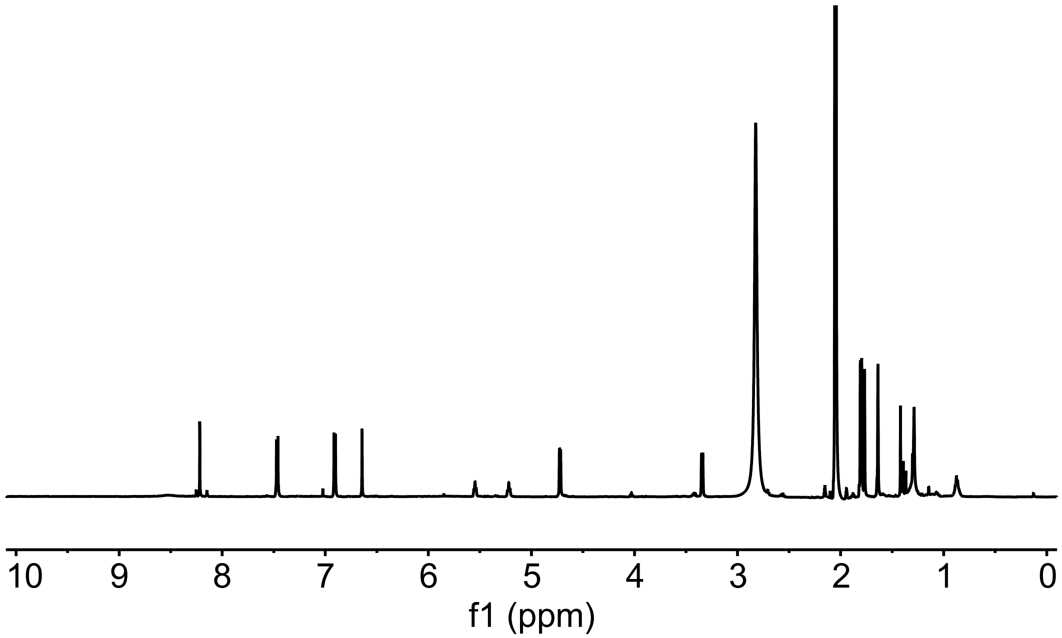

Figure S46. ^{1}H (500 MHz) NMR of 6-*C*,7-*O*-diprenylgenistein (**35**) in acetone-*d6*.

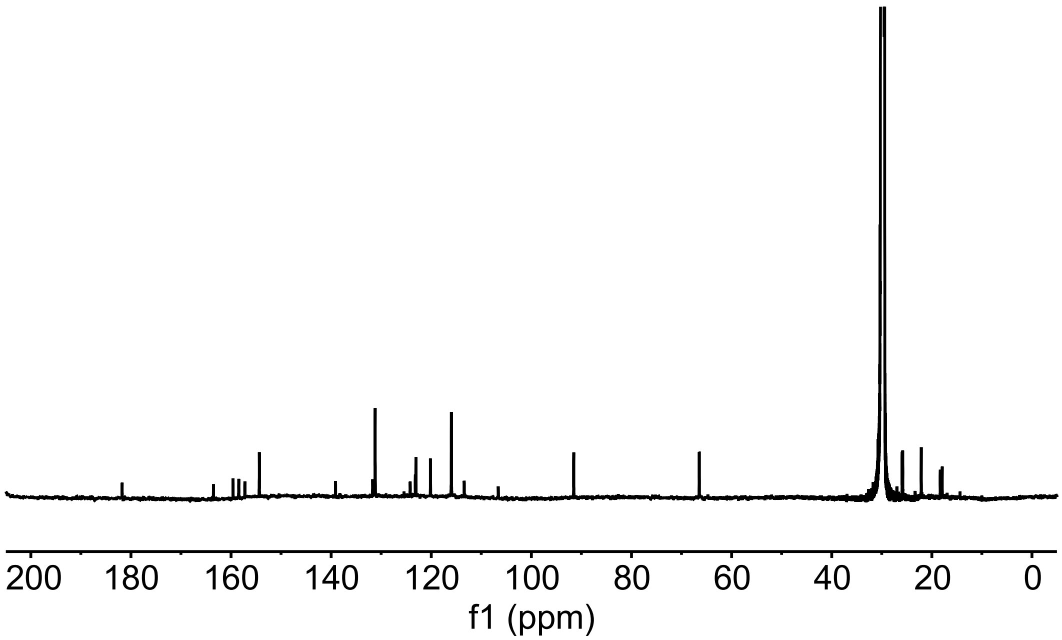

Figure S47. ^{13}C NMR (125 MHz) of 6-*C*,7-*O*-diprenylgenistein (**35**) in acetone-*d6*.

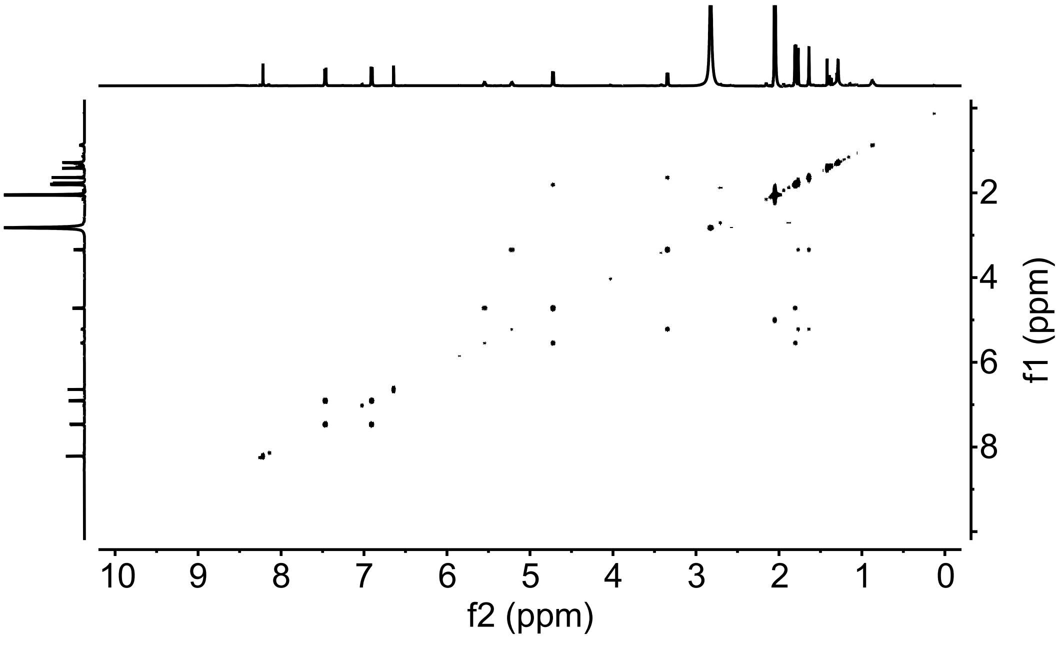

Figure S48. ^{1}H (500 MHz) NMR spectra and COSY correlations of 6-*C*,7-*O*-diprenylgenistein (**35**) in acetone-*d6*.

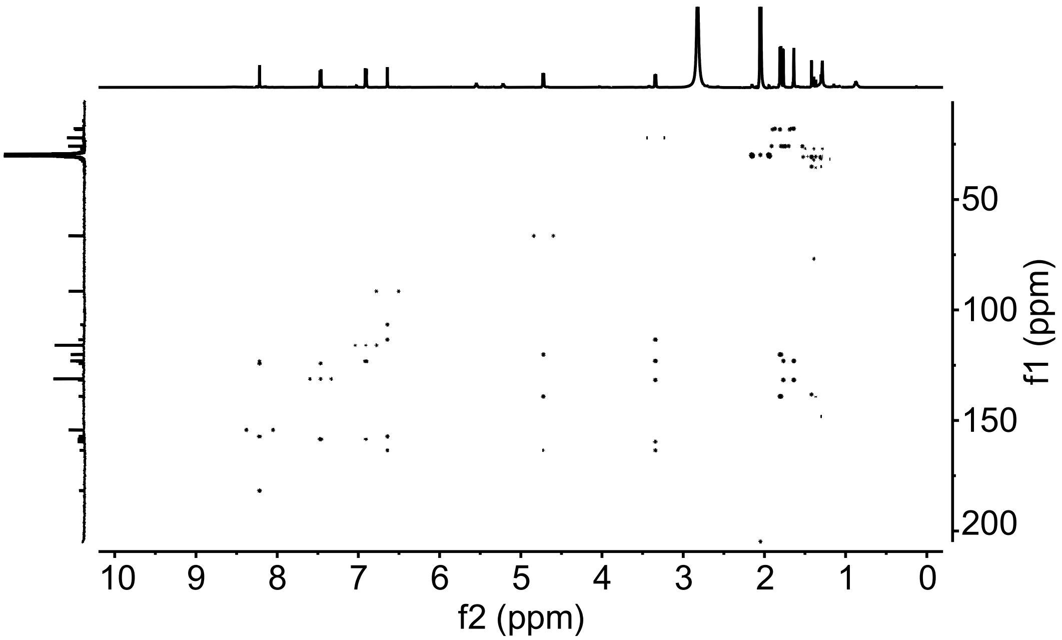

Figure S49. ^{1}H (500 MHz) and ^{13}C (125 MHz) NMR spectra and HMBC correlations of 6-*C*,7-*O*-diprenylgenistein (**35**) in acetone-*d6*.

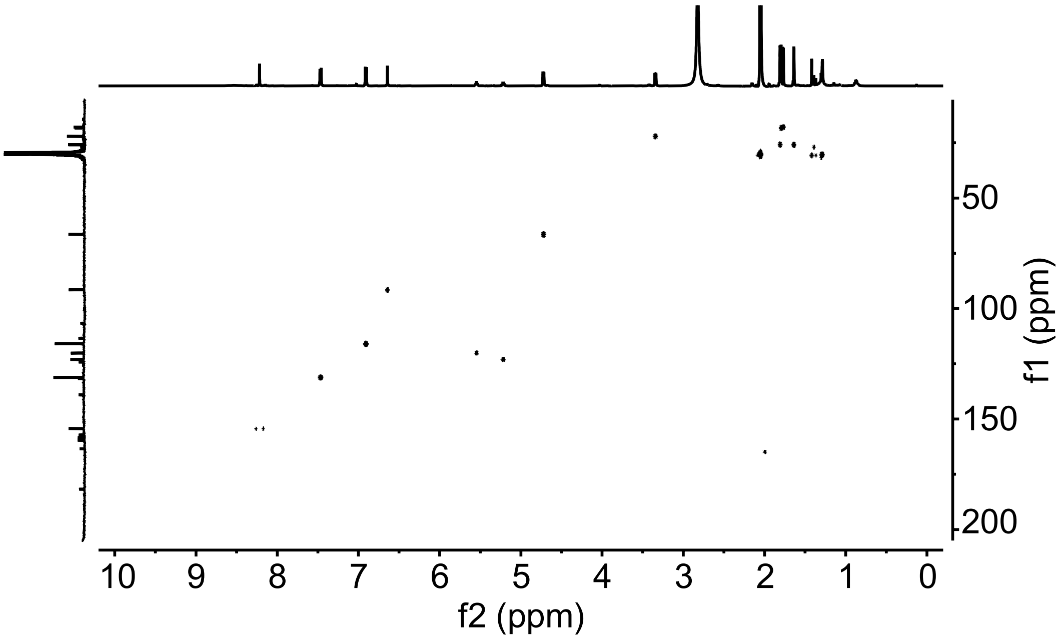

Figure S50. ^{1}H (500 MHz) and ^{13}C (125 MHz) NMR spectra and HSQC correlations of 6-*C*,7-*O*-diprenylgenistein (**35**) in acetone-*d6*.

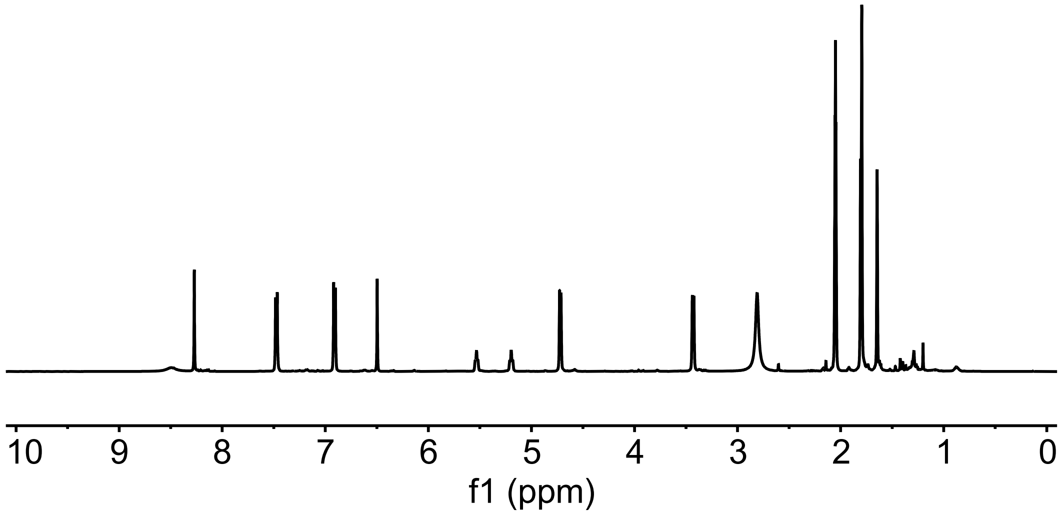

Figure S51. ^{1}H (500 MHz) NMR of 8-*C*,7-*O*-diprenylgenistein (**36**) in acetone-*d6*.

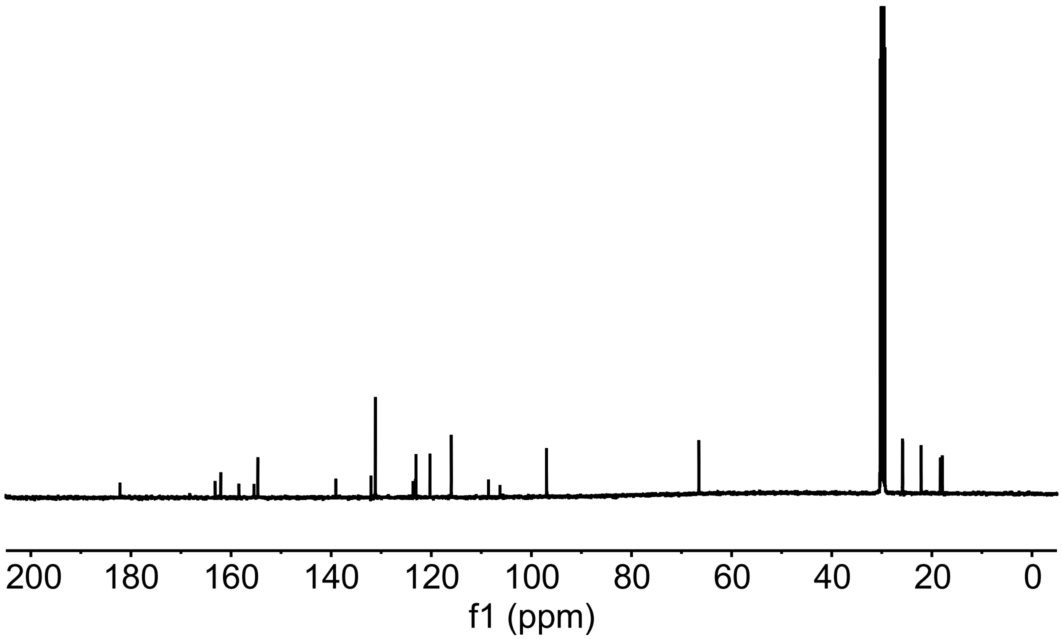

Figure S52. ^{13}C NMR (125 MHz) of 8-*C*,7-*O*-diprenylgenistein (**36**) in acetone-*d6*.

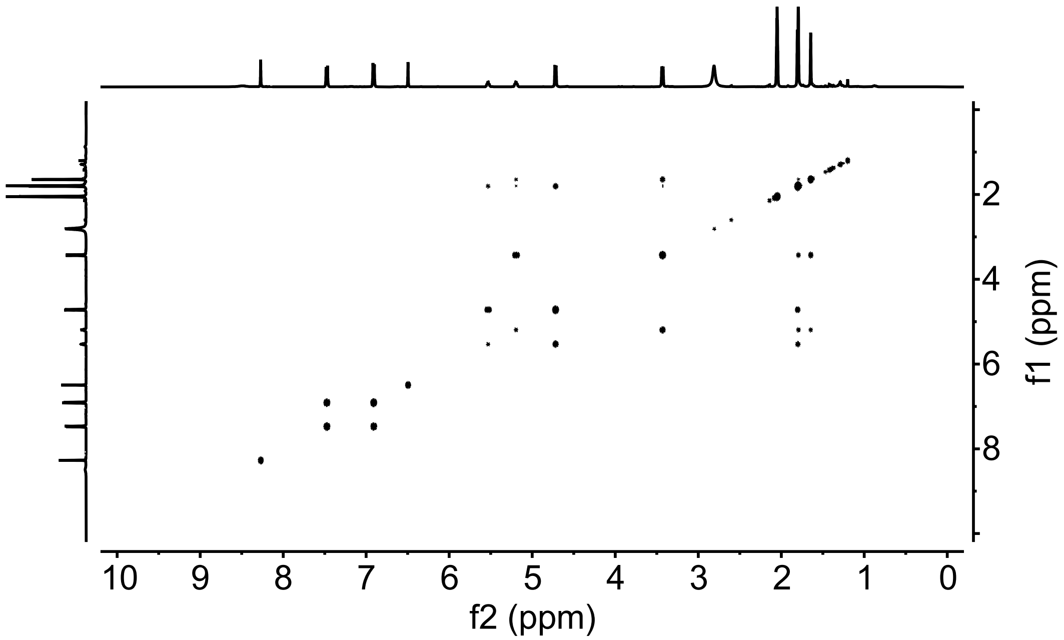

Figure S53. ^{1}H (500 MHz) NMR spectra and COSY correlations of 8-*C*,7-*O*-diprenylgenistein (**36**) in acetone-*d6*.

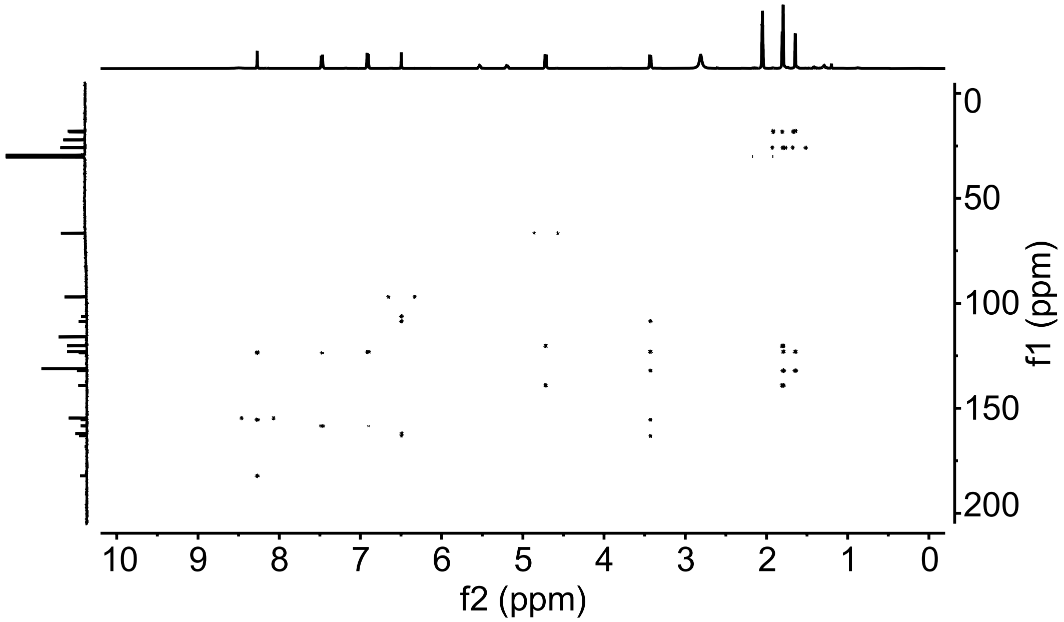

Figure S54. ^{1}H (500 MHz) and ^{13}C (125 MHz) NMR spectra and HMBC correlations of 8-*C*,7-*O*-diprenylgenistein (**36**) in acetone-*d6*.

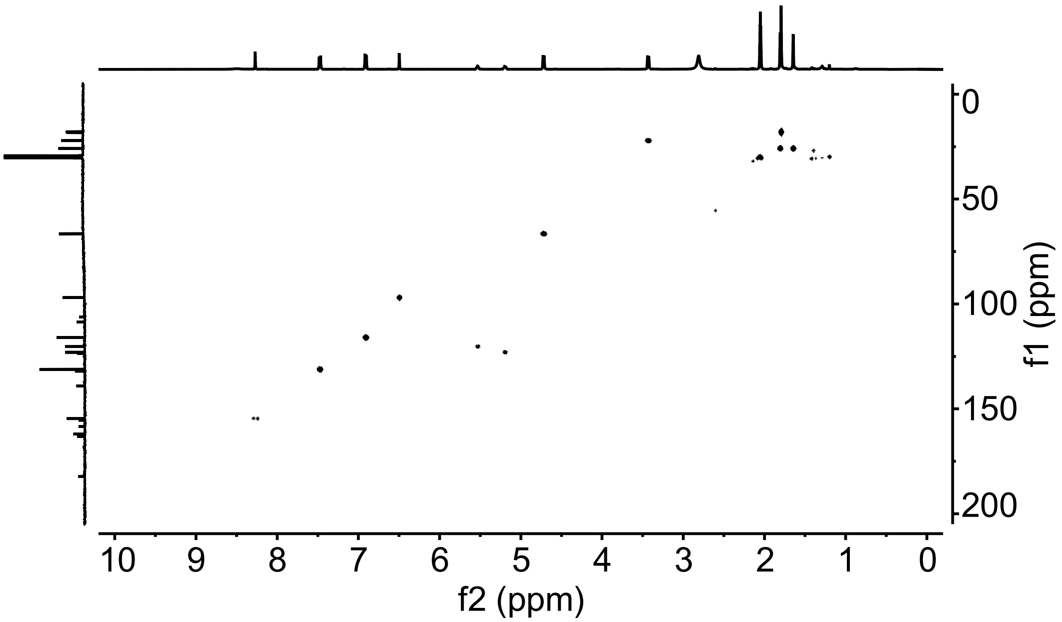

Figure S55. ^{1}H (500 MHz) and ^{13}C (125 MHz) NMR spectra and HMQC correlations of 8-*C*,7-*O*-diprenylgenistein (**36**) in acetone-*d6*.

Supporting information B

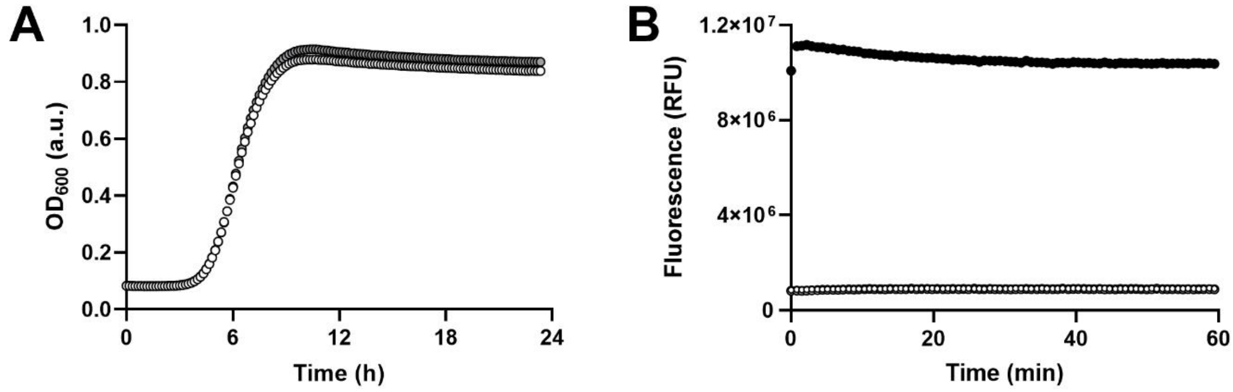

Figure S56. The signal for the controls during the broth microdilution assay and propidium iodide (PI) uptake assay based on three biological replicates. **A)** Optical density (OD) at 600 nm in arbitrary units (a.u.) in the broth microdilution assay for negative controls tryptone soy broth (TSB, grey circles) and 1% (*v/v*) dimethylsulfoxide (DMSO) in TSB (white circles). **B)** Fluorescence in relative fluorescence units (RFU) in the PI uptake assay of negative controls peptone physiological salt solution (PPS, grey circles) and 2% (*v/v*) dimethylsulfoxide (DMSO) in PPS (white circles), and maximum fluorescence by heated cells (black circles).

Table S3. Growth delay (GD) of MRSA 18HN by prenylated phenolics inactive at 50 μg mL^{-1}. Structures of prenylated phenolics can be found in **Figure 1**. The growth delay (GD) was calculated by subtracting time-to-detection (TTD), *i.e.*, the time to reach a change in optical density at 600 nm of 0.05 units, of the DMSO blank from the TTD of the compound.

**Compound**	**GD at 50 μg mL^{-1} in hours (average ± st. dev.)**
Bavachinin (**1**)	13.5 ± 1.4
Isobavachin (**3**)	8.4 ± 2.8
3'-Prenylnaringenin (**7**)	3.8 ± 0.4
Isoxanthohumol (**9**)	> 24
6-*C*,7-*O*-Diprenylnaringenin (**11**)	19.0 ± 0.9
8-*C*,7-*O*-Diprenylnaringenin (**12**)	1.5 ± 0.8
3'-*C*,7-*O*-Diprenylnaringenin (**13**)	0.4 ± 0.2
7,4'-*O*-Diprenylnaringenin (**14**)	0.6 ± 0.8
Licorisoflavan A (**16**)	3.4 ± 1.5
α-Isowighteone (**24**)	1.9 ± 0.0
7-*O*-Prenylgenistein (**34**)	-0.7 ± 0.2
6-*C*,7-*O*-Diprenylgenistein (**35**)	5.3 ± 0.2
8-*C*,7-*O*-Diprenylgenistein (**36**)	4.8 ± 0.5

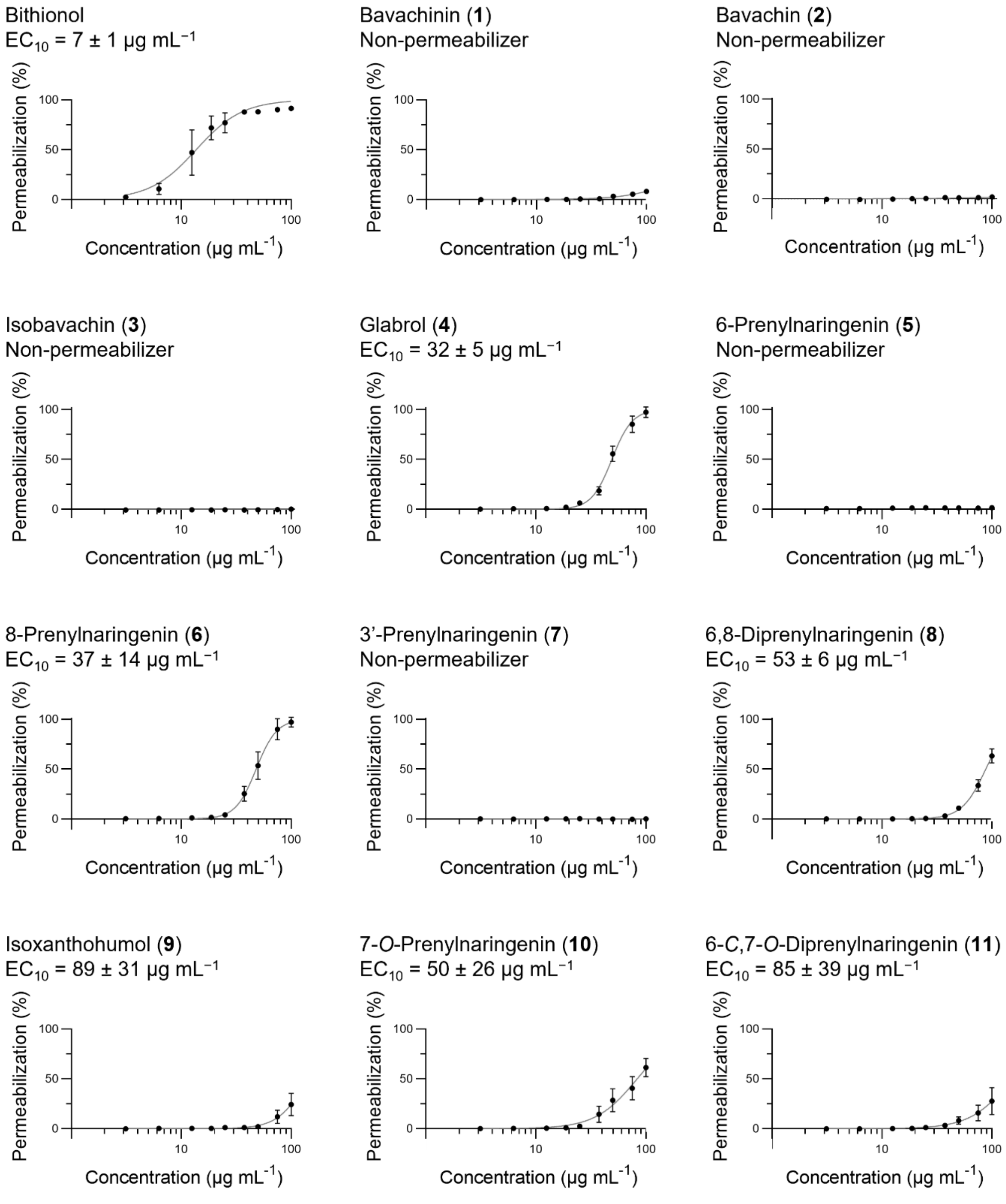

Figure S57. Effective concentrations for 10% of maximum membrane permeabilization (EC$_{10}$) and concentration-response curves for the positive control bithionol and prenylated phenolics **1 – 36.** Data points show the average membrane permeabilization (% compared to maximum fluorescence control, *i.e.*, heated cells), and error bars represent the standard deviation of the mean of biological replicates ($n \geq 3$). The EC$_{10}$ shown is the average ± standard deviation extracted from concentration-response curves of individual replicates.

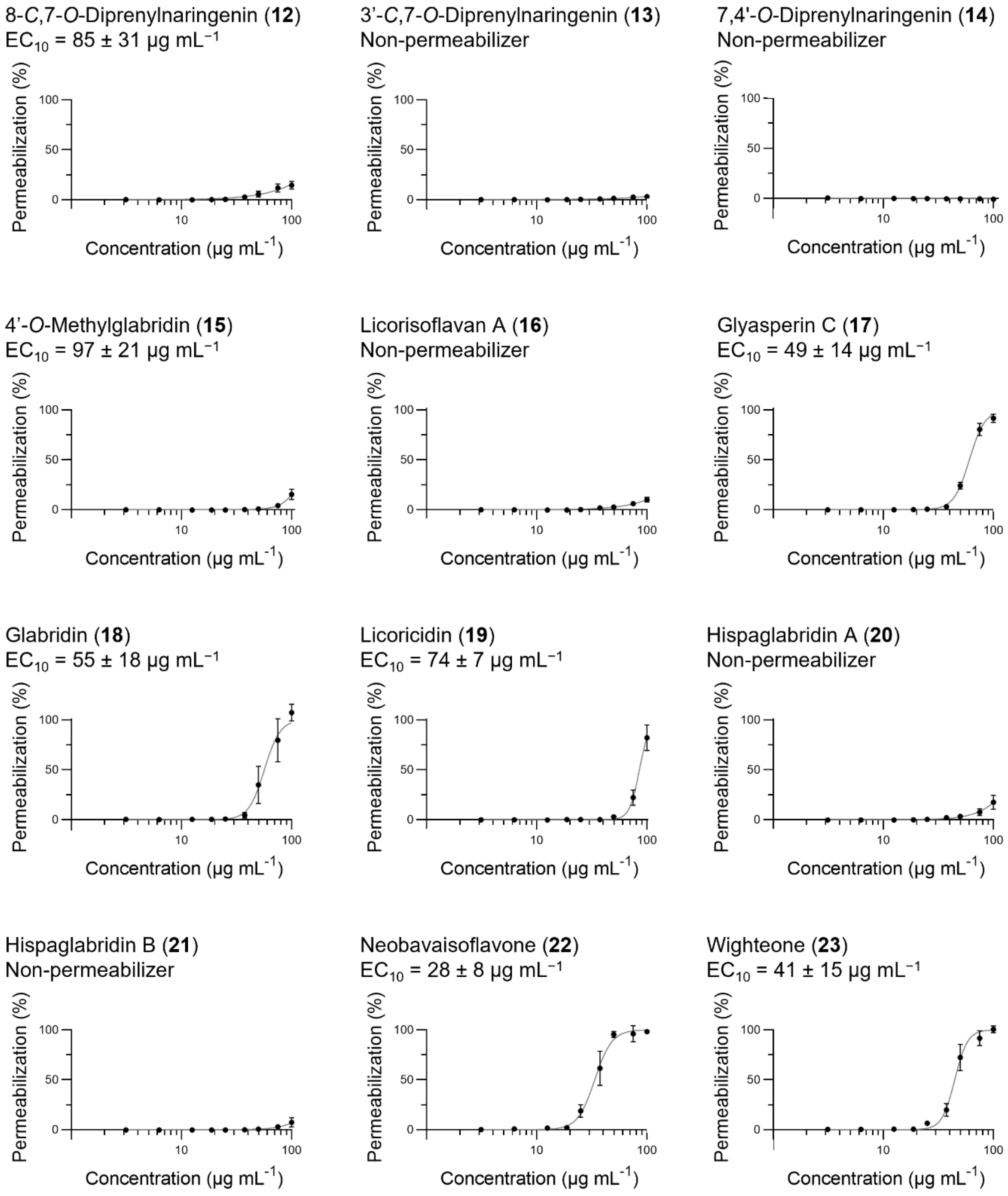

Figure S57. Continued.

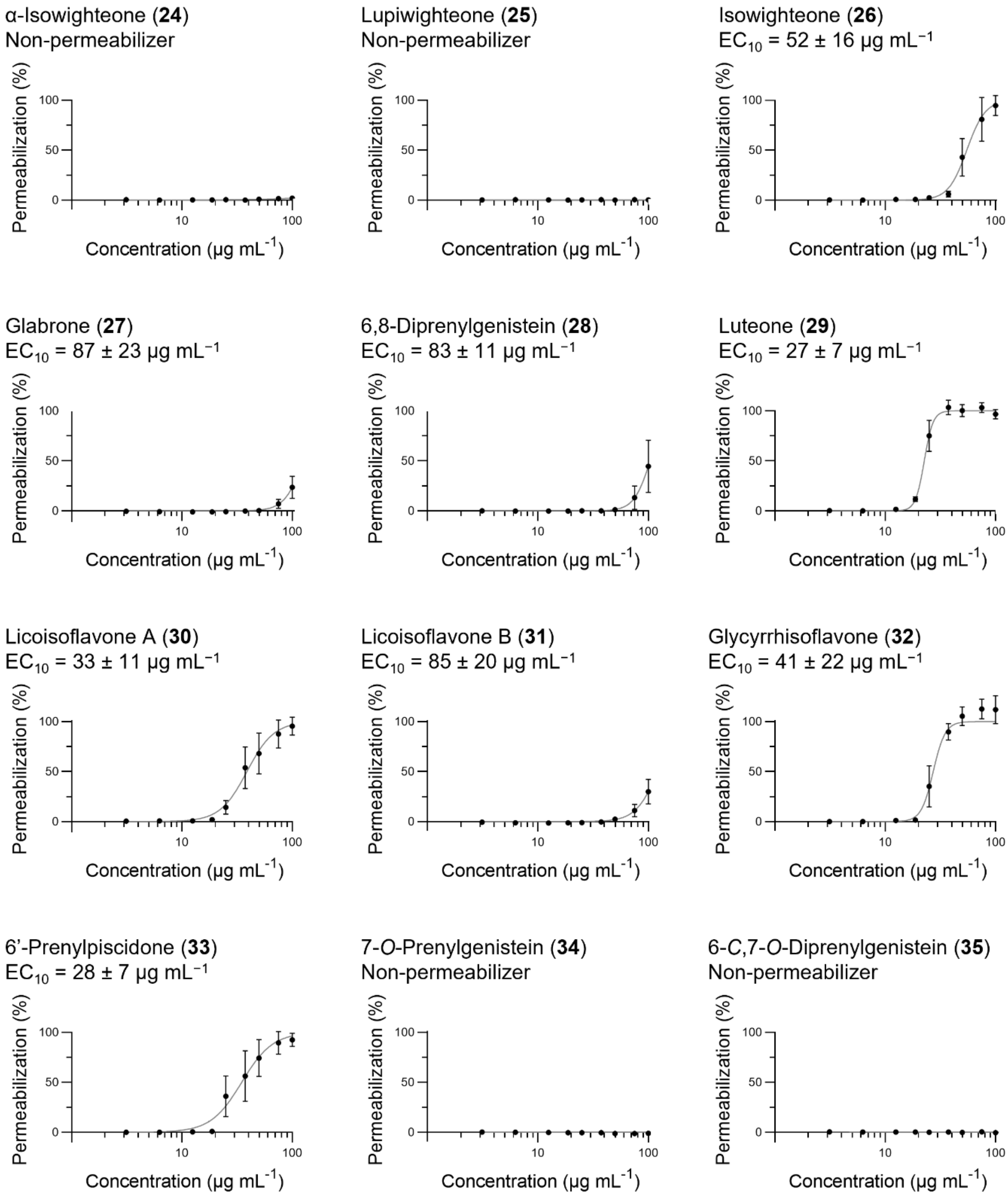

Figure S57. Continued.

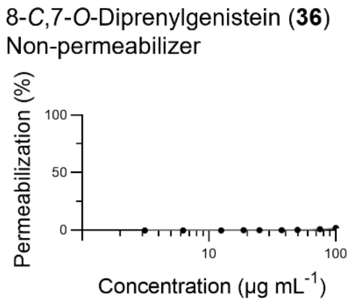

Figure S57. Continued.

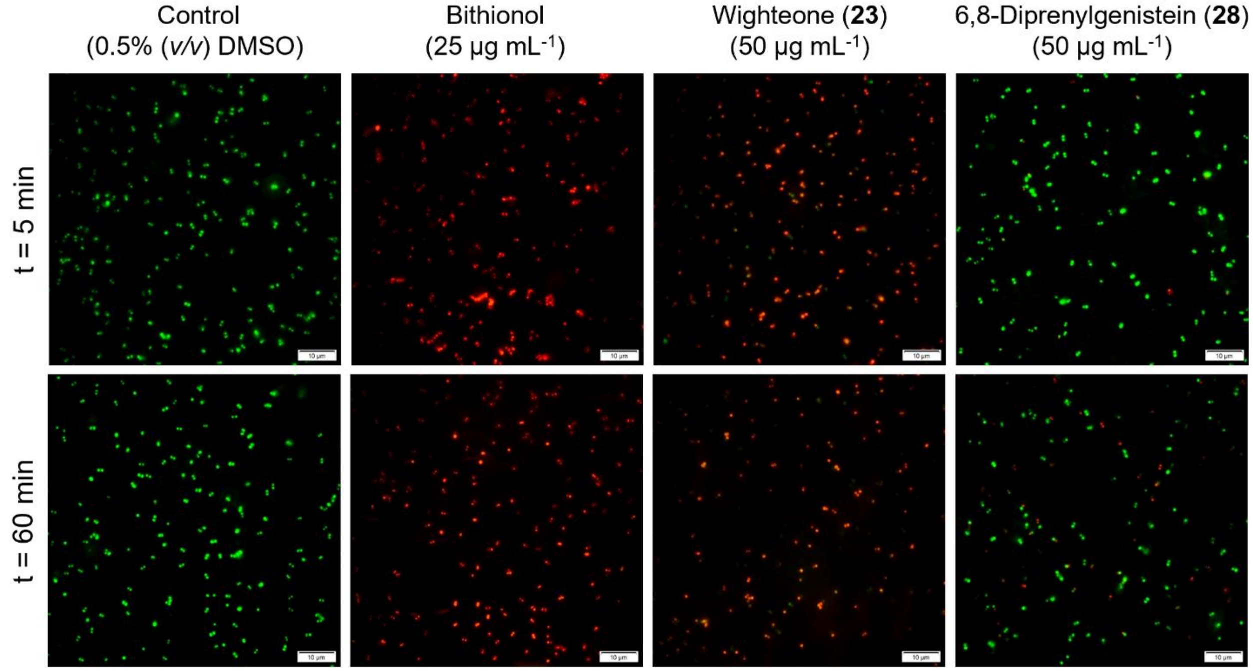

Figure S58. Fluorescence microscopy images of MRSA stained with propidium iodide (red, permeabilized) and SYTO 9 (green, not permeabilized) after 5 and 60 min of treatment with positive control bithionol and prenylated phenolics wighteone (**23**) and 6,8-diprenylgenistein (**28**). Scale bar: 10 μm.

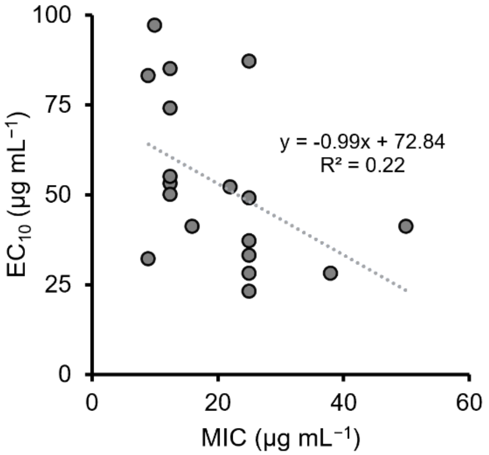

Figure S59. Correlation between the effective concentration for permeabilization (EC_{10}) and the antimicrobial activity (MIC) for all prenylated phenolics with antimicrobial (MIC ≤ 50 µg mL^{-1}) and permeabilizing (EC_{10} < 100 µg mL^{-1}) activity.

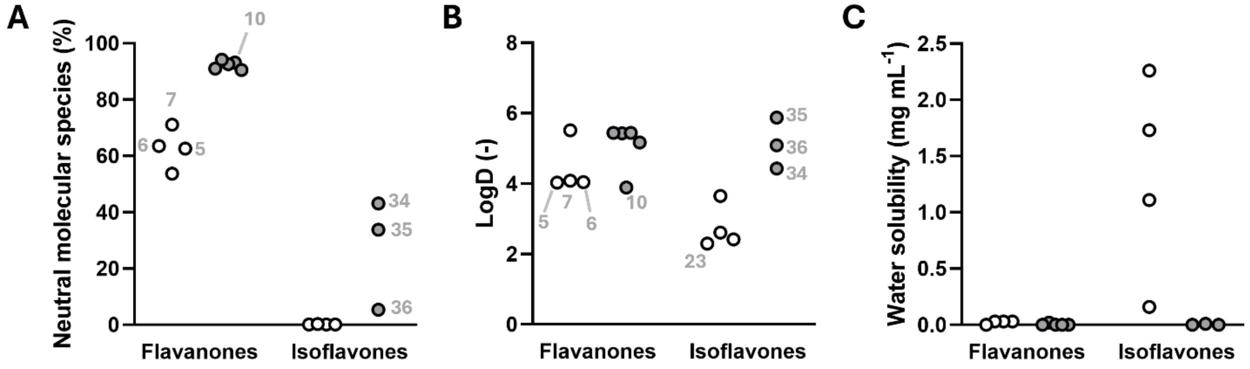

Figure S60. Calculated (by MarvinSketch) properties of *O*-prenylated phenolics (grey circles, flavanones **10**, **11**, **12**, **13**, **14**, and isoflavones **34**, **35**, **36**) and their *C*-prenylated counterparts (white circles, flavanones **5**, **6**, **7**, **8**, and isoflavones **23**, **25**, **26**, **28**). **A**) The neutral or undissociated molecular species (% at pH 7.3 at 310 K). **B**) Hydrophobicity (LogD at pH 7.3). **C**) Aqueous solubility (mg mL^{-1} at pH 7.3). Compound numbers refer to **Figure 1**.

Table S4. Calculated (by MarvinSketch) properties of prenylated phenolics: The neutral fraction (% of molecular species in undissociated form at pH 7.3 at 310 K), hydrophobicity (logD at pH 7.3), and solubility (aqueous solubility at pH 7.3).

Compound	Molecular species in undissociated form at pH 7.3 at 310 K (%)	LogD at pH 7.3	Aqueous solubility at pH 7.3 (mg mL^{-1})
**Flavanones**			
Bavachinin (**1**)	98.94	3.85	< 0.01
Bavachin (**2**)	59.38	3.64	0.01
Isobavachin (**3**)	59.53	3.64	0.01
Glabrol (**4**)	59.17	5.19	< 0.01
6-Prenylnaringenin (**5**)	62.59	4.03	0.03
8-Prenylnaringenin (**6**)	63.57	4.04	0.03
3'-Prenylnaringenin (**7**)	71.09	4.08	0.03
6,8-Diprenylnaringenin (**8**)	53.72	5.52	< 0.01
Isoxanthohumol (**9**)	62.76	3.41	0.01
7-*O*-Prenylnaringenin (**10**)	93.17	3.89	0.02
6-*C*,7-*O*-Diprenylnaringenin (**11**)	90.54	5.43	< 0.01
8-*C*,7-O-Diprenylnaringenin (**12**)	91.08	5.44	< 0.01
3'-*C*,7-*O*-Diprenylnaringenin (**13**)	92.62	5.44	< 0.01
7,4'-*O*-Diprenylnaringenin (**14**)	94.17	5.17	< 0.01
**Isoflavans**			
4'-*O*-Methylglabridin (**15**)	99.57	4.00	0.01
Licorisoflavan A (**16**)	98.77	5.81	< 0.01
Glyasperin C (**17**)	98.23	4.23	0.05
Glabridin (**18**)	99.21	3.97	0.02
Licoricidin (**19**)	98.17	5.78	< 0.01
Hispaglabridin A (**20**)	98.79	5.52	< 0.01
Hispaglabridin B (**21**)	99.48	5.00	< 0.01
**Isoflavones**			
Neobavaisoflavone (**22**)	0.92	2.65	0.15
Wighteone (**23**)	0.15	2.30	2.26
α-Isowighteone (**24**)	2.01	2.07	0.23
Lupiwighteone (**25**)	0.13	2.42	1.73
Isowighteone (**26**)	0.30	2.61	1.11
Glabrone (**27**)	0.88	1.83	0.18
6,8-Diprenylgenistein (**28**)	0.10	3.65	0.16
Luteone (**29**)	0.14	1.99	6.83
Licoisoflavone A (**30**)	0.27	2.29	3.42
Licoisoflavone B (**31**)	0.29	1.79	1.31
Glycyrrhisoflavone (**32**)	0.29	2.31	3.25
6'-Prenylpiscidone (**33**)	0.29	3.61	0.20
7-*O*-Prenylgenistein (**34**)	43.11	4.43	0.01
6-*C*,7-*O*-Diprenylgenistein (**35**)	33.76	5.88	< 0.01
8-*C*,7-*O*-Diprenylgenistein (**36**)	5.40	5.09	< 0.01

References

(1) van Dinteren, S.; Ritsema, J. H.; Sanders, M. G.; Meijerink, J.; Vincken, J.-P.; Araya-Cloutier, C. Unraveling the molecular drivers of antibacterial prenylated (iso)flavonoids and chalcones against *Streptococcus mutans*. *Sci Rep.* **2025**, *15* (1), 14776. DOI: 10.1038/s41598-025-98782-7.
(2) van de Schans, M. G. M.; Ritschel, T.; Bovee, T. F. H.; Sanders, M. G.; de Waard, P.; Gruppen, H.; Vincken, J.-P. Involvement of a hydrophobic pocket and helix 11 in determining the modes of action of prenylated flavonoids and isoflavonoids in the human estrogen receptor. *ChemBioChem.* **2015**, *16* (18), 2668–2677. DOI: 10.1002/cbic.201500343.
